# Astrocytic LRRK2 Controls Synaptic Connectivity via Regulation of ERM Phosphorylation

**DOI:** 10.1101/2023.04.09.536178

**Published:** 2024-08-28

**Authors:** Shiyi Wang, Ryan Baumert, Gabrielle Séjourné, Dhanesh Sivadasan Bindu, Kylie Dimond, Kristina Sakers, Leslie Vazquez, Jessica Moore, Christabel Xin Tan, Tetsuya Takano, Maria Pia Rodriguez, Scott H. Soderling, Albert R. La Spada, Cagla Eroglu

**Affiliations:** 1 The Department of Cell Biology, Duke University Medical Center, Durham, NC, USA; 2 Department of Neonatology, Children’s Mercy Hospital, Kansas City, MO, USA; 3 College of Psychology, Nova Southeastern University, Fort Lauderdale, FL, USA; 4 The Department of Neurobiology, Duke University Medical Center, Durham, NC, USA; 5 Life Sciences Institute, University of Michigan, Ann Arbor, MI, USA; 6 Division of Molecular Systems for Brain Function, Kyushu University Institute for Advanced Study, Medical Institute of Bioregulation, Japan; 7 Japan Science and Technology Agency, PRESTO, Japan; 8 The Department of Neurobiology, Duke University Medical Center, Durham, NC, USA; 9 Aligning Science Across Parkinson’s (ASAP) Collaborative Research Network, Chevy Chase, MD; 10 The Department of Neurology, Duke University Medical Center, Durham, NC, USA; 11 Departments of Pathology & Laboratory Medicine, Neurology, Biological Chemistry, and Neurobiology & Behavior, University of California, Irvine, CA, USA; 12 UCI Center for Neurotherapeutics, University of California, Irvine, CA, USA; 13 Howard Hughes Medical Institute, Duke University Medical Center, Durham, NC, USA

## Abstract

Astrocytes, a major glial cell type of the brain, regulate synapse numbers and function. However, whether astrocyte dysfunction can cause synaptic pathologies in neurological disorders such as Parkinson’s Disease (PD) is unknown. Here, we investigated the impact of the most common PD-linked mutation in the leucine-rich repeat kinase 2 (*LRRK2*) gene (G2019S) on the synaptic functions of astrocytes. We found that both in human and mouse cortex, the LRRK2 G2019S mutation causes astrocyte morphology deficits and enhances the phosphorylation of the ERM proteins (Ezrin, Radixin, and Moesin), which are important components of perisynaptic astrocyte processes. Reducing ERM phosphorylation in LRRK2 G2019S mouse astrocytes restored astrocyte morphology and corrected excitatory synaptic deficits. Using an *in vivo* BioID proteomic approach, we found Ezrin, the most abundant astrocytic ERM protein, interacts with the Autophagy-Related 7 (Atg7), a master regulator of catabolic processes. The Ezrin/Atg7 interaction is inhibited by Ezrin phosphorylation, thus diminished in the LRRK2 G2019S astrocytes. Importantly, Atg7 function is required to maintain proper astrocyte morphology. These studies reveal an astrocytic molecular mechanism that could serve as a therapeutic target in PD.

## INTRODUCTION

Parkinson’s Disease (PD) is a progressive neurodegenerative motor disorder affecting over 8 million people worldwide^1^. PD is commonly diagnosed at the onset of motor symptoms, including tremors, bradykinesia, and rigidity^1^. By the time these motor symptoms are apparent, PD patients have already lost many of their dopaminergic neurons in the substantia nigra pars compacta (SNPc)^2^. However, decades before the onset of motor dysfunction, individuals with PD often display non-motor “prodromal” symptoms, such as cognitive, mood, and sleep disorders, suggesting that early synaptic circuit dysfunction exists across multiple brain regions, including the cerebral cortex^3,4^. Even though most PD cases are idiopathic, ~15% are caused by mutations in Parkinsonism genes^5^. The PD-linked genes identified to date point to dysfunction in fundamental cellular processes such as lysosome and mitochondria function, vesicular trafficking, and secretion^6^. However, how the dysfunction of these genes results in impaired synaptic circuits and dopaminergic neuron death is unknown. Moreover, many cell types in the brain and body express these PD-linked genes^7^. Thus, the cellular origins of PD may not be restricted to neurons.

Astrocytes instruct the formation and function of synaptic circuits^8,9^. Most synapses in the central nervous system are in direct contact with perisynaptic astrocyte processes (PAPs)^10^. This structural association is essential for regulating synaptic function; therefore, the PAPs and the presynaptic and postsynaptic neuronal compartments are collectively termed “the tripartite synapse”^10^. At the tripartite synapse, astrocytes clear excess neurotransmitters, maintain ion homeostasis, and secrete factors regulating synapse development and function^11^. To accomplish these crucial roles at the synapse, astrocytes gain a highly elaborate morphology and form a network coupled through gap junctions to continuously tile the entire brain parenchyma^8,12,13^. Previous studies found that astrocyte morphogenesis, tiling, and neuronal synaptogenesis are interdependent processes controlled by bidirectional signaling via secreted factors and cell adhesion molecules^14,15^. Recent studies also found that astrocytes become reactive, undergoing morphological and molecular changes in numerous neurological disorders, including PD^16,17^.

Mutations in the leucine-rich repeat kinase 2 (*LRRK2*) gene cause familial PD. Lrrk2 is a large multidomain protein containing ROC (Ras of complex), COR (C-terminal of ROC), catalytic kinase, and four protein-protein interactions domains (armadillo, ankyrin, leucine-rich repeats, WD40 domains)^18,19,^. Lrrk2 plays critical roles in ciliogenesis, lysosome function, immune system, and synaptic vesicle trafficking by phosphorylation of its substrates^20–26^. LRRK2 G2019S is the most common mutation that causes familial PD^20,27,28^. Long before the death of dopaminergic neurons, PD patients with the G2019S mutation have non-motor symptoms, such as depression, hallucinations, and sleep and cognitive disorders^29^. Several rodent models with LRRK2 G2019S mutation do not show motor dysfunction or dopaminergic neuron loss^30,31^ but alterations in cortical and cortico-striatal circuits and cognitive problems such as impaired goal-directed actions have been observed^32–34^.

It is widely accepted that neuronal dysfunction drives synaptic pathologies in PD; however, given the important roles of astrocytes in synaptic connectivity, we reasoned astrocytic dysfunction caused by LRRK2 G2019S mutations can also underlie the circuit phenotypes. Here, we explored this possibility by using LRRK2 G2019S knockin (^ki/ki^) mouse model^35,36^ in conjunction with *in vivo* astrocyte-specific genetic, proteomic, electrophysiological, and biochemical approaches.

## RESULTS

### LRRK2 G2019S mutation causes ERM hyperphosphorylation in the prefrontal cortices of mice and humans.

Cortical astrocytes play critical roles in cognitive functions by controlling the number and activity of synapses. Their highly elaborate morphology allows them to physically and functionally interact with thousands of synapses through their PAPs^37,38^. In disease states, astrocytes change their morphology and tiling, which is proposed to induce synaptic abnormalities^39^.

**Figure 1:**
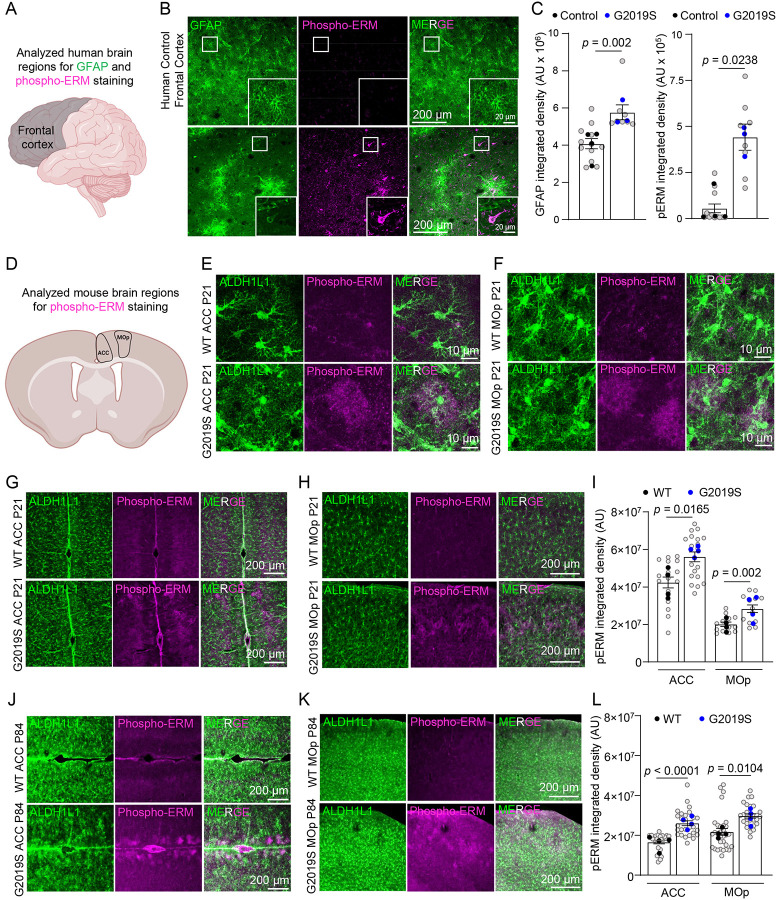
ERM phosphorylation is impaired in PD patients carrying LRRK2 G2019S mutation. (A) Schematic representation of analyzed human brain regions for phospho-ERM staining, frontal cortex. (B) Representative confocal images of GFAP (green) and ERM phosphorylation (purple) in the frontal cortex of human control subjects or human PD patients carrying LRRK2 G2019S mutation carriers at age >80 years old. Scale bar, 200 μm. (C) Quantification of GFAP integrated density in (B), n = 4 (Human control, 3 males and 1 female), 3 (LRRK2 G2019S mutation carriers, 2 males and 1 female) subjects, nested t-test, unpaired Two-tailed t-test. t (18) = 3.612, *p* = 0.0020. Quantification of phospho-ERM integrated density in (B), n = 4 (Human control, 3 males and 1 female), 3 (LRRK2 G2019S mutation carriers, 2 males and 1 female) subjects, nested t-test, unpaired Two-tailed t-test. t (4) = 3.551, *p* = 0.0238. Grey dots are the data acquired from each image. Black dots are the averaged data acquired from each control subject. Blue dots are the averaged data acquired from each LRRK2 G2019S mutation carrier. (D) Schematic representation of analyzed mouse brain regions for phospho-ERM staining, the anterior cingulate cortex (ACC), and the primary motor cortex (MOp). (E-F) Representative confocal images of phospho-ERM (purple) in the ACC and the MOp of WT or LRRK2 G2019S^ki/ki^ Aldh1L1-eGFP mice at P21. Scale bar, 10 μm. (G-H) Representative confocal images of phospho-ERM (purple) in the ACC and MOp of WT or LRRK2 G2019S^ki/ki^ Aldh1L1-eGFP mice at P21. Scale bar, 200 μm. (I) Quantification of phospho-ERM integrated density in (G-H), n = 4 (WT, 2 males and 2 females), 4 (LRRK2 G2019S^ki/ki^, 2 males and 2 females) mice, For phospho-ERM quantification in the ACC, nested t-test, unpaired two-tailed t-test. t (6) = 3.295, *p* = 0.0165. For phospho-ERM quantification in the MOp, nested t-test, unpaired two-tailed t-test. t (24) = 3.462, *p* = 0.002. (J-K) Representative confocal images of phospho-ERM (purple) in the ACC and MOp of WT or LRRK2 G2019S^ki/ki^ Aldh1L1-eGFP mice at P84. Scale bar, 200 μm. n = 4 (WT, 2 males and 2 females), 4 (LRRK2 G2019S^ki/ki^, 2 males and 2 females) mice. (L) Quantification of phospho-ERM integrated density in (J-K), n = 4 (WT, 2 males and 2 females), 4 (LRRK2 G2019S^ki/ki^, 2 males and 2 females) mice, For phospho-ERM quantification in the ACC, nested t-test, unpaired Two-tailed t-test. t (46) = 6.173, *p* < 0.0001. For phospho-ERM quantification in the MOp, nested t-test, unpaired Two-tailed t-test. t (10) = 3.146, *p* = 0.0104. Grey dots are the data acquired from each image. Black dots are the averaged data acquired from each WT mouse. Blue dots are the averaged data acquired from each LRRK2 G2019S^ki/ki^ mouse.

To study how the LRRK2 G2019S mutation impacts astrocytes in the prefrontal cortex, a cortical region linked to PD, we stained sections from the frontal cortices of age and sex-matched control and LRRK2 G2019S mutation-carrying PD patients (Supplemental Table 1) with glial fibrillary acidic protein (GFAP, a marker for astrocyte branches) and phosphorylated ERM (Phospho-ERM, which are enriched in PAPs) (Figure 1A). Remarkably, the astrocytic organization, determined by GFAP staining, was severely disrupted, and GFAP levels were significantly elevated (~42%) in PD patients carrying the LRRK2 G2019S mutation, indicative of reactive gliosis (Figure 1B–C). Unexpectedly, the phospho-ERM signal was increased by 7-fold in the frontal cortices of PD patients carrying the LRRK2 G2019S mutation (Figure 1B–C). The elevated phospho-ERM signal was observed in GFAP-positive astrocytes and other cortical cells. These data reveal that there are reactive astrocytes in the cortices of LRRK2 G2019S PD patients, and ERM phosphorylation is highly upregulated in the brains of these patients.

Is ERM hyperphosphorylation an early or late event in LRRK2 G2019S PD pathology? To address this question, we utilized the LRRK2 G2019S^ki/ki^ mice in which the human G2019S point mutation was introduced into exon 41 of the mouse *Lrrk2* gene^35,36^. To determine if ERM hyperphosphorylation localizes to astrocytes, we crossed these mice to an astrocyte reporter line carrying the Aldh1L1-eGFP transgene, which labels all astrocytes by GFP^40^. We examined the levels of phospho-ERM in coronal brain sections from 21-day-old (P21, juvenile) and 12-week-old (P84, adult) WT or LRRK2 G2019S^ki/ki^ mice. In LRRK2 G2019S^ki/ki^ mice, a brain region-specific and heterogeneous increase in phospho-ERM staining was observed in astrocytes across both age groups, compared to WT mice (Figure 1D–L). The increase in phospho-ERM in LRRK2 G2019S^ki/ki^ mice was localized to a subpopulation of astrocytes within layers (L)1 and L2–3 of the anterior cingulate cortex (ACC) and the primary motor cortex (MOp) (Figure 1E–L; Figure S1A–C) but was absent in other regions such as the primary sensory cortex (SSp) and the dorsal medial striatum (DMS) (Figure S1D–I). In P21 LRRK2 G2019S^ki/ki^ mice, the phospho-ERM integrated densities in the ACC and MOp L1 and L2–3 were increased by ~32% (ACC) and ~41% (MOp) compared to the WTs (Figure 1I), whereas in P84 LRRK2 G2019S^ki/ki^ mice, the phospho-ERM integrated densities were increased by ~57% (ACC) and ~37% (MOp) compared to the WTs (Figure 1L). Importantly, we did not observe any difference in total ERM levels in the ACC and MOp between WT and LRRK2 G2019S^ki/ki^ brain sections (Figure S1J–L). These findings show that the LRRK2 G2019S mutation enhanced ERM phosphorylation in cortical astrocytes *in vivo*. This increase in ERM phosphorylation is not uniform but rather localized to a subset of ACC and MOp astrocytes.

These findings reveal that the LRRK2 G2019S mutation increases phospho-ERM levels in the cortices of PD patients and mice. In the human samples, phospho-ERM elevation was observed concurrently with reactive gliosis (elevated GFAP staining). To determine if, in LRRK2 G2019S^ki/ki^ mice, there were reactive astrocytes detectable at early ages, we stained sections from the same P21 mouse brains with an anti-GFAP antibody. In the mouse cortex, grey matter astrocytes do not display GFAP staining unless there is reactive gliosis^41,42^. We did not detect any increase in the levels of GFAP (Figure S2A–C), indicating that phospho-ERM upregulation does not occur concurrently with reactive astrogliosis in LRRK2 G2019S^ki/ki^ mice. Distinct from the P21 and P84 LRRK2 G2019S^ki/ki^ mice where phospho-ERM was restricted to a subset of astrocytes in a brain region-specific manner, in the human patient brains (~80 years old), we saw phospho-ERM accumulation outside the GFAP-labelled astrocytes. This observation suggests aberrant ERM hyperphosphorylation in multiple cell types occurs at later stages of PD in patients with LRRK2 G2019S mutation.

### LRRK2 G2019S mutation alters excitatory and inhibitory synapse numbers and function in the cortex.

**Figure 2:**
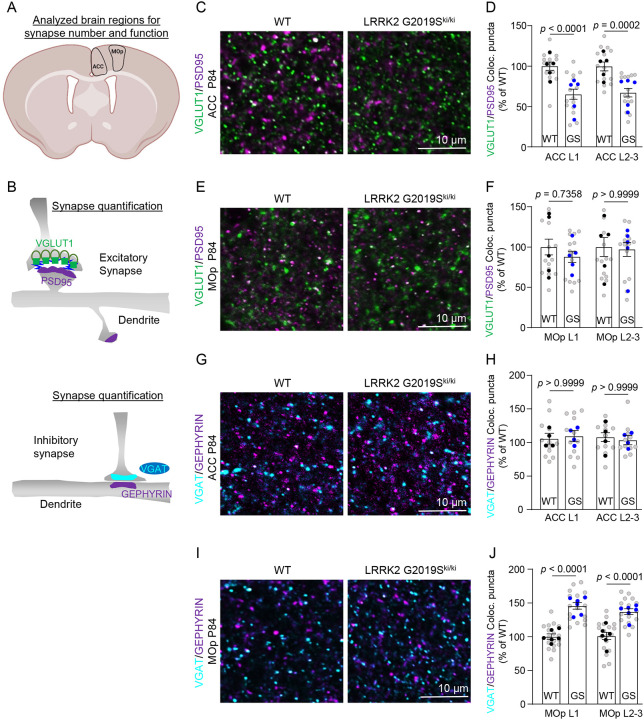
LRRK2 G2019S affects excitatory and inhibitory synapse numbers in the ACC and MOp. (A) Schematic representation of analyzed brain regions (ACC and MOp) for excitatory and inhibitory synapse numbers. (B) Schematic representations of methodologies used to quantify the VGluT1-PSD95 and VGAT-GEPHYRIN colocalized puncta. (C) Representative images from the ventral ACC of WT and LRRK2 G2019S^ki/ki^ mice that were stained with VGluT1 and PSD95 antibodies at P84. Scale bar, 10 μm. (D) Quantification of VGluT1-PSD95 co-localized puncta, normalized using the means of WT values in the ventral ACC L1 and L2–3. n = 5 (WT, 3 males and 2 females), 5 (LRRK2 G2019S^ki/ki^, 3 males and 2 females) mice. Nested One-way ANOVA [F (3, 56) = 12.48, *p* < 0.0001], Bonferroni’s multiple comparisons test revealed a significant difference between WT ACC L1 and LRRK2 G2019S^ki/ki^ ACC L1 (*p* < 0.0001, 95% C.I. = [16.83, 52.83]), and between WT ACC L2–3 and LRRK2 G2019S^ki/ki^ ACC L2–3 (*p* = 0.0002, 95% C.I. = [14.73, 50.74]). alpha = 0.05. (E) Representative images from the MOp of WT and LRRK2 G2019S^ki/ki^ mice that were stained with VGluT1 and PSD95 antibodies at P84. Scale bar, 10 μm. (F) Quantification of VGluT1-PSD95 co-localized puncta, normalized using the means of WT values in the MOp L1 and L2–3. n = 5 (WT, 3 males and 2 females), 5 (LRRK2 G2019S^ki/ki^, 3 males and 2 females) mice. Nested One-way ANOVA [F (3, 56) = 0.3681, *p* = 0.7763], Bonferroni’s multiple comparisons test revealed a significant difference between WT MOp L1 and LRRK2 G2019S^ki/ki^ MOp L1 (*p* = 0.7358, 95% C.I. = [−18.62, 42.83]), and between WT MOp L2–3 and LRRK2 G2019S^ki/ki^ MOp L2–3 (*p* > 0.9999, 95% C.I. = [−27.51, 33.94]). alpha = 0.05. (G) Representative images from the ventral ACC of WT and LRRK2 G2019S^ki/ki^ mice that were stained with VGAT and GEPHYRIN antibodies at P84. Scale bar, 10 μm. (H) Quantification of VGAT-GEPHYRIN co-localized puncta, normalized using the means of WT values in the ventral ACC L1 and L2–3. n = 4 (WT, 2 males and 2 females), 4 (LRRK2 G2019S^ki/ki^, 2 males and 2 females) mice. Nested One-way ANOVA [F (3, 43) = 0.1155, *p* = 0.9505], Bonferroni’s multiple comparisons test revealed a significant difference between WT ACC L1 and LRRK2 G2019S^ki/ki^ ACC L1 (*p* > 0.9999, 95% C.I. = [−28.54, 20.66]), and between WT ACC L2–3 and LRRK2 G2019S^ki/ki^ ACC L2–3 (*p* > 0.9999, 95% C.I. = [−20.71, 29.60]). alpha = 0.05. (I) Representative images from MOp of WT and LRRK2 G2019S^ki/ki^ mice that were stained with VGAT and GEPHYRIN antibodies at P84. Scale bar, 10 μm. (J) Quantification of VGAT-GEPHYRIN co-localized puncta, normalized using the means of WT values in MOp L1 and L2–3. n = 6 (WT, 3 males and 3 females), 6 (LRRK2 G2019S^ki/ki^, 3 males and 3 females) mice. Nested One-way ANOVA [F (3, 68) = 25.88, *p* < 0.0001], Bonferroni’s multiple comparisons test revealed a significant difference between WT MOp L1 and LRRK2 G2019S^ki/ki^ MOp L1 (*p* < 0.0001, 95% C.I. = [−60.89, −30.43]), and between WT MOp L2–3 and LRRK2 G2019S^ki/ki^ MOp L2–3 (*p* < 0.0001, 95% C.I. = [−51.48, −21.02]). alpha = 0.05. Grey dots are the data acquired from each synapse image. Black dots are the averaged data acquired from each WT mouse. Blue dots are the averaged data acquired from each LRRK2 G2019S^ki/ki^ mouse.

Previous studies did not observe dopaminergic neuron loss or motor dysfunction in the LRRK2 G2019S^ki/ki^ mice^31,43^; however, synapse dysfunction at cortico-striatal circuits was reported in adult mice^34^. Despite the critical role of Lrrk2 in cortico-striatal synapses and PD-related cortical circuits^44^, whether LRRK2 G2019S affects cortical synapse numbers and function has not been explored. Understanding these cortical synaptic abnormalities is crucial, as they may underlie the early cognitive deficits in PD that are unrelated to dopaminergic dysfunction. Therefore, we next investigated how LRRK2 G2019S mutation impacts synaptic connectivity in the ACC and MOp L1 and L2–3 (Figure 2A), regions in which ERM hyperphosphorylation within astrocytes was observed.

Astrocytes, through secreted factors and direct contact with synapses, strongly promote excitatory and inhibitory synapse formation and maintenance^13^. We quantified the density of structural excitatory and inhibitory intracortical synapses in WT and LRRK2 G2019S^ki/ki^ mice. To do so, we marked the excitatory synapses as the co-localization of the presynaptic Vesicular Glutamate Transporter 1 (VGlut1) and the Postsynaptic Density Protein (PSD95), whereas inhibitory synapses were labeled using presynaptic Vesicular GABA Transporter (VGAT), and postsynaptic gephyrin (GEPHYRIN)^45,46^ (Figure 2B). Structural synapses were identified as the apposition and co-localization of presynaptic and postsynaptic markers, which are in two distinct neuronal compartments (i.e., axons and dendrites) and would only appear to co-localize at synapses due to their proximity^47^.

Excitatory synapse densities were quantified both at P21 and P84. In the ACC of the LRRK2 G2019S^ki/ki^ mice, there was a significant decrease in intracortical excitatory synapse densities in both layers and ages we analyzed (~30%) when compared to the WT controls (Figure 2C–D and Figure S3A–B). However, no differences in excitatory synapse numbers were observed between genotypes in the MOp (Figure 2E–F and Figure S3C–D). When we quantified the densities of inhibitory synapses in the MOp, we found significant differences between genotypes (~40% increase). In contrast, no differences were observed for inhibitory synapse numbers in the ACC of WT and LRRK2 G2019S^ki/ki^ mice (Figure 2G–J). These results revealed that the LRRK2 G2019S mutation causes a brain region-specific reduction in excitatory synapse densities in the ACC and an increase in inhibitory synapse densities in the MOp, highlighting brain region-specific synaptic alterations associated with this mutation.

**Figure 3:**
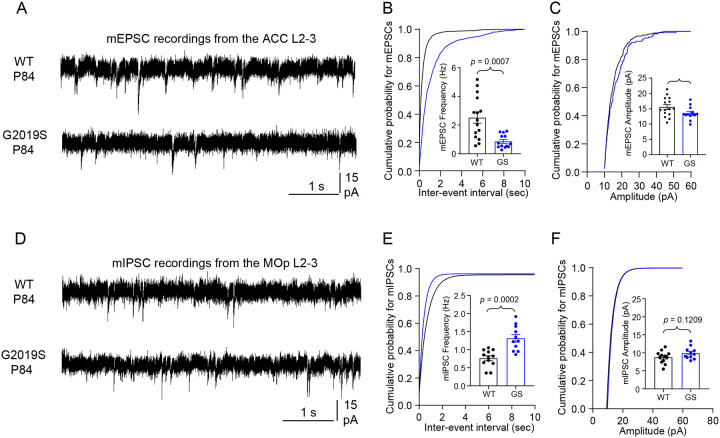
LRRK2 G2019S affects excitatory and inhibitory synapse function in the ACC and MOp. (A) Representative mEPSC traces from the ventral ACC L2–3 pyramidal neurons in acute brain slices of WT and LRRK2 G2019S^ki/ki^ mice. (B) Cumulative probability plots and quantification of synaptic event frequency, n = 15 (WT), 13 (LRRK2 G2019S^ki/ki^) neurons from 4 mice per genotype. Kolmogorov-Smirnov test (D = 0.504, *p* < 0.001). The average frequency of mEPSC in WT (2.521 ± 0.3846) and LRRK2 G2019S^ki/ki^ (0.8691 ± 0.1309) mice. Unpaired Two-tailed t-test [t (26) = 3.828, *p* = 0.0007]. (C) Cumulative probability plots and quantification of synaptic event amplitude, n = 14 (WT), 12 (LRRK2 G2019S^ki/ki^) neurons from 4 mice per genotype. Kolmogorov-Smirnov test (D = 0.194, *p* < 0.0001). The average amplitude of mEPSC in WT (7.826 ± 0.8259) and LRRK2 G2019S^ki/ki^ (6.959 ± 0.9015) mice. Unpaired t-test [t (24) = 0.7093, *p* = 0.485]. (D) Representative mIPSC traces from MOp L2–3 pyramidal neurons in acute brain slices of WT and LRRK2 G2019S^ki/ki^ mice. (E) Cumulative probability plots and quantification of synaptic event frequency, n = 12 (WT), 11 (LRRK2 G2019S^ki/ki^) neurons from 4 mice per genotype. Kolmogorov-Smirnov test (D = 0.424, *p* < 0.0001). The average frequency of mIPSC in WT (0.7606 ± 0.06816) and LRRK2 G2019S^ki/ki^ (1.312 ± 0.1041) mice. Unpaired Two-tailed t-test [t (21) = 4.503, *p* = 0.0002]. (F) Cumulative probability plots and quantification of synaptic event amplitude, n = 12 (WT), 11 (LRRK2 G2019S^ki/ki^) neurons from 4 mice per genotype. Kolmogorov-Smirnov test (D = 0.382, *p* < 0.0001). The average amplitude of mIPSC in WT (8.776 ± 0.4961) and LRRK2 G2019S^ki/ki^ (9.921 ± 0.5043) mice. Unpaired t-test [t (21) = 1.617, *p* = 0.1209]. Data are presented as mean ± s.e.m.

Next, we used electrophysiology to test the functional impact of altered synapse density in the ACC and the MOp of LRRK2 G2019S^ki/ki^ mice on synaptic function. We recorded miniature excitatory postsynaptic currents (mEPSCs) in the ACC L2–3 pyramidal neurons and miniature inhibitory postsynaptic currents (mIPSCs) in the MOp L2–3 pyramidal neurons from acute brain slices of P84 LRRK2 G2019S^ki/ki^ and WT mice. In agreement with a reduction in excitatory synapse density in the ACC, LRRK2 G2019S^ki/ki^ L2–3 neurons displayed mEPSC frequency reduced by ~65% and a corresponding right shift in the cumulative distributions of mEPSC inter-event intervals (Figure 3A–B) when compared to WT neurons. There were no significant changes in the mean mEPSC amplitude between genotypes (Figure 3C). mEPSC frequency changes correlate with synapse numbers and/or the probability of presynaptic glutamate release^48^, whereas mEPSC amplitude indicates the synaptic strength reflecting the number of AMPARs at the postsynapse^49^. Therefore, together with our anatomical data, these physiological analyses reveal that the LRRK2 G2019S mutation reduces the number of intracortical excitatory connections formed on L2–3 pyramidal neurons in the ACC without a change in synaptic strength. In contrast, in the MOp, we found a robust ~72% increase in mIPSC frequency when we recorded from the L2–3 pyramidal neurons of the LRRK2 G2019S^ki/ki^ mice compared to WT, with no significant change in amplitude (Figure 3D–F). The increased mIPSC frequency aligns with our findings that inhibitory synapse numbers are elevated in the MOp of the LRRK2 G2019S^ki/ki^ mice.

### Reducing ERM hyperphosphorylation in astrocytes rescues excitatory synapse density and function in the ACC of adult LRRK2 G2019S^ki/ki^ mice.

Lrrk2 phosphorylates a conserved threonine residue present in all ERM proteins^50,51^. We found that LRRK2 G2019S mutation leads to increased ERM phosphorylation in astrocytes (Figure 1). We hypothesized that dampening ERM phosphorylation, specifically in astrocytes, might be sufficient to restore the synapse alteration in LRRK2 G2019S^ki/ki^ mice. To test this hypothesis specifically in astrocytes *in vivo*, we first investigated if we could reduce ERM phosphorylation in LRRK2 G2019S^ki/ki^ astrocytes by virally overexpressing an Ezrin mutant lacking the ability to be phosphorylated. We chose Ezrin because Ezrin is highly abundant and specifically enriched in astrocytes compared to its family members, Radixin and Moesin^52^ (Figure S4A–B). Switching the amino acid threonine (T) at location 567 with alanine (A) results in a version of the protein that cannot be phosphorylated (Phospho-dead Ezrin, Figure S4C)^53,54^.

To determine if phospho-dead Ezrin can reduce ERM phosphorylation in astrocytes of LRRK2 G2019S^ki/ki^ mice, we retro-orbitally injected 9-week-old WT and LRRK2 G2019S^ki/ki^ mice with adenoviruses (AAVs) to express Hemagglutinin (HA)-tagged WT Ezrin or phospho-dead Ezrin under the control of the astrocyte-specific GfaABC1D promoter (Figure S4D). The injected mouse brains were harvested at 12 weeks (3 weeks after injection) for histological analyses.

**Figure 4:**
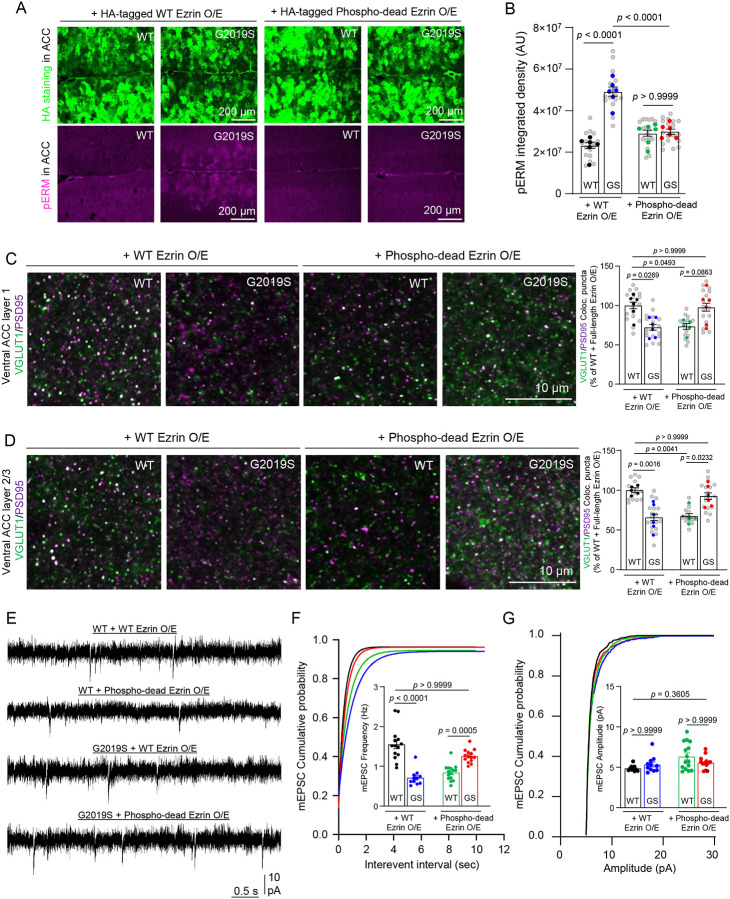
Overexpression of phospho-dead Ezrin in adult LRRK2 G2019S^ki/ki^ astrocytes restores excitatory synapse number and function in the ventral ACC. (A) Representative images from the ventral ACC of WT and LRRK2 G2019S^ki/ki^ mice injected with AAV-HA-tagged-WT Ezrin or AAV-HA-tagged-Phospho-dead Ezrin that were stained with HA and phospho-ERM antibodies at P84. Scale bar, 200 μm. n = 6 (WT + WT Ezrin O/E, 3 males and 3 females), 5 (WT + Phospho-dead Ezrin O/E, 3 males and 2 females), 6 (LRRK2 G2019S^ki/ki^ + WT Ezrin O/E, 3 males and 3 females), 6 (LRRK2 G2019S^ki/ki^ + Phospho-dead Ezrin O/E, 3 males and 3 females) mice. (B) Quantification of phosphor-ERM integrated density in (A). n = 6 (WT + WT Ezrin O/E, 3 males and 3 females), 6 (WT + Phospho-dead Ezrin O/E, 3 males and 3 females), 6 (LRRK2 G2019S^ki/ki^ + WT Ezrin O/E, 3 males and 3 females), 6 (LRRK2 G2019S^ki/ki^ + Phospho-dead Ezrin O/E, 3 males and 3 females) mice. One-way ANOVA [F (3, 20) = 31.62, *p* < 0.0001], Bonferroni’s multiple comparisons test revealed a significant difference between WT + WT Ezrin O/E and LRRK2 G2019S^ki/ki^ + WT Ezrin O/E (*p* < 0.0001, 95% C.I. = [−34272667, −17645403]), and between LRRK2 G2019S^ki/ki^ + WT Ezrin O/E and LRRK2 G2019S^ki/ki^ + Phospho-dead Ezrin O/E (*p* < 0.0001, 95% C.I. = [11069274, 27696537]). There were no differences between WT + WT Ezrin O/E and WT + Phospho-dead Ezrin O/E (*p* = 0.3065, 95% C.I. = [−14207483, 2419780]), and between WT + Phospho-dead Ezrin O/E and LRRK2 G2019S^ki/ki^ + Phospho-dead Ezrin O/E (*p* > 0.9999, 95% C.I. = [−8995910, 7631353]), alpha = 0.05. (C) Representative images from the ventral ACC L1 of WT and LRRK2 G2019S^ki/ki^ mice injected with AAV-HA-tagged-WT Ezrin or AAV-HA-tagged-Phospho-dead Ezrin that were stained with VGluT1 and PSD95 antibodies at P84. Scale bar, 10 μm. Quantification of VGluT1-PSD95 co-localized puncta, normalized using the means of WT mice injected with AAV-HA-tagged-WT Ezrin in the ventral ACC L1. n = 6 (WT + WT Ezrin O/E, 3 males and 3 females), 5 (WT + Phospho-dead Ezrin O/E, 3 males and 2 females), 6 (LRRK2 G2019S^ki/ki^ + WT Ezrin O/E, 3 males and 3 females), 6 (LRRK2 G2019S^ki/ki^ + Phospho-dead Ezrin O/E, 3 males and 3 females) mice. One-way ANOVA [F (3, 19) = 5.882, *p* = 0.0051], Bonferroni’s multiple comparisons test revealed a significant difference between WT + WT Ezrin O/E and WT + Phospho-dead Ezrin O/E (*p* = 0.0493, 95% C.I. = [0.05586, 53.21]), between WT + WT Ezrin O/E and LRRK2 G2019S^ki/ki^ + WT Ezrin O/E (*p* = 0.0269, 95% C.I. = [2.396, 53.08]), and between LRRK2 G2019S^ki/ki^ + WT Ezrin O/E and LRRK2 G2019S^ki/ki^ + Phospho-dead Ezrin O/E (*p* = 0.049, 95% C.I. = [−50.77, −0.08163]). There were no differences between WT + WT Ezrin O/E and LRRK2 G2019S^ki/ki^ + Phospho-dead Ezrin O/E (*p* > 0.9999, 95% C.I. = [−23.03, 27.66]), between WT + Phospho-dead Ezrin O/E and LRRK2 G2019S^ki/ki^ + WT Ezrin O/E (*p* > 0.9999, 95% C.I. = [−25.48, 27.68]), and between WT + Phospho-dead Ezrin O/E and LRRK2 G2019S^ki/ki^ + Phospho-dead Ezrin O/E (*p* = 0.0863, 95% C.I. = [−50.90, 2.259]), alpha = 0.05. (D) Representative images from the ventral ACC L2–3 of WT and LRRK2 G2019S^ki/ki^ mice injected with AAV-HA-tagged-WT Ezrin or AAV-HA-tagged-Phospho-dead Ezrin that were stained with VGluT1 and PSD95 antibodies at P84. Scale bar, 10 μm. Quantification of VGluT1-PSD95 co-localized puncta, normalized using the means of WT mice injected with AAV-HA-tagged-WT Ezrin in the ventral ACC L2–3. n = 6 (WT + WT Ezrin O/E, 3 males and 3 females), 5 (WT + Phospho-dead Ezrin O/E, 3 males and 2 females), 6 (LRRK2 G2019S^ki/ki^ + WT Ezrin O/E, 3 males and 3 females), 6 (LRRK2 G2019S^ki/ki^ + Phospho-dead Ezrin O/E, 3 males and 3 females) mice. One-way ANOVA [F (3, 18) = 10.45, *p* = 0.0003], Bonferroni’s multiple comparisons test revealed a significant difference between WT + WT Ezrin O/E and WT + Phospho-dead Ezrin O/E (*p* = 0.0041, 95% C.I. = [8.857, 55.39]), between WT + WT Ezrin O/E and LRRK2 G2019S^ki/ki^ + WT Ezrin O/E (*p* = 0.0016, 95% C.I. = [11.67, 56.22]), between WT + Phospho-dead Ezrin O/E and LRRK2 G2019S^ki/ki^ + Phospho-dead Ezrin O/E (*p* = 0.0232, 95% C.I. = [−47.19.67, −2.636]) and between LRRK2 G2019S^ki/ki^ + WT Ezrin O/E and LRRK2 G2019S^ki/ki^ + Phospho-dead Ezrin O/E (*p* = 0.0092, 95% C.I. = [−47.97.77, −5.494]). There were no differences between WT + WT Ezrin O/E and LRRK2 G2019S^ki/ki^ + Phospho-dead Ezrin O/E (*p* > 0.9999, 95% C.I. = [−15.06, 29.49]), and between WT + Phospho-dead Ezrin O/E and LRRK2 G2019S^ki/ki^ + WT Ezrin O/E (*p* > 0.9999, 95% C.I. = [−20.45, 24.10]), alpha = 0.05. (E) Representative mEPSC traces from the ventral ACC L2–3 pyramidal neurons in acute brain slices of WT and LRRK2 G2019S^ki/ki^ mice injected with AAV-HA-tagged-WT Ezrin or AAV-HA-tagged-Phospho-dead Ezrin. (F) Cumulative probability plots and quantification of synaptic event frequency. n = 14 (WT + WT Ezrin O/E), 15 (WT + Phospho-dead Ezrin O/E), 11 (LRRK2 G2019S^ki/ki^ + WT Ezrin O/E), 14 (LRRK2 G2019S^ki/ki^ + Phospho-dead Ezrin O/E) neurons from 4 mice per condition. Kruskal-Wallis test [H (3) = 36.83, *p* < 0.0001]. Average frequency of mEPSC in WT + WT Ezrin O/E (1.555 ± 0.1125), WT + Phospho-dead Ezrin O/E (0.8416 ± 0.05145), LRRK2 G2019S^ki/ki^ + WT Ezrin O/E (0.7154 ± 0.05959), and LRRK2 G2019S^ki/ki^ + Phospho-dead Ezrin O/E (1.253 ± 0.04424) mice. The posthoc Dunn’s test with Bonferroni adjustments showed to be significant for WT + WT Ezrin O/E vs. WT + Phospho-dead Ezrin O/E (*p* < 0.0001), WT + WT Ezrin O/E vs. LRRK2 G2019S^ki/ki^ + WT Ezrin O/E (*p* < 0.0001), WT + Phospho-dead Ezrin O/E vs. LRRK2 G2019S^ki/ki^ + Phospho-dead Ezrin O/E (*p* = 0.0084), and LRRK2 G2019S^ki/ki^ + WT Ezrin O/E vs. LRRK2 G2019S^ki/ki^ + Phospho-dead Ezrin O/E (*p* = 0.0005). The posthoc Dunn’s test with Bonferroni adjustments revealed no significant difference between WT + WT Ezrin O/E and LRRK2 G2019S^ki/ki^ + Phospho-dead Ezrin O/E (*p* > 0.9999) and between WT + Phospho-dead Ezrin O/E and LRRK2 G2019S^ki/ki^ + WT Ezrin O/E (*p* > 0.9999). (G) Cumulative probability plots and quantification of synaptic event amplitude. n = 11 (WT + WT Ezrin O/E), 15 (WT + Phospho-dead Ezrin O/E), 11 (LRRK2 G2019S^ki/ki^ + WT Ezrin O/E), 13 (LRRK2 G2019S^ki/ki^ + Phospho-dead Ezrin O/E) neurons from 4 mice per condition. Kruskal-Wallis test [H (3) = 7.051, *p* = 0.0703]. Average amplitude of mEPSC in WT + WT Ezrin O/E (4.873 ± 0.1034), WT + Phospho-dead Ezrin O/E (6.339 ± 0.4284), LRRK2 G2019S^ki/ki^ + WT Ezrin O/E (5.354 ± 0.2988), and LRRK2 G2019S^ki/ki^ + Phospho-dead Ezrin O/E (5.591 ± 0.2247) mice. The posthoc Dunn’s test with Bonferroni adjustments revealed no significant difference for WT + WT Ezrin O/E vs. WT + Phospho-dead Ezrin O/E (*p* = 0.0688), WT + WT Ezrin O/E vs. LRRK2 G2019S^ki/ki^ + WT Ezrin O/E (*p* > 0.9999), WT + Phospho-dead Ezrin O/E vs. LRRK2 G2019S^ki/ki^ + Phospho-dead Ezrin O/E (*p* > 0.9999), and LRRK2 G2019S^ki/ki^ + WT Ezrin O/E vs. LRRK2 G2019S^ki/ki^ + Phospho-dead Ezrin O/E (*p* > 0.9999), WT + WT Ezrin O/E vs. LRRK2 G2019S^ki/ki^ + Phospho-dead Ezrin O/E (*p* = 0.3605), and WT + Phospho-dead Ezrin O/E vs. LRRK2 G2019S^ki/ki^ + WT Ezrin O/E (*p* = 0.9468). alpha = 0.05. Data are presented as mean ± s.e.m.

The widespread viral expression of Ezrin in astrocytes was verified by staining with an anti-HA tag antibody (Figure 4A). First, overexpressing WT or phospho-dead Ezrin in WT mouse brains did not increase the phospho-ERM level in the ACC and MOp (Figure 4A–B). However, WT Ezrin overexpression in the LRRK2 G2019S^ki/ki^ mouse brains was accompanied by a robust phospho-ERM elevation in a population of astrocytes in the ACC and MOp (Figure 4A–B). In contrast, overexpression of the phospho-dead Ezrin eliminated the ERM hyperphosphorylation in the LRRK2 G2019S^ki/ki^ ACC and MOp (Figure 4A–B). This observation indicates that the LRRK2 G2019S mutation sensitizes astrocytes to Lrrk2-mediated ERM phosphorylation.

To determine if overexpressing phospho-dead Ezrin can also dampen hyperphosphorylation of another Lrrk2 substrate, Rab10^27,55^, We stained sections from the same brains as in Figure 4A–B with antibodies against phosphorylated Rab10 (Thr 73). Rab10 is a member of the Rab family of small GTPases that plays crucial roles in neuronal function and intracellular transport pathways^56^. As expected, we found higher Rab10 phosphorylation in LRRK2 G2019S^ki/ki^ brain sections than in WT mice. However, neither WT nor phospho-dead Ezrin overexpression impacted Rab10 phosphorylation (Figure S5A–B). These results show that phospho-dead Ezrin overexpression in astrocytes specifically reduces ERM hyperphosphorylation without impacting another Lrrk2 substrate, Rab10.

Given the effects of phospho-dead Ezrin in dampening ERM hyperphosphorylation, we next tested whether this manipulation in adult LRRK2 G2019S^ki/ki^ astrocytes can reverse the synaptic phenotypes we observed in the ACC and MOp. To do so, we followed the same experimental strategy and timeline as in Figure S4D. WT Ezrin overexpression in astrocytes did not affect the excitatory synapse density decline in the ACC L1 and L2–3 of LRRK2 G2019S^ki/ki^ mice. In contrast, overexpression of phospho-dead Ezrin reduced excitatory synapse density in the WT background by ~30% (Figure 4C–D). Conversely, astrocytic overexpression of phospho-dead Ezrin in LRRK2 G2019S^ki/ki^ rescued the excitatory synapse numbers to WT levels in the ACC (Figure 4C–D). These data suggest that the reduction in synapse numbers we observed in the ACC of the LRRK2 G2019S^ki/ki^ mice is largely due to an astrocytic dysfunction through dysregulated ERM phosphorylation. These results also indicate that astrocytes contribute to excitatory synaptic dysfunction in the LRRK2 G2019S^ki/ki^ mouse cortex.

We next investigated whether the same manipulation of adult astrocytes can change the inhibitory synapse number in the MOp. We found that neither the WT nor the phospho-dead Ezrin overexpression in adult astrocytes can rescue the density of inhibitory synapses back to WT levels in the MOp L1 and L2–3 of LRRK2 G2019S^ki/ki^ mice (Figure S5C–D). In summary, phospho-dead Ezrin expression in adult WT or LRRK2 G2019S^ki/ki^ astrocytes is sufficient to modify excitatory synapse numbers in the ACC but cannot restore or impair inhibitory synaptic connectivity in the MOp. It is plausible that LRRK2 G2019S mutation impairs inhibitory synapses in the MOp by altering the number or connectivity of cortical interneurons through a developmental mechanism. Thus, manipulation in adults is insufficient to eliminate this inhibitory synapse phenotype.

Because astrocytic overexpression of phospho-dead Ezrin impacted excitatory synapse numbers in the ACC of WT and LRRK2 G2019S^ki/ki^ mice, we next performed whole-cell patch-clamp recordings from ACC L2–3 pyramidal neurons transduced with the same AAVs. Overexpression of WT Ezrin in LRRK2 G2019S^ki/ki^ astrocytes did not rescue the decreased frequency of mEPSCs in ACC L2–3 neurons. However, overexpression of phospho-dead Ezrin in LRRK2 G2019S^ki/ki^ astrocytes restored the frequency up to the levels of WT mice expressing WT Ezrin (Figure 4E–F). Interestingly, overexpression of phospho-dead Ezrin in WT astrocytes significantly reduced the frequency of mEPSCs (~54%) in WT ACC neurons (Figure 4E–F). Posthoc comparisons confirmed no significant differences in mean mEPSC amplitudes across all 4 conditions (Figure 4G).

Altogether, these electrophysiological and neuroanatomical analyses show that the LRRK2 G2019S mutation reduces the density and function of excitatory synaptic inputs made onto the ACC L2–3 but increases the density and function of inhibitory synaptic inputs made onto the MOp L2–3. These data reveal that the LRRK2 G2019S mutation dysregulates proper excitatory and inhibitory synapse connectivity and function in the cortex in a brain region-specific manner. Because astrocytes control the number and function of cortical synapses, these results also pinpoint a link between LRRK2 G2019S-mediated ERM hyperphosphorylation in astrocytes (Figure 1) and the synaptic dysfunctions in the ACC and MOp (Figure 2 and 3). Our results also reveal that astrocytic overexpression of phospho-dead Ezrin in adult LRRK2 G2019S^ki/ki^ mice can recover excitatory synapse density and function, whereas the same manipulation disrupts synapse numbers and function in WT ACC neurons (Figure 4). These findings show that balancing ERM phosphorylation in astrocytes is crucial for maintaining excitatory synapse density and function in the ACC.

### Lrrk2 regulates astrocyte morphological complexity *in vivo* through ERM phosphorylation.

Astrocytes gain their elaborate morphology during the second and third postnatal weeks in mice^57,58^. Previous studies highlighted the importance of astrocyte morphology in the regulation of synapse numbers and function^13^. ERM proteins are structural components of perisynaptic astrocyte processes^59^. When phosphorylated and bound to phosphatidylinositol 4,5-bisphosphate (PIP2), ERM proteins change conformation and link the F-actin cytoskeleton to the cell membrane^54^. This switch is thought to be important in organizing cell morphology by allowing process outgrowth^53,54^. Ezrin is the most abundant ERM protein in astrocytes and is required for proper astrocyte morphogenesis^60–62^. Based on our observation that ERM hyperphosphorylation in LRRK2 G2019S^ki/ki^ astrocytes causes aberrant cortical synaptic connectivity as early as P21, we next investigated how this mutation impacts astrocyte morphological complexity.

**Figure 5:**
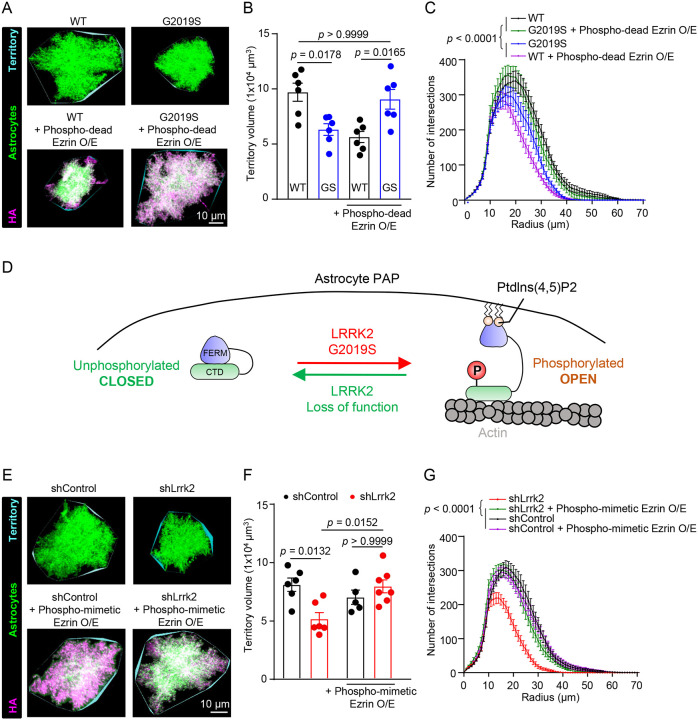
Astrocytic LRRK2 controls astrocyte morphology by balancing ERM phosphorylation levels *in vivo*. (A) Images of ACC and MOp L2–3 WT and LRRK2 G2019S^ki/ki^ astrocytes at P21 expressing PB-mCherry-CAAX ± Phospho-dead Ezrin. Astrocyte territory in cyan. Scale bar, 10 μm. (B) Average territory volumes of P21 astrocytes. n = 16 (WT; 6 mice), 18 (WT + Phospho-dead Ezrin O/E; 6 mice), 16 (LRRK2 G2019S^ki/ki^; 6 mice), 17 (LRRK2 G2019S^ki/ki^ + Phospho-dead Ezrin O/E; 6 mice). Statistical significance was determined by One-way ANOVA [F (3, 20) = 7.987, *p* = 0.0011], Bonferroni multiple comparisons revealed a significant difference between WT and LRRK2 G2019S^ki/ki^ (*p* = 0.0178, 95% C.I. = [0.4542, 6.313]), between WT and WT + Phospho-dead Ezrin O/E (*p* = 0.0037, 95% C.I. = [1.132, 6.991]), and between WT + Phospho-dead Ezrin O/E and LRRK2 G2019S^ki/ki^ + Phospho-mimetic Ezrin O/E (*p* = 0.0165, 95% C.I. = [−6.346, −0.4875]), and no differences between WT and LRRK2 G2019S^ki/ki^ + Phospho-mimetic Ezrin O/E (*p* > 0.9999, 95% C.I. = [−2.284, 3.574]), between LRRK2 G2019S^ki/ki^ and WT + Phospho-dead Ezrin O/E (*p* > 0.9999, 95% C.I. = [−2.251, 3.608]), and between LRRK2 G2019S^ki/ki^ and LRRK2 G2019S^ki/ki^ + Phospho-mimetic Ezrin O/E (*p* = 0.0763, 95% C.I. = [−5.668, 0.1908]), alpha = 0.05. Data points are mouse averages. (C) Quantification of astrocyte branching complexity. n = 16 (WT; 6 mice), 18 (WT + Phospho-dead Ezrin O/E; 6 mice), 16 (LRRK2 G2019S^ki/ki^; 6 mice), 17 (LRRK2 G2019S^ki/ki^ + Phospho-dead Ezrin O/E; 6 mice). Two-way ANOVA for repeated measures. Main effects of conditions [F (2.505, 3305) = 158.4, *p* < 0.0001] and radius [F (88, 1602) = 218.5, *p* < 0.0001] and interaction [F (264, 3958) = 10.62, *p* < 0.0001]. Bonferroni multiple comparisons revealed a significant difference between WT and LRRK2 G2019S^ki/ki^ (*p* < 0.0001, 95% C.I. = [16.09, 26.40]), between WT and WT + Phospho-dead Ezrin O/E (*p* < 0.0001, 95% C.I. = [29.06, 39.54]), between LRRK2 G2019S^ki/ki^ and WT + Phospho-dead Ezrin O/E (*p* < 0.0001, 95% C.I. = [7.6, 18.52]), between LRRK2 G2019S^ki/ki^ and LRRK2 G2019S^ki/ki^ + Phospho-dead Ezrin O/E (*p* < 0.0001, 95% C.I. = [−22.19, −13.43]), and between WT + Phospho-dead Ezrin O/E and LRRK2 G2019S^ki/ki^ + Phospho-dead Ezrin O/E (*p* < 0.0001, 95% C.I. = [−37.02, −24.72]), and no difference between WT and LRRK2 G2019S^ki/ki^ + Phospho-dead Ezrin O/E (*p* = 0.5469, 95% C.I. = [−1.932, 8.796]). (D) A proposed working model of how Ezrin in astrocytes transitions between open and closed conformation and Lrrk2 regulates astrocyte morphogenesis by modulating this transition. (E) Images of ACC and MOp L2–3 astrocytes at P21 expressing shControl-mCherry-CAAX or sh*Lrrk2*-mCherry-CAAX ± Phospho-mimetic Ezrin. Astrocyte territory in cyan. Scale bar, 10 μm. (F) Average territory volumes of P21 astrocytes. n = 18 (shControl; 6 mice), 18 (shControl + Phospho-mimetic Ezrin O/E; 5 mice), 22 (sh*Lrrk2*; 6 mice), 19 (sh*Lrrk2* + Phospho-mimetic Ezrin O/E; 6 mice). Statistical significance was determined by One-way ANOVA [F (3, 19) = 5.47, *p* = 0.0070], Bonferroni multiple comparisons revealed a significant difference between shControl and sh*Lrrk2* (*p* = 0.0132, 95% C.I. = [0.4928, 5.387]), and between sh*Lrrk2* and sh*Lrrk2* + Phospho-mimetic Ezrin O/E (*p* = 0.0152, 95% C.I. = [−5.337, −0.4428]), and no differences between shControl and shControl + Phospho-mimetic Ezrin O/E (*p* > 0.9999, 95% C.I. = [−1.495, 3.639]), between shControl and sh*Lrrk2* + Phospho-mimetic Ezrin O/E (*p* > 0.9999, 95% C.I. = [−2.397, 2.497]), and between shControl + Phospho-mimetic Ezrin O/E and sh*Lrrk2* + Phospho-mimetic Ezrin O/E (*p* > 0.9999, 95% C.I. = [−3.589, 1.545]), alpha = 0.05. Data points are mouse averages. (G) Quantification of astrocyte branching complexity. n = 16 (shControl), 22 (shControl + Phospho-mimetic Ezrin O/E), 21 (sh*Lrrk2*), 21 (sh*Lrrk2* + Phospho-mimetic Ezrin O/E). Two-way ANOVA for repeated measures. Main effects of conditions [F (2.435, 7008) = 225.0, *p* < 0.0001] and radius [F (88, 8633) = 325.4, *p* < 0.0001] and interaction [F (264, 8633) = 5.908, *p* < 0.0001]. Bonferroni multiple comparisons revealed a significant difference between shControl and sh*Lrrk2* (*p* < 0.0001, 95% C.I. = [31.02, 40.20]), between sh*Lrrk2* and shControl + Phospho-mimetic Ezrin O/E (*p* < 0.0001, 95% C.I. = [−38.59, −29.67]), between sh*Lrrk2* and sh*Lrrk2* + Phospho-mimetic Ezrin O/E (*p* < 0.0001, 95% C.I. = [−34.32, −25.69]), between shControl and sh*Lrrk2* + Phospho-mimetic Ezrin O/E (*p* = 0.0139, 95% C.I. = [0.7510, 10.47]), and between shControl + Phospho-mimetic Ezrin O/E and sh*Lrrk2* + Phospho-mimetic Ezrin O/E (*p* = 0.0188, 95% C.I. = [0.4426, 7.813]), and no difference between shControl and shControl + Phospho-mimetic Ezrin O/E (*p* > 0.9999, 95% C.I. = [−3.083, 6.044]). Data are presented as mean ± s.e.m.

To determine if astrocyte morphology was altered in the LRRK2 G2019S^ki/ki^ cortices, we sparsely labeled astrocytes with a membrane-targeted fluorescent protein (mCherry-CAAX), using postnatal astrocyte labeling by electroporation (PALE) in WT and LRRK2 G2019S^ki/ki^ mice and imaged whole astrocytes with confocal microscopy at high resolution (Figure S6A). We focused our analyses of astrocyte territory size and complexity (see Methods for details) on the ACC and MOp L1 and L2–3 at P21. In LRRK2 G2019S^ki/ki^ mice, the territory volumes of astrocytes were significantly smaller (reduced by ~34%) than WT astrocytes (Figure 5A–B). In addition, compared to WT astrocytes, LRRK2 G2019S^ki/ki^ astrocytes were significantly less complex (Figure 5C). But the reduction in astrocyte size and complexity in LRRK2 G2019S^ki/ki^ did not change the number of ALDH1L1+/SOX9+ cells in the ACC and MOp (Figure S6B–D). These results reveal that LRRK2 G2019S mutation reduces astrocyte morphological complexity without affecting astrocyte numbers *in vivo*.

Because phospho-dead Ezrin overexpression in astrocytes differentially impacts synapse numbers and function in WT and LRRK2 G2019S^ki/ki^ mice (Figure 4), we hypothesized that overexpression of phospho-dead Ezrin would also impact astrocyte morphology differently between genotypes. To test this possibility, we performed PALE to introduce mCherry-CAAX and phospho-dead Ezrin into WT or LRRK2 G2019S^ki/ki^ mouse astrocytes. The overexpression of phospho-dead Ezrin decreased the territory volume and morphological complexity of WT astrocytes (Figure 5A–C). However, phospho-dead Ezrin expression restored astrocyte territory volume and complexity in LRRK2 G2019S^ki/ki^ mice (Figure 5A–C). Our results show that the abundance of unphosphorylated Ezrin differentially regulates astrocyte morphology in WT versus LRRK2 G2019S^ki/ki^ astrocytes.

Previous studies suggested phosphorylation at T567 promotes Ezrin acquiring an open conformation by binding to phosphatidylinositol-4,5-bisphosphate (PIP2)^63^. In this conformation, the N-terminus interacts with membrane proteins, and the C-terminus is available to bind to the actin cytoskeleton^64,65^. An Ezrin mutant in which the T567 residue is switched with aspartate (D) prefers Ezrin’s open conformation, thereby mimicking the phosphorylated state (Phospho-mimetic Ezrin, Figure S4C)^66,67^. Conversely, the phospho-dead Ezrin remains in the closed conformation in which the N and C terminal domains are bound together. This closed conformation is proposed to be unable to interact with membranes or actin^53,68^. Because we found that phospho-dead Ezrin differentially impacts WT and LRRK2 G2019S^ki/ki^ astrocytes, we hypothesized that Ezrin’s transition between open and closed conformation is regulated by LRRK2 function and should be tightly balanced for proper astrocyte morphogenesis (Figure 5D). In LRRK2 G2019S^ki/ki^ astrocytes, Ezrin is more likely to be phosphorylated and in the open state. This distorted balance of the Ezrin state may explain the morphological defects of LRRK2 G2019S^ki/ki^ astrocytes. This model also aligns with our findings that dampening Ezrin phosphorylation via phospho-dead Ezrin disrupts WT astrocyte morphology but rescues the deficits in LRRK2 G2019S^ki/ki^ astrocytes.

If this model is correct, we would expect that the loss of Lrrk2 function in astrocytes impacts astrocyte morphogenesis, a phenotype that we could rescue by introducing phospho-mimetic Ezrin. To test the impact of Lrrk2 loss in astrocytes, first, we used a knockout (KO)-verified Lrrk2 antibody^69,70^ and confirmed the expression of Lrrk2 protein in cortical mouse astrocytes by immunostaining coronal brain sections from WT or LRRK2 G2019S^ki/ki^ mice. Lrrk2 staining was evident in WT and LRRK2 G2019S^ki/ki^ astrocytes, localizing throughout astrocytic processes (Figure S7A–B). In addition, cortical astrocytes isolated from WT rats express Lrrk2 *in vitro*, which was detected by immunoblotting (Figure S7C–D). In agreement, RNA sequencing databases for cortical cell types in mice and humans also show *lrrk2* mRNA present in astrocytes (Fig. S7C–D).

To test if astrocytic Lrrk2 function is required to control astrocyte morphogenesis, we knocked down Lrrk2 in WT astrocytes *in vivo*, using PALE. We transfected astrocytes with the mCherry-CAAX reporter and either with a control shRNA (shControl) or a shRNA that reduces Lrrk2 expression by ~90% (Figure S7E–F, targets both rat and mouse Lrrk2). Interestingly, similar to LRRK2 G2019S^ki/ki^ astrocytes, shLrrk2- transfected astrocytes had significantly smaller territory volumes (reduced by ~36%) and reduced morphological complexity when compared to shControl astrocytes (Figure 5E–G).

Could phospho-mimetic Ezrin rescue the morphological complexity of shLrrk2-transfected astrocytes? To test this, we performed PALE to introduce shLrrk2 or shControl with a phospho-mimetic Ezrin overexpression vector. Overexpression of phospho-mimetic Ezrin in shControl astrocytes did not change astrocyte territory volume and morphological complexity compared to only shControl-transfected astrocytes (Figure 5E–G). However, expression of phospho-mimetic Ezrin in shLrrk2-containing astrocytes restored astrocyte territory volume and morphological complexity up to the levels of shControl-transfected astrocytes (Figure 5E–G).

These results show that both the gain of Lrrk2 kinase function (in LRRK2 G2019S^ki/ki^ mice) and the loss of Lrrk2 function (shLrrk2) decreases astrocyte complexity and size. In line with our model, we found that the opposite manipulation of Ezrin conformational/phosphorylation states can rescue these morphological phenotypes. Interestingly, in the shControl astrocytes overexpressed with phospho-mimetic Ezrin, we did not observe a reduction in astrocyte territory and morphological complexity. This outcome can be explained by the presence of endogenous Ezrin in its closed conformation, which balances the additional phospho-mimetic Ezrin. These findings reveal that Lrrk2 is crucial to regulate astrocyte morphological complexity via balancing ERM phosphorylation *in vivo*.

### LRRK2 G2019S mutation interrupts Ezrin interactomes in astrocytes

**Figure 6:**
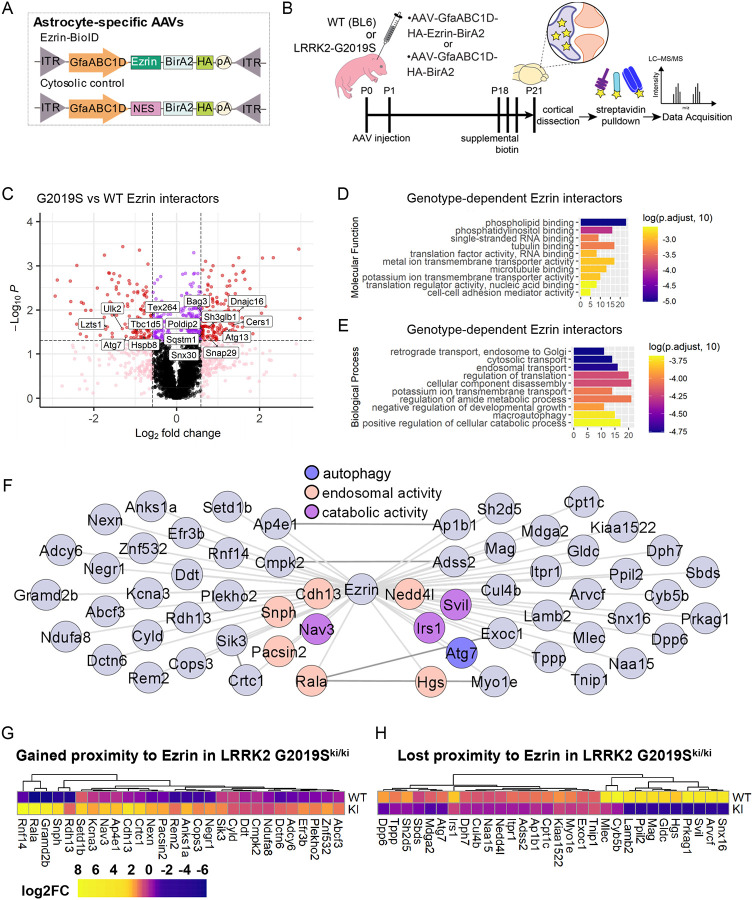
LRRK2 G2019S alters the interactome of astrocytic Ezrin *in vivo*. (A) astrocyte-specific AAVs (serotype PHP.eB) were used to express Ezrin or nonspecific cytosolic control. NES, nuclear export sequence; ITR, inverted terminal repeats; GfaABC1D truncated GFAP promoter; HA, hemagglutinin tag; pA, polyadenylation. (B) outline of the experimental paradigm. n = 3 biological replicates per construct per genotype (1 replicate = 2 animals per pooled sample). (C) Volcano plot showing the differential abundance of proteins detected by Astro-Ezrin-BioID in WT and LRRK2 G2019S^ki/ki^ cortices. (D-E) Bars show the top 10 most significant Gene Ontology (GO) terms, ordered by lowest adjusted p-value, for the proteins differentially detected by Astro-Ezrin-BioID in WT compared to LRRK2 G2019S^ki/ki^ (D) Molecular function (E) Biological Process. (F) The interaction network depicts 58 high-confidence proteins that gained or lost proximity to Ezrin in LRRK2 G2019S^ki/ki^ compared to WT mice. (G-H) heatmaps depict fold-change in abundance (Astro-Ezrin-BioID / Asto-Cyto-BioID) of proteins with high confidence changed proximity to Ezrin in LRRK2 G2019S^ki/ki^ astrocytes.

What are the molecular mechanisms by which ERM hyperphosphorylation stunts astrocyte morphology in LRRK2 G2019S^ki/ki^ astrocytes? To address this question, we identified possible interactors of Ezrin in WT and LRRK2 G2019S^ki/ki^ astrocytes by *in vivo* astrocyte-specific proximity labeling. To do so, we used two virally encoded astrocyte-specific biotin ligase (BirA2) probes: Ezrin fused with BirA2 (Astro-Ezrin-BirA2) and soluble BirA2 with a nuclear export sequence (Astro-Cyto-BirA2) (Figure 6A). The viruses were delivered using intracortical AAV injections to P0–2 WT and LRRK2 G2019S^ki/ki^ mouse pups. AAV-injected mice were given subcutaneous biotin injections starting at P18 for 3 days, and cortices were collected at P21 (Figure 6B). Biotinylated proteins were detected in astrocytes using immunohistochemistry and immunoblotting for HA and Streptavidin (Figure S8A–C).

Biotinylated proteins from cortices were affinity-purified and analyzed using quantitative liquid chromatography-tandem mass spectrometry (LC-MS) (Figure 6B). Across the samples, we identified 30,815 unique peptides corresponding to 4,081 unique proteins. Principle component analyses (PCA) of iBioID results showed that Cyto-BioID (control) and Ezrin-BioID (bait) samples cluster separately, and Ezrin-BioID samples from WT or LRRK2 G2019S^ki/ki^ astrocytes form distinct clusters (Figure S9A). We detected Radixin and Moesin, known Ezrin family proteins, to be significantly enriched in the Ezrin-BioID sample compared to Cyto-BioID (Figure S9B)^71^.

To delineate genotype-specific interactors of Ezrin, we performed a comparative analysis of putative Ezrin interactors from WT versus LRRK2 G2019S^ki/ki^ mice. Astro-Ezrin-BioID detected 344 proteins at a significantly different abundance (fold-change > 1.5 or < −1.5, *p* < 0.05) between genotypes. 190 were found to gain proximity to Ezrin in LRRK2 G2019S^ki/ki^ astrocytes; whereas 154 lost Ezrin proximity (Figure 6C; Figure S9C–D).

To determine how changes in the Ezrin interactome may impact molecular processes and cellular functions in astrocytes, we applied Gene Ontology (GO) analysis to the 344 proteins that were differentially enriched between genotypes. We found that terms for molecular functions related to phospholipid binding, microtubule binding, and membrane transporter and cell-adhesion mediator activity were over-represented in differentially enriched proteins (Figure 6D). This aligns with our model that the LRRK2 G2019S mutation would shift Ezrin towards its phosphorylated/open state, which is associated with membranes by binding lipids and membrane proteins. Unexpectedly, when we applied GO-term analyses to biological processes, we found terms such as catabolic processes, macroautophagy, negative regulation of developmental growth, and intracellular transport to be overrepresented (Figure 6E). These findings indicated a role for Ezrin in these biological processes, which are altered by the LRRK2 G2019S mutation.

Previous studies in neurons and other cell types demonstrated macroautophagy deficits when the LRRK2 G2019S mutation is present^72^. Therefore, we next analyzed global changes in the cytosol induced by the LRRK2 G2019S mutation by comparing the Astro-Cyto-BioID datasets of all astrocytic proteins across genotypes. There were 195 proteins that were differentially enriched in the Cyto-BioID depending on the genotype (78 gained and 117 lost in LRRK2 G2019S^ki/ki^, Figure S9E). GO-term analysis of differentially detected cytosolic proteins revealed an enrichment of proteins associated with phospholipid binding and vesicle trafficking (Figure S9F–G). Macroautophagy, catabolic processes, and related GO-terms did not enrich in Cyto-BioID, suggesting that changes in these biological processes that we found in the Ezrin-BioID are specifically related to alterations in Ezrin function in the LRRK2 G2019S^ki/ki^ astrocytes.

iBioID captures proteins that are in close proximity to the bait but does not guarantee direct protein-protein interactions. To identify potential direct interactors of Ezrin which are differentially regulated by LRRK2 G2019S mutation, we compared protein abundance in Astro-Ezrin-BioID to Astro-Cyto-BioID within each genotype (Ezrin/Cyto fold -change > 1.5, *p* > 0.05, 346 proteins in WT and 335 proteins in LRRK2 G2019S^ki/ki^). To generate high-confidence lists of interactors that gained or lost proximity to Ezrin in LRRK2 G2019S^ki/ki^ astrocytes, we set a second requirement for proteins with fold-change > 1.5 or < −1.5 between LRRK2 G2019S^ki/ki^ and WT Astro-Ezrin-BioID. Using these filters, we narrowed down our list to 28 as “gained proximity to Ezrin in LRRK2 G2019S astrocytes” and 30 as “lost proximity to Ezrin in LRRK2 G2019S astrocytes” (Figure 6F–H; Supplemental Table 2).

Taken together, these data provide a comprehensive map of Ezrin interactomes in WT and LRRK2 G2019S^ki/ki^ mouse astrocytes *in vivo*. This discovery-based approach reveals unexpected changes in Ezrin interactions with proteins that function in catabolic processes and autophagy in the astrocytes of LRRK2 G2019S^ki/ki^ mice. Thus, it points us towards a molecular mechanism underlying the role of Lrrk2-Ezrin signaling pathways in astrocyte morphogenesis.

### Atg7 is an Ezrin interactor that is required for proper astrocyte morphogenesis

Among the high-confidence astrocytic Ezrin interactors, we identified autophagy-related gene 7 (Atg7), a crucial autophagy regulator. Atg7 was highly enriched (~2.3-fold) in the Ezrin-BioID fraction of WT astrocytes but was less abundant (−2.0-fold) in the Ezrin-BioID fraction of LRRK2 G2019S^ki/ki^ astrocytes compared to the Cyto-BioID. Atg7 encodes an E1-like ubiquitin-activating enzyme and plays a crucial role in the initiation of autophagy^73^. Studies in mice and humans have also pointed to the dysregulation of Atg7 in neurological disorders^74^. However, little is known about the role of Atg7 in astrocytes, and whether it can directly interact with Ezrin and regulate astrocyte morphology is unknown.

**Figure 7:**
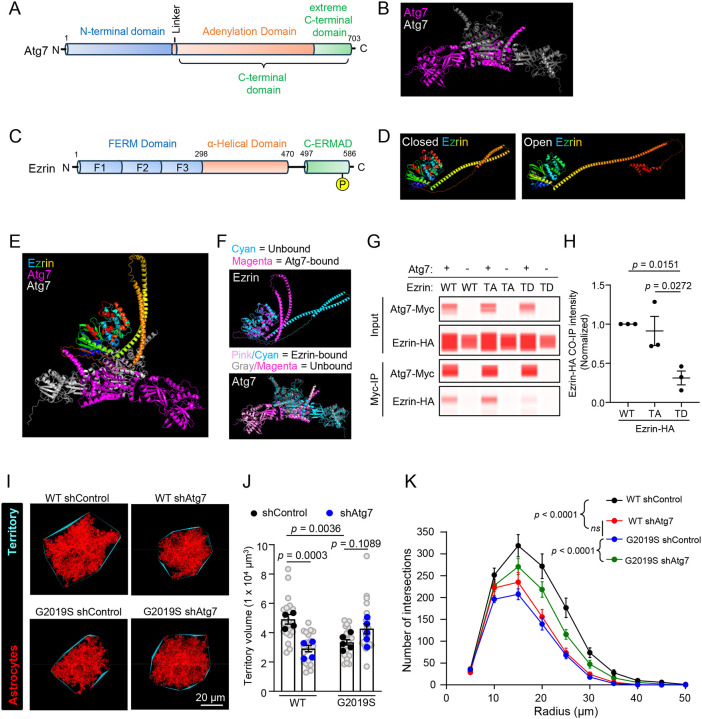
Interaction Between Atg7 and Ezrin Depends on Ezrin’s Phosphorylation State. (A) Schematic of domains within Atg7. (B) Predicted model of Atg7 homodimer. (C) Schematic of domains within Ezrin. (D) Model of the predicted structure of Ezrin in its closed or open conformation. (E) Model of the predicted interaction between Atg7 homodimer and Ezrin. (F) (Upper) Model of the predicted structural changes of Ezrin when bound to Atg7. (Lower) Model of the predicted structural changes of Atg7 when bound to Ezrin. (G) Co-immunoprecipitation of Ezrin-HA by Atg7-Myc pull down. Immunoblot of HEK293T cell lysates overexpressing WT Ezrin, phospho-dead Ezrin, or phospho-mimetic Ezrin with or without Atg7-Myc. Ezrin is detected by an anti-HA antibody, while Atg7 is detected by an anti-Myc antibody. (H) Quantification of Ezrin co-immunoprecipitation signal intensity in (G). Signals corresponding to Ezrin were first normalized to that for WT Ezrin. Statistical significance was determined by One-way ANOVA [F (2, 6) = 9.932, *p* = 0.0125]. Tukey’s multiple comparisons test revealed a significant difference between WT Ezrin and phospho-mimetic Ezrin (*p* = 0.0151, 95% C.I. = [0.1724, 1.205]) and between phospho-dead Ezrin and phospho-mimetic Ezrin (*p* = 0.0272, 95% C.I. = [0.08525, 1.118]) and no differences between WT Ezrin and phospho-dead Ezrin (*p* = 0.8658, 95% C.I. = [−0.4292, 0.6035]), alpha = 0.05. n = 3 independent experiments. (I) Images of ACC and MOp L2–3 WT and LRRK2 G2019S^ki/ki^ astrocytes at P21 expressing shControl-PB-mCherry-CAAX or sh*Atg7*-PB-mCherry-CAAX. Astrocyte territory in cyan. Scale bar, 10 μm. (J) Average territory volumes of P21 astrocytes. n = 20 (WT shControl; 4 mice), 21 (WT sh*Atg7*; 4 mice), 25 (LRRK2 G2019S^ki/ki^ shControl; 5 mice), 23 (LRRK2 G2019S^ki/ki^ sh*Atg7*; 5 mice). Statistical significance was determined by Nested one-way ANOVA [F (3, 28) = 9.484, *p* = 0.0002]. Bonferroni multiple comparisons revealed a significant difference between WT shControl and LRRK2 G2019S^ki/ki^ shControl (*p* = 0.0036, 95% C.I. = [0.4504, 2.701]), between WT shControl and WT sh*Atg7* (*p* = 0.0003, 95% C.I. = [0.8655, 3.176]), and between WT sh*Atg7* and LRRK2 G2019S^ki/ki^ sh*Atg7* (*p* = 0.0109, 95% C.I. = [−2.502, −0.2662]), and no differences between WT shControl and LRRK2 G2019S^ki/ki^ sh*Atg7* (*p* = 0.4294, 95% C.I. = [−0.4935, 1.766]), between LRRK2 G2019S^ki/ki^ shControl and WT sh*Atg7* (*p* = 0.6977, 95% C.I. = [−0.6684, 1.558]), and between LRRK2 G2019S^ki/ki^ shControl and LRRK2 G2019S^ki/ki^ sh*Atg7* (*p* = 0.1089, 95% C.I. = [−2.026, 0.1480]), alpha = 0.05. Data points are mouse averages. (K) Quantification of astrocyte branching complexity. n = 20 (WT shControl; 4 mice), 21 (WT sh*Atg7*; 4 mice), 26 (LRRK2 G2019S^ki/ki^ shControl; 5 mice), 23 (LRRK2 G2019S^ki/ki^ sh*Atg7*; 5 mice). Two-way ANOVA for repeated measures. Main effects of conditions [F (2.324, 472.6) = 43.1, *p* < 0.05] and radius [F (9, 250) = 301.8, *p* < 0.05] and interaction [F (27, 610) = 4.539, *p* < 0.05]. Bonferroni multiple comparisons revealed a significant difference between WT shControl and LRRK2 G2019S^ki/ki^ shControl (*p* < 0.0001, 95% C.I. = [34.87, 66.02]), between WT shControl and WT sh*Atg7* (*p* < 0.0001, 95% C.I. = [26.39, 58.96]), between WT shControl and LRRK2 G2019S^ki/ki^ sh*Atg7* (*p* = 0.0021, 95% C.I. = [6.359, 41.23]), between LRRK2 G2019S^ki/ki^ shControl and LRRK2 G2019S^ki/ki^ sh*Atg7* (*p* < 0.0001, 95% C.I. = [−36.99, −16.31]), and between WT sh*Atg7* and LRRK2 G2019S^ki/ki^ sh*Atg7* (*p* = 0.0006, 95% C.I. = [−31.62, −6.15]), and no difference between LRRK2 G2019S^ki/ki^ shControl and WT sh*Atg7* (*p* = 0.3353, 95% C.I. = [−18.53, 2.994]).

First, to examine the putative interaction between Ezrin and Atg7, we used AlphaFold-2.0 Multimer^75^ to model the structure of these proteins alone or in complex with each other. Atg7, which functions as a homodimer, has two globular domains: An N-terminal domain and a C-terminal domain that are separated by a short flexible linker. The C-terminal domain is further divided into the adenylation and extreme C-terminal domains (Figure 7A–B)^76^. Ezrin comprises an N-terminal FERM (four-point one, Ezrin, Radixin, Moesin) domain (subdivided into three subdomains- F1, F2, and F3), α-helical domain, and a C-terminal ERM Association domain (C-ERMAD) (Figure 7C)^67^. When unphosphorylated, Ezrin remains in a closed state where N and C terminal domains interact, and the central alpha-helical hinge region bends to form a “clamshell-like” structure. Upon phosphorylation of T567 and binding to PIP2, Ezrin acquires an open conformation^77^. We also modeled both conformations of Ezrin using Alpha-fold (Figure 7D; see Methods for details of modeling).

We additionally utilized Alpha-fold 2.0 multimer to predict the structure of the complex formed between the closed conformation of Ezrin and the homodimer of Atg7 (Figure 7E). Alpha-fold 2.0 multimer, in conjunction with CABS-Flex 2.0 and PyMol, were used to predict and assess conformational changes in Atg7 and Ezrin after binding each other (Figure 7F). When Ezrin binds Atg7, it is predicted to undergo drastic conformational changes in its α-helical domain, which is pulled towards the ERMAD and FERM domains, whereas the changes in the Atg7 dimer structure after Ezrin binding are minimal (Figure 7F). These modeling analyses strongly suggested a direct interaction between Ezrin and Atg7, which we set out to test empirically next.

To probe for an interaction between Ezrin and Atg7, we performed co-immunoprecipitation (Co-IP) in HEK293T cells overexpressing Myc-tagged Atg7 (Atg7-Myc) and HA-tagged WT Ezrin. To understand how the open or closed conformation of Ezrin would impact the Atg7 interaction, we also conducted Co-IPs with modulation of Ezrin phosphorylation levels: phospho-mimetic T567D (“TD”, open) and phospho-dead T567A (“TA”, closed) Ezrin. We found that WT Ezrin or phospho-dead Ezrin coimmunoprecipitated with Myc-Atg7, indicating that Atg7 interacts with WT Ezrin or phospho-dead Ezrin (Figure 7G). However, the interaction between Atg7 and phospho-mimetic Ezrin was significantly reduced (Figure 7H). Together with the structural modeling, these results suggest that Atg7 interacts directly with Ezrin in a phosphorylation/conformation-dependent manner. These findings align well with our Ezrin BioID data (Figure 6), in which Atg7 lost interaction with Ezrin in LRRK2 G2019S^ki/ki^ astrocytes. These findings also indicate that Atg7 may play a role in regulating astrocyte morphogenesis, and this function of Atg7 may be altered in LRRK2 G2019S^ki/ki^ astrocytes.

Due to the impaired interaction between Atg7 and Ezrin when Ezrin is in its phospho-mimetic (open) state, we hypothesized that altered Atg7 function contributes to impaired astrocyte morphology in LRRK2 G2019S^ki/ki^ mice. To test this hypothesis, we developed a short hairpin RNA (shRNA) that targets both rat and mouse *Atg7*, which effectively reduced *Atg7* mRNA in mouse cells (Figure S10A). Knockdown of Atg7 in rat astrocytes significantly reduced neuron-induced astrocyte morphogenesis *in vitro* in a neuron-astrocyte co-culture assay (Figure S10B–C).

To elucidate the necessity of Atg7 in regulating astrocyte morphology *in vivo*, we introduced shControl or shAtg7 into a sparse population of cortical astrocytes in WT and LRRK2 G2019S^ki/ki^ mice by using PALE. We found that Atg7 knockdown in WT astrocytes led to a significant reduction in astrocyte territory volume and morphological complexity, resembling the LRRK2 G2019S^ki/ki^ astrocytes transfected with shControl (Figure 7I–K). Atg7 knockdown in LRRK2 G2019S^ki/ki^ astrocytes did not further reduce their territory volume (Figure 7J) but significantly improved their morphological complexity (Figure 7K). Overall, these data reveal an essential role for Atg7 in astrocyte morphological complexity and suggest that disruptions in Atg7 function in astrocytes contribute to the altered astrocyte morphology observed in LRRK2 G2019S^ki/ki^ mice.

## DISCUSSION

Here, we describe a previously unknown role for Lrrk2, a PD-linked gene, in controlling astrocyte morphological complexity by regulating the phosphorylation state of ERM proteins. In the presence of LRRK2 G2019S mutation, we found enhanced ERM phosphorylation in human and mouse cortical astrocytes. ERM hyperphosphorylation in the LRRK2 G2019S background is specific to a subset of the ACC and MOp astrocytes of juvenile and adult mice. The hyperphosphorylation of ERM proteins in these regions occurred concurrently with dysregulated astrocytic morphology and synapse numbers and activity. These observations prompted us to test how shifting the balance of the ERM phosphorylation status in astrocytes impacts astrocyte morphology and synaptic connectivity. We found that overexpression of a phospho-dead Ezrin, specifically in astrocytes, is sufficient to rescue both the morphological deficits of astrocytes themselves as well as the number and function of excitatory synapses within the ACC of the LRRK2 G2019S^ki/ki^ mice. However, the same manipulation is detrimental in WT astrocytes. Taken together, these results support a model that Lrrk2 activity in astrocytes regulates a tight balance between phosphorylated and unphosphorylated ERM proteins in astrocytes, and pushing this balance in either direction impairs astrocytes’ morphology and synaptic functions.

### How does ERM phosphorylation control astrocyte morphology and synaptogenic functions?

The canonical function of ERM proteins is linking the actin cytoskeleton with the plasma membrane, thus allowing cellular process outgrowth and morphogenesis^64^. Our findings suggest that disruptions in the interaction between Ezrin and a core autophagy-related protein, Atg7, contribute to the altered astrocyte morphology observed in LRRK2 G2019S^ki/ki^ mice. *In vivo* Ezrin interactome in astrocytes included several other proteins related to autophagy, which changed their proximity to Ezrin in the LRRK2 G2019S^ki/ki^ astrocytes, compared to WT astrocytes. These observations indicate that dysregulated autophagy due to ERM hyperphosphorylation may underlie the deficits we observed in astrocytes.

Another intriguing observation is that, *in vivo*, knocking down Atg7 in WT astrocytes reduces astrocyte morphological complexity. Atg7 is a homodimeric E1-like enzyme that is essential for autophagy, a process crucial for maintaining cellular homeostasis by degrading and recycling damaged organelles and proteins^73^. The presence of autophagic dysregulation in the context of neurodegenerative diseases, including PD-associated LRRK2 mutations, is well-documented^31,78,72^. However, whether autophagy plays a role in astrocyte morphology and its synaptogenic functions is not known. Moreover, how LRRK2 G2019S mutation impacts autophagy in astrocytes is not yet elucidated. It is tempting to speculate that the depletion of Atg7 disrupts autophagic flux, accumulating damaged organelles and proteins within astrocytes, potentially compromising their structural integrity and function.

However, even without Atg7, other forms of autophagy can still occur^79,80^, suggesting that the role of Atg7 in astrocyte morphogenesis may be independent of its canonical role in autophagy. For example, Atg7 is implicated in various endocytic pathways, including LC3-associated endocytosis and phagocytosis, processes crucial for cellular clearance and process remodeling^81,82^. Our findings also pinpoint a role for Ezrin (and other ERM proteins) in autophagy regulation. In agreement, there is emerging evidence linking Ezrin to molecular pathways regulating autophagy^83^. Through molecular modeling and biochemical methods, we found evidence for a direct interaction between Atg7 and unphosphorylated Ezrin. Future studies would be highly fruitful if we could determine how this interaction impacts astrocyte cell biology.

Astrocytes control synapse formation and function through cell adhesion molecules and secreted proteins that signal to neurons^14^. It is plausible that ERM phosphorylation regulates synaptogenic protein secretion from astrocytes via autophagy-related pathways. Another alternative explanation is the role of astrocytic phospho-ERM proteins in controlling synapse elimination via phagocytosis by astrocytes. In agreement with this possibility, a recent study found that synapses are more likely to be eliminated if phospho-ERM proteins were localized to peri-synaptic astrocyte processes in ALS patient brains and mouse models^84^. Future research investigating the complex relationships between Lrrk2, ERM, and autophagy in astrocytes will be necessary to test these intriguing possibilities.

### Implications for Therapeutic Development

LRRK2 G2019S^ki/ki^ mice do not recapitulate motor symptoms of PD because there is no detectable dopaminergic neuron loss in these mice^30,43^. However, these mice serve as good models for the non-motor (prodromal) PD symptoms seen in LRRK2 G2019S carriers^3^. These non-motor symptoms, which arise long before motor dysfunction in PD, indicate aberrant synaptic connectivity within multiple brain circuits, including the cortex, long before the death of dopaminergic neurons^85^.

In agreement, LRRK2 G2019S^ki/ki^ mice display early circuit dysfunction and non-motor symptoms akin to prodromal PD. Previous studies showed that LRRK2 G2019S^ki/ki^ mice have deficits in attention and goal-directed learning and differential response to stress^33,35,86^. Our findings showing synaptic deficits in the ACC of LRRK2 G2019S^ki/ki^ mice (Figure 2–3) fit well with these studies because the ACC is a brain region that controls attention, goal-directed actions, and mood^87,88^. Furthermore, we observed synaptic deficits in the ACC of LRRK2 G2019S^ki/ki^ mice concurrently with astrocyte morphological deficits located in the ACC and MOp.

Why do ERM phosphorylation and synaptic deficits happen in specific brain regions of LRRK2 G2019S^ki/ki^ mice? Adult cortical astrocytes are heterogeneous in their gene expression, morphology, and function^89,90^. Interestingly, cortical astrocyte heterogeneity is controlled by layer-specific neuron heterogeneity^89,90^. It is plausible that neurons and astrocytes in different cortical regions have varying levels of LRRK2 and diverse susceptibilities to LRRK2 mutations. The ACC and MOp may heavily depend on LRRK2 to maintain synaptic integrity and astrocyte function. Future studies investigating the impacts of LRRK2 G2019S in cortical neurons and astrocytes’ transcriptional profiles may help explain why region-specific impact of this mutation in astrocytes.

In sum, our findings reveal a critical function for Lrrk2 in astrocyte development and synaptic connectivity, suggesting that Lrrk2-mediated ERM hyperphosphorylation in astrocytes is an early mediator of PD pathogenesis. This astrocytic dysfunction could directly contribute to synaptic deficits observed in the prodromal stages of PD. Moreover, our data indicate that restoring astrocyte function, even post-development could be a promising strategy for treating excitatory synaptic pathology. Identifying the molecular mechanisms by which Lrrk2 controls ERM phosphorylation and the specific phosphatases involved could lead to new therapeutic targets for PD. Future studies aimed to elucidate the molecular mechanisms through which Lrrk2 controls ERM phosphorylation and to identify the phosphatases required to that maintain the tight balance of ERM phosphorylation in astrocytes are likely to reveal new targets for PD therapeutics.

## STAR METHODS

### RESOURCE AVAILABILITY

#### Lead contact

Further information and requests for resources and reagents should be directed to and will be fulfilled by the lead contacts, Shiyi Wang (shiyi.wang@duke.edu) and Cagla Eroglu (cagla.eroglu@duke.edu).

#### Materials availability

The reagents generated in this study are available without restriction.

#### Data and code availability

The data generated during this study are available from the lead contact upon request. The proteomic data is uploaded in the repository Massive (massive.ucsd.edu), accession number: MSV000095133

### EXPERIMENTAL MODELS AND SUBJECT DETAILS

#### Animals

All mice and rats were used in accordance with the Institutional Animal Care and Use Committee (IACUC) and the Duke Division of Laboratory Animal Resources (DLAR) oversight (IACUC Protocol Numbers A147–17-06 and A117–20-05). All mice and rats were housed under typical day/night conditions of 12-hour cycles. LRRK2 G2019S^ki/ki^ mice (RRID: IMSR_TAC:13940) were obtained through Taconic. This model was generated on the C57BL6/NTac background. It has been maintained on that pure background in Taconic. Aldh1l1-EGFP (RRID: MMRRC_011015-UCD) mice were obtained through MMRRC and backcrossed on a C57BL/6J background. Wild-type CD1 mice used for PALE were purchased through Charles River Laboratories (RRID: IMSR_CRL:022).

Mice and rats were used for experiments as specified in the text and figure legends. For all experiments, age, and sex-matched mice were randomly assigned to experimental groups based on genotypes. Mice of both sexes were included in the analysis, and we did not observe any influence or association of sex on the experimental outcomes.

#### Primary cortical neuron isolation and culture

Purified (glia-free) rat cortical neurons were prepared as described previously^40^. Briefly, cortices from P1 rat pups of both sexes (Sprague Dawley, Charles River Laboratories, SD-001) were micro-dissected, digested in papain (~7.5 units/ml) at 33°C for 45 minutes, triturated in low and high ovomucoid solutions, resuspended in panning buffer (DPBS (GIBCO 14287) supplemented with BSA and insulin) and passed through a 20 μm mesh filter (Elko Filtering 03–20/14). Filtered cells were incubated on negative panning dishes coated with Bandeiraea Simplicifolia Lectin 1 (x2), followed by goat anti-mouse IgG+IgM (H+L) (Jackson ImmunoResearch 115–005-044), and goat anti-rat IgG+IgM (H+L) (Jackson ImmunoResearch 112–005-044) antibodies, then incubated on positive panning dishes coated with mouse anti-L1 (ASCS4, Developmental Studies Hybridoma Bank, Univ. Iowa) to bind cortical neurons. Adherent cells were collected by forceful pipetting with a P1000 pipette. Isolated neurons were pelleted (11 minutes at 200 g) and resuspended in serum-free neuron growth media (NGM; Neurobasal, B27 supplement, 2 mM L-Glutamine, 100 U/ml Pen/Strep, 1 mM sodium pyruvate, 4.2 μg/ml Forskolin, 50 ng/mL BDNF, and 10 ng/mL CNTF). 70,000 neurons were plated onto 12 mm glass coverslips coated with 10 μg/ml poly-D-lysine (PDL, Sigma P6407) and 2 μg/ml laminin and incubated at 37°C in 10% CO_2_. On day *in-vitro* (DIV) 2, half of the media was replaced with NGM Plus (Neurobasal Plus, B27 Plus, 100 U/mL Pen/Strep, 1 mM sodium pyruvate, 4.2 μg/ml Forskolin, 50 ng/ml, BDNF, and 10 ng/ml CNTF) and AraC (10 μM) was added to stop the growth of proliferating contaminating cells. On DIV 3, all the media was replaced with NGM Plus. In experiments involving lentivirus infection, 100 μl of supernatant containing lentivirus plus polybrene (1 μg/ml) was added to the AraC NGM mixture on DIV 2 and completely washed out on DIV 3 and replaced with NGM Plus containing 100 ng/ml BDNF. Neurons were fed on DIV 6 and DIV 9 by replacing half of the media with NGM Plus.

#### Primary cortical astrocyte isolation and culture

##### Rat astrocyte isolation and culture

Rat cortical astrocytes were prepared as described previously^40^. P1 rat cortices from both sexes were micro-dissected, papain digested, triturated in low and high ovomucoid solutions, and resuspended in astrocyte growth media (AGM: DMEM (GIBCO 11960), 10% FBS, 10 μM, hydrocortisone, 100 U/ml Pen/Strep, 2 mM L-Glutamine, 5 μg/ml Insulin, 1 mM Na Pyruvate, 5 μg/ml N-Acetyl-L-cysteine). Between 15–20 million cells were plated on 75 mm^2^ flasks (non-ventilated cap) coated with poly-D-lysine and incubated at 37°C in 10% CO_2_. On DIV 3, the removal of non-astrocyte cells was performed by forcefully shaking closed flasks by hand for 10–15 s until only an adherent monolayer of astrocytes remained. AraC was added to the media from DIV 5 to DIV 7 to eliminate contaminating fibroblasts. On DIV 7, astrocytes were trypsinized (0.05% Trypsin-EDTA) and plated into 12-well or 6-well dishes. On DIV 8, cultured rat astrocytes were transfected with shRNA and/or expression plasmids using Lipofectamine LTX with Plus Reagent (Thermo Scientific) per the manufacturer’s protocol. Briefly, 1 μg (12-well) or 2 μg (6-well) total DNA was diluted in Opti-MEM containing Plus Reagent, mixed with Opti-MEM containing LTX (1:2 DNA to LTX), and incubated for 30 minutes. The transfection solution was added to astrocyte cultures and incubated at 37°C for 3 hours. On DIV 10, astrocytes were trypsinized, resuspended in NGM plus, plated (20,000 cells per well) onto DIV 10 neurons, and co-cultured for 48 hours.

##### shRNA plasmids

pLKO.1 Puro plasmids containing shRNA (pLKO.1-shRNA) against mouse/rat *Lrrk2* (sh*Lrrk2*: TRCN0000322193; GGCCGAGTTGTGGATCATATT) and mouse/rat *Atg7* (sh*Atg7*: TRCN0000092163; TATTATTGAGTTCAGAGCTGG), were obtained from the RNAi Consortium (TRC) via Dharmacon. Two scrambled shRNA sequences were generated (GTTGCTGAATGGCGGATCTAT; GTTGCGGTTATGAATAGTACT) and cloned into the pLKO.1 TRC cloning vector^94^ according to Addgene protocols (https://www.addgene.org/protocols/plko/). To generate pLKO.1 shRNA plasmids that express EGFP (pLKO.1-shRNA-EGFP), CAG-EGFP was removed from pLenLox-shNL1-CAG-EGFP^95^ and inserted between Kpn1 and SpeI sites in pLKO.1 Puro, replacing the puromycin resistance gene. pLKO.1 shRNA mCherry plasmids were generated by replacing EGFP with mCherry between KpnI and NheI sites.

##### PiggyBac plasmids

pPB-CAG-EGFP and pGLAST-PBase were a gift from Dr. Joseph Loturco^96^. To generate pPB-CAG-mCherry-CAAX, mCherry-CAAX was inserted between XmaI and NotI restrictions sites to replace EGFP. To insert the hU6 promoter and shRNA in pPB-CAG-mCherry-CAAX, a DNA fragment containing hU6 and shRNA was amplified from pLKO.1-shRNA using Phusion High-Fidelity DNA Polymerase (NEB) with primers that introduced SpeI restriction sites (Forward Primer: GGACTAGTCAGGCCCGAAGGAATAGAAG; Reverse Primer: GGACTAGTGCCAAAGTGGATCTCTGCTG). PCR products were purified, digested with SpeI, and ligated into pPB-CAG-mCherry-CAAX at the SpeI restriction site. An analytical digest with EcoRI followed by sequencing was used to confirm the orientation of the inserted DNA fragment.

##### Ezrin plasmids

pZac2.1-GfaABC1D-Lck-GCaMP6f was a gift from Dr. Baljit Khakh (Addgene plasmid #52924). pZac2.1-GfaABC1D-BioID2-HA was generated by PCR of BioID2 from pAAV-hSyn-BioID2-Linker-Synapsin1a-HA^97^ (primers Fwd: 5’-ctagcctcgagaattcaccatgttcaaaaatcttatttg-3’, Rev: 5’-ccgggtcgactctagatgcgtaatccggtacatcg-3’) and insertion into the EcoRI and XbaI restriction sites of pZac2.1-GfaABC1D-Lck-GCaMP6f using In-Fusion cloning (TaKaRa). pHJ421(pEGFP-Ezrin WT) and pHJ423 (pEGFP-Ezrin T567D) were a gift from Stephen Shaw (Addgene plasmid # 20680 and # 20681). pZac2.1-GfaABC1D-Ezrin WT-BioID2-HA and pZac2.1-GfaABC1D-Ezrin T567D-BioID2-HA were generated by PCR of Ezrin from pHJ421 or pHJ423 (primers Fw: 5’-ctagcctcgagaattcaccatgccgaaaccaatca-3’, Rv: 5’-tgaacatggtgaattccgacagggcctcgaactcg-3’) and insertion into the EcoRI restriction sites of pZac2.1-GfaABC1D-BioID2, respectively. To generate pZac2.1-GfaABC1D-Ezrin T567A-BioID2-HA plasmid, Q5^®^ Site-Directed Mutagenesis Kit (NEB) was used with mutagenesis primers (Fwd: CAAGTACAAGGCGCTGCGGCAGA; Rev: TCCCGGCCTTGCCTCATG)

##### Lentivirus production and transduction

Lentiviruses containing shRNA targeting vectors were produced to test the knockdown efficiency of shRNA constructs in cultured primary astrocytes or to bulk transduce neurons with shRNA and GFP. To produce lentivirus, HEK293T cells were transfected with a pLKO.1 shRNA Puro targeting plasmid (for astrocyte transduction), an envelope plasmid (VSVG), and a packaging plasmid (dR8.91) using X-tremeGENE (Roche). One day after transfection, the media was replaced with AGM (for astrocyte transduction), and media containing lentivirus was collected on days 2 and 3 post-transfection. To assess the knockdown efficiency of shRNAs in astrocytes, rat primary astrocytes at DIV 7 were plated in 6-well dishes in 2 ml of AGM. On DIV 8, 1 ml of AGM was removed, and 500 μl of fresh AGM was added along with 500 μl of lentivirus-containing media and 1 μg/ml polybrene. Cultured astrocytes were treated with puromycin (1 μg/ml) from DIV 10–15 to select for transduced cells. Cultured astrocytes were lysed at DIV 15 for protein extraction and Western blot analysis.

##### Immunocytochemistry

Astrocyte-neuron co-cultures on glass coverslips were fixed on DIV 12 with warm 4% PFA for 7 minutes, washed 3 times with PBS, blocked in a blocking buffer containing 50% normal goat serum (NGS) and 0.4% Triton X-100 for 30 minutes, and washed in PBS. Samples were then incubated overnight at 4°C in primary antibodies diluted in blocking buffer containing 10% NGS, washed with PBS, incubated in Alexa Fluor conjugated secondary antibodies (Life Technologies) for 2 hours at room temperature, and washed again in PBS. Coverslips were mounted onto glass slides (VWR Scientific) with Vectashield mounting media containing DAPI (Vector Labs), sealed with nail polish, and imaged on an AxioImager M1 (Zeiss) fluorescence microscope. Images of healthy astrocytes with strong expression of fluorescent markers that did not overlap with other fluorescent astrocytes were acquired at 40x magnification in red, green, and/or DAPI channels using a CCD camera. Astrocyte morphological complexity was analyzed in FIJI using the Sholl analysis plugin^98^, as described previously^40^ (https://github.com/Eroglu-Lab/In-Vitro-Sholl). Statistical analyses were conducted using a custom code in R (https://github.com/Eroglu-Lab/In-Vitro-Sholl). A mixed-effect model with multiple comparisons made using the Tukey post-test was used for Sholl analysis to account for the variability per experiment as a random effect to evaluate differences between the conditions. The exact number of independent experiments and the exact number of cells analyzed are indicated in the figure legend for each experiment. To ensure the health of astrocyte-neuron co-cultures, the peak for the number of astrocyte intersections must be greater than or equal to 25 in the control condition. We imaged non-overlapping astrocytes that contained a single nucleus (DAPI stain) and expressed consistent fluorescent markers and Ezrin constructs according to the experimental conditions.

##### Postnatal astrocyte labeling by electroporation (PALE)

Late P0/early P1 mice were sedated by hypothermia until anesthetized, and 1 μl of plasmid DNA mixed with Fast Green Dye was injected into the lateral ventricle of one hemisphere using a pulled glass pipette (Drummond). For shRNA knockdown experiments in wild-type CD1 mice, the 1 μl of DNA contained 1 μg of pGLAST-PBase and 1 μg of pPB-shRNA-mCherryCAAX was injected. To label astrocytes in WT and LRRK2 G2019S^ki/ki^ mice, the 1 μl of DNA contained 1 μg of pGLAST-PBase and 1 μg of pPB-mCherry-CAAX was injected per mouse.

For PALE-mediated overexpression of phospho-mimetic Ezrin in shRNA knockdown experiments, 0.5 μg pGLAST-PBase, 0.5 μg pPB-shRNA-mCherryCAAX, and 1 μg pZac2.1-GfaABC1D-Ezrin T567D-BioID2-HA were injected in a total volume of 1 μl. For phospho-dead Ezrin overexpression in WT and LRRK2 G2019S^ki/ki^ mice, 0.5 μg pGLAST-PBase, 0.5 μg of pZac2.1-gfaABC1D-mCherry-CAAX and 1 μg of pZac2.1-GfaABC1D-Ezrin T567A-BioID2-HA were injected in a total volume of 1 μl. Following DNA injection, electrodes were oriented with the positive terminal above the frontal cortex and the negative terminal below the chin, and 5 discrete 50 ms pulses of 100 V spaced 950 ms apart were applied. Pups were recovered on a heating pad, returned to their home cage, and monitored until collection at P21. The number of mice used for each experiment is indicated in the figure legends. All animals appeared healthy at the time of collection. Brain sections were examined for the presence of electroporated cells before staining.

##### Adeno-associated virus (AAV) production and administration

Purified AAVs were produced as described previously^99^. Briefly, HEK293T cells were transfected with pAd-DELTA F6, serotype plasmid AAV PHP.eB, and AAV plasmid (pZac2.1-GfaABC1D-Ezrin-BioID2-HA or pZac2.1-GfaABC1D-Ezrin T567A-BioID2-HA). Three days after transfection, cells were collected in 15 mM NaCl, 5 mM Tris-HCl, pH 8.5, and lysed with repeat freeze-thaw cycles followed by treatment with Benzonase (Novagen 70664) at 37°C for 30 minutes. Lysed cells were pelleted by centrifugation, and the supernatant, containing AAVs, was applied to an Optiprep density gradient (Sigma D1556, 15%, 25%, 40%, and 60%) and centrifuged at 67,000 rpm using a Beckman Ti-70 rotor for 1 hour. The AAV-enriched fraction was isolated from between 40% and 60% iodixanol solution and concentrated by repeated washes with sterile PBS in an Amicon Ultra-15 filtration unit (NMWL: 100 kDa, Millipore UFC910008) to a final volume of ~100 μl and aliquoted for storage at −80°C. 9-week-old WT or LRRK2 G2019S^ki/ki^ mice placed in a stereotaxic frame were anesthetized through inhalation of 1.5% isofluorane gas. 10 μl of purified AAVs having a titer of ~1 × 10^12^ GC/ml was introduced into the mouse brain intravenously by injection into the retro-orbital sinus. After 3 weeks at 12-week-old, mice were anesthetized with 200 mg/kg Tribromoethanol (Avertin) and transcardially perfused with TBS/Heparin and 4% paraformaldehyde (PFA) at room temperature (RT). Harvested brains were post-fixed overnight in 4% PFA, cryoprotected in 30% sucrose, and the brain blocks were prepared with O.C.T. (TissueTek) to store at −80°C. 30 μm thick brain sections were obtained through cryosectioning using a Leica CM3050S (Leica, Germany) vibratome and stored in a mixture of TBS and glycerol at −20°C for further free-float antibody staining procedures.

##### Immunohistochemistry on mouse brain sections

Mice used for immunohistochemistry were anesthetized with 200 mg/kg Avertin and perfused with TBS/Heparin and 4% PFA. Brains were collected and post-fixed in 4% PFA overnight, cryoprotected in 30% sucrose, frozen in a solution containing 2 parts 30% sucrose and 1-part O.C.T. (TissueTek), and stored at −80°C. Floating coronal tissue sections of 30 μm, 40 μm or 100 μm thickness were collected and stored in a 1:1 mixture of TBS/glycerol at −20°C. For immunostaining, sections were washed in 1x TBS containing 0.2% Triton X-100 (TBST), blocked in 10% NGS diluted in TBST, and incubated in primary antibody for 2–3 nights at 4°C with gentle shaking. Primary antibodies used were anti-LRRK2 (Rabbit, 1:500; ab133474, Abcam), HA (Rat, 1:500; 11867423001, Roche), phospho-ERM (Rabbit, 1:500; 3141, Cell Signaling), Sox9 (Rabbit, 1:500; AB5535, Millipore), GFAP (Rabbit, 1:500; Z0334, Agilent DAKO), VGluT1 (Guinea pig, 1:2000; 135304, Synaptic Systems), PSD95 (Rabbit, 1:300; 51–6900, Innovative Research), VGAT (Guinea pig, 1:1000; 131004, Synaptic Systems), and Gephyrin (Rabbit, 1:1000; 147011, Synaptic Systems). Following the primary incubation, sections were washed in TBST, incubated in Alexa Fluor conjugated secondary antibodies diluted 1:200 (Life Technologies) for 2–3 hours at room temperature, washed with TBST, and mounted onto glass slides using a homemade mounting media (90% Glycerol, 20 mM Tris pH 8.0, 0.5% n-Propyl gallate) and sealed with nail polish. For DAPI staining, DAPI (1:50,000) was added to the secondary antibody solution for the final 10 minutes of incubation. Images were acquired with an Olympus FV 3000 microscope.

##### Mouse astrocyte territory volume analysis

To assess the territory volume of individual astrocytes in the mouse cortex, 100 μm-thick floating sections containing anterior cingulate cortex (ACC) and primary motor cortex (MOp) astrocytes labeled sparsely via PALE with mCherry-CAAX were collected. High-magnification images containing an entire astrocyte (50–60 μm z-stack) were acquired with an Olympus FV 3000 microscope with the 60x objective. Criteria for data inclusion required that the entirety of the astrocyte could be captured within a single brain section and that the astrocyte was in L2–3 of the ACC or MOp. Astrocytes in which the entire astrocyte could not be captured within the section or was in other layers or outside of the ACC or MOp were not imaged. Imaged astrocytes were analyzed using Imaris Bitplane software as described previously^40^. Briefly, surface reconstructions were generated, and the Imaris Xtensions “Visualize Surface Spots” and “Convex Hull” were used to create an additional surface render representing the territory of the astrocyte. The volume of each territory was recorded, and astrocyte territory sizes from biological replicates were analyzed across experimental conditions using a nested two-way ANOVA followed by the Bonferroni posthoc test. For 3D Sholl analysis of individual PALE astrocytes, we first loaded images onto Imaris and then created a surface. After generating the surface of astrocytes, we created filaments using ‘Add new filament (leaf icon)’. For the quantification of complexity, we clicked on the gear tool on Imaris to display Sholl intersections. The number of animals and cells/animals analyzed are specified in the figure legend for each experiment.

#### Biochemical assays

##### mRNA extraction and cDNA preparation

Cells stored in TRIzol (15596026, Invitrogen) were thawed and resuspended in 1 mL of TRIzol. After adding 200 μL of chloroform, the samples were centrifuged at 12,000 g for 15 minutes at 4°C. The aqueous phase was collected, and RNA was precipitated with GlycoBlue Coprecipitant (AM9515, Invitrogen) and isopropanol. The RNA pellet was washed with 75% ethanol, air-dried, and resuspended in 40 μL of nuclease-free water. The RNA was then isolated using the Zymo Research RNA Clean & Concentrator-5 Kit (R1014, Zymo), quantified with the Qubit RNA HS Assay Kit (Q32852, Invitrogen), and diluted to equalize concentrations. Finally, cDNA libraries were generated using qScript cDNA SuperMix (101414–102, VWR) with a temperature profile of 25°C for 5 minutes, 42°C for 30 minutes, and 85°C for 5 minutes, and the resulting cDNA was diluted threefold and stored at −80°C.

##### Real-time qPCR

cDNA samples were plated on a 96-well qPCR plate and incubated with Fast SYBR Green Master Mix (4385616, Applied Biosystems), nuclease-free water, and the forward and reverse primers of interest at a ratio of 5 μl SYBR: 3 μl water: 0.5 μl forward primer: 0.5 μl reverse primer: 1 μl sample. Each sample was plated two to four times to ensure technical replicates. A control sample (water with primers and Master Mix) served as a negative control. Cycle threshold values were collected for each well and normalized to GAPDH as a housekeeping gene. The sequences of forward (F) and reverse (R) primers used (5′→ 3′) are: Atg7: (F) 5′- GTTCGCCCCCTTTAATAGTGC −3′ and (R) 5′-TGAACTCCAACGTCAAGCGG −3′

##### Protein extraction and Western blotting

Protein was extracted from cultured rat astrocytes using membrane solubilization buffer (25 mM Tris pH 7.4, 150 mM NaCl, 1 mM CaCl_2_, 1 mM MgCl_2_, 0.5% NP-40, and protease inhibitors). Cultured astrocytes were washed twice with ice-cold TBS containing 1 mM CaCl_2_ and 1 mM MgCl_2_ and incubated on ice in membrane solubilization buffer for 20 minutes with occasional agitation. Cell lysates were collected, vortexed briefly, and centrifuged at 4°C at high speed for 10 minutes to pellet non-solubilized material. The supernatant was collected and stored at −80°C.

Pierce BCA Protein Assay Kit (Thermo Fisher) was used to determine protein concentration, and lysates were mixed with 4x Pierce^™^ Reducing Sample Buffer (Thermo Scientific) and incubated at 45°C for 45 minutes to denature proteins. 7–10 μg (cultured astrocyte lysates) of protein was loaded into Bolt^™^ 4–12% Bus-Tris Plus gels (Thermo Scientific) and run at 150 V for 1 hour. Proteins were transferred at 100 V to PVDF membrane (Millipore) for 1.5 hours, blocked in 5% BSA in TBST (137 mM NaCl, 2.68 mM KCl, 24.7 mM Tris-Base, 0.1% (w/v) Tween 20) for 1 hour and incubated in primary antibodies overnight at 4°C. Primary antibodies used were: anti-LRRK2 (Rabbit, 1:500; ab133474, abcam), GAPDH (mouse, 1:5000; ab8245, abcam), β-actin (mouse, 1:5000; A5441, Millipore Sigma). The next day, membranes were washed with TBST, incubated in HRP secondary antibodies (Thermo Fisher Scientific) for 2 hours, washed in TBST, and imaged on a Biorad Gel Doc imaging system. Protein levels were quantified using FIJI.

##### Coimmunoprecipitation

Co-immunoprecipitation assays were performed in HEK293T cells. Cells were transfected 36 hours prior to lysis with Ezrin/Atg7 cDNA using X-tremeGENE HP (Roche) and grown to 85–90% confluency. At 36 hours post-transfection, cells were rinsed with 1x PBS and collected for lysis. Cellular lysis and protein extraction was conducted via brief vortexing in chilled membrane solubilization buffer (25mM HEPES, 150mM KCl, 1.5mM MgCl2, 0.5% NP40, 10% Glycerol) in the presence of both protease (cOmplete, Roche) and phosphatase (PhosSTOP, Roche) inhibitors. Protein concentrations of cell lysates were equalized using the Pierce^™^ BSA Protein Assay Kit (Thermo Fisher) and CLARIOstar Plus Plate Reader (BMG Labtech). Equalized cell lysates were incubated, while rotating, with Pierce^™^ Anti-c-Myc Magnetic Beads (Thermo Fisher) for 6 hours at 4°C. Following this incubation, the beads were washed with chilled lysis buffer. Protein samples were eluted from beads through the addition of 2x Bromophenol Blue-free Laemilli Sample Buffer and heating to 95°C for 5 minutes. Eluted protein samples were subjected to western blot analysis and quantification using a SimpleWestern Jess (ProteinSimple) automated immunoassay system with a 12–230kDa Fluorescence Separation module and associated manufacturer’s protocol. Primary antibodies used for protein detection were: Anti-Myc (Rabbit, 1:40, Cell Signaling Technologies, mAb#2278) and Anti-HA (Rat, 1:20, Roche, 11867423001). Signal detection was achieved using the following fluorescently conjugated secondary antibodies at a dilution of 1:100: IRDye^®^ 680RD Goat anti-Rabbit IgG (LI-COR, 926–68071) and IRDye^®^ 680RD Goat anti-Rat IgG (LI-COR, 926–68076). Signal quantification was conducted with the Simple Western Compass software, utilizing quantitative electropherograms of detected signals. Ezrin co-immunoprecipitation signal intensity was background-subtracted and normalized to Ezrin protein load and Atg7 IP levels for statistical analysis. Statistical analysis was performed in GraphPad Prism 9, using One-way ANOVA with Tukey’s multiple comparisons test and alpha threshold of 0.05.

##### Protein interaction modeling

Full-length mouse Ezrin (UniProt ID: Q4KML7) and Atg7 (UniProt ID: Q9D906) were used for structure prediction in Alphafold 2.0 Multimer. The structure of Ezrin in the open conformation was generated by segmenting independently predicted Ezrin N- and C-termini and threading them onto an open ERM hinge structure using the PyMOL2 molecular visualization software. To model the Ezrin:Atg7 interaction and associated conformational changes, the structures of Ezrin and the Atg7 homodimer were predicted and docked using Alphafold 2.0 Multimer. Conformational changes in the Ezrin:Atg7 protein complex were assessed using PyMOL2 and CABS-flex 2.0^100^. For all predicted structure models, the highest-confidence structures, calculated via the predicted local distance difference test (pLDDT), were subjected to energy-minimization via AMBER relaxation. The resulting minimized structures were imported into PyMOL2 for representative model production.

#### Proteomic analysis

##### *In vivo* BioID protein purification

*In vivo*, BioID experiments were performed as previously described in Takano et al., 2020 with modifications^101^. Genotype-matched animals (wild-type C57BL6 or LRRK2 G2019S^ki/ki^) were bred to produce single-genotype litters. For each genotype (WT or G2019S), 6 pups were injected with AAVs carrying Astro-Ezrin-BioID (PHP.eB.GfaABC1D-Ezrin WT-BioID2-HA) or Astro-CYTO-BioID (PHP.eB.GfaABC1D-BioID2-HA). 2 genotype-matched cortices were pooled at the time of protein isolation, yielding 3 independent replicates per BioID construct. 12 total samples (3 per genotype per construct) were subjected to LC-MS/MS and downstream analysis. WT and LRRK2 G2019S^ki/ki^ P0 – P2 mouse pups were anesthetized by hypothermia, and 1μl of each concentrated AAV-BioID vector was injected bilaterally into the cortex using a Hamilton syringe. Pups were monitored until they recovered on a heating pad. At P18, P19, and P20, biotin was subcutaneously injected at 24 mg/kg to increase the biotinylation efficiency. At P21, the cerebral cortices were removed and stored at −80° C. For the protein purification, each cortex was lysed in a buffer containing 50 mM Tris/HCl, pH 7.5; 150 mM NaCl; 1 mM EDTA; protease inhibitor mixture (Roche); and phosphatase inhibitor mixture (PhosSTOP, Roche). Next, an equal volume of buffer containing 50 mM Tris/HCl, pH 7.5; 150 mM NaCl; 1 mM EDTA; 0.4 % SDS; 2 % TritonX-100; 2 % deoxycholate; protease inhibitor mixture; and phosphatase inhibitor mixture was added to the samples, following by sonication and centrifugation at 15,000 g for 10 min. The remaining supernatant was ultracentrifuged at 100,000g for 30min at 4° C. Finally, SDS detergent was added to the samples and heated at 45 ° C for 45 min. After cooling on ice, each sample was incubated with High-Capacity Streptavidin Agarose beads (ThermoFisher) at 4° C overnight. Following incubation, the beads were serially washed: 1) twice with a solution containing 2% SDS; 2) twice with a buffer 1% TritonX-100, 1% deoxycholate, 25 mM LiCl; 3) twice with 1M NaCl, and finally, five times with 50 mM ammonium bicarbonate. The biotinylated proteins attached to the agarose beads were eluted in a buffer of 125 mM Tris/HCl, pH6.8; 4 % SDS; 0.2 % β-mercaptoethanol; 20 % glycerol; 3 mM biotin at 60°C for 15min.

##### Sample Preparation

The Duke Proteomics and Metabolomics Shared Resource (DPMSR) received 12 samples (3 of each WT CYT, GS CYT, WT EZR, and GS EZR) which were kept at −80C until processing. Samples were spiked with undigested bovine casein at a total of either 1 or 2 pmol as an internal quality control standard. Next, they were reduced for 15 min at 80C, alkylated with 20 mM iodoacetamide for 30 min at room temperature, then supplemented with a final concentration of 1.2% phosphoric acid and 636 μl of S-Trap (Protifi) binding buffer (90% MeOH/100mM TEAB). Proteins were trapped on the S-Trap micro cartridge, digested using 20 ng/μL sequencing grade trypsin (Promega) for 1 hr at 47C, and eluted using 50 mM TEAB, followed by 0.2% FA, and lastly using 50% ACN/0.2% FA. All samples were then lyophilized to dryness. Samples were resuspended in 12 μl of 1% TFA/2% acetonitrile with 12.5 fmol/μl of yeast ADH. A study pool QC (SPQC) was created by combining equal volumes of each sample.

##### LC-MS/MS Analysis

Quantitative LC/MS/MS was performed on 3 μL of each sample, using an MClass UPLC system (Waters Corp) coupled to a Thermo Orbitrap Fusion Lumos high resolution accurate mass tandem mass spectrometer (Thermo) equipped with a FAIMSPro device via a nanoelectrospray ionization source. Briefly, the sample was first trapped on a Symmetry C18 20 mm × 180 μm trapping column (5 μl/min at 99.9/0.1 v/v water/acetonitrile), after which the analytical separation was performed using a 1.8 μm Acquity HSS T3 C18 75 μm × 250 mm column (Waters Corp.) with a 90-min linear gradient of 5 to 30% acetonitrile with 0.1% formic acid at a flow rate of 400 nanoliters/minute (nL/min) with a column temperature of 55C. Data collection on the Fusion Lumos mass spectrometer was performed for three difference compensation voltages (−40v, −60v, −80v). Within each CV, a data-dependent acquisition (DDA) mode of acquisition with a r=120,000 (@ m/z 200) full MS scan from m/z 375 – 1500 with a target AGC value of 4e5 ions was performed. MS/MS scans with HCD settings of 30% were acquired in the linear ion trap in “rapid” mode with a target AGC value of 1e4 and max fill time of 35 ms. The total cycle time for each CV was 0.66s, with total cycle times of 2 sec between like full MS scans. A 20s dynamic exclusion was employed to increase the depth of coverage. The total analysis cycle time for each sample injection was approximately 2 hours.

##### Quantitative Data Analysis

Following 15 total UPLC-MS/MS analyses (excluding conditioning runs, but including 3 replicate SPQC samples), data were imported into Proteome Discoverer 3.0 (Thermo Scientific Inc.) and individual LCMS data files were aligned based on the accurate mass and retention time of detected precursor ions (“features”) using Minora Feature Detector algorithm in Proteome Discoverer. Relative peptide abundance was measured based on peak intensities of selected ion chromatograms of the aligned features across all runs. The MS/MS data was searched against the SwissProt *M. musculus* database, a common contaminant/spiked protein database (bovine albumin, bovine casein, yeast ADH, etc.), and an equal number of reversed-sequence “decoys” for false discovery rate determination. Sequest with INFERYS was utilized to produce fragment ion spectra and to perform the database searches. Database search parameters included fixed modification on Cys (carbamidomethyl) and variable modification on Met (oxidation). Search tolerances were 2ppm precursor and 0.8Da product ion with full trypsin enzyme rules. Peptide Validator and Protein FDR Validator nodes in Proteome Discoverer were used to annotate the data at a maximum 1% protein false discovery rate based on q-value calculations. Note that peptide homology was addressed using razor rules in which a peptide matched to multiple different proteins was exclusively assigned to the protein has more identified peptides. Protein homology was addressed by grouping proteins that had the same set of peptides to account for their identification. A master protein within a group was assigned based on % coverage.

Prior to normalization, a filter was applied such that a peptide was removed if it was not measured at least twice across all samples and in at least 50% of the replicates in any one single group. After that filter, samples were total intensity normalized (total intensity of all peptides for a sample are summed and then normalized across all samples). Next, the following imputation strategy is applied to missing values. If less than half of the values are missing in a biological group, values are imputed with an intensity derived from a normal distribution of all values defined by measured values within the same intensity range (20 bins). If greater than half values are missing for a peptide in a group and a peptide intensity is > 5e6, then it was concluded that the peptide was misaligned and its measured intensity is set to 0. All remaining missing values are imputed with the lowest 2% of all detected values. Peptide intensities were then subjected to a trimmed-mean normalization in which the top and bottom 10 percent of the signals were excluded, and the average of the remaining values was used to normalize across all samples. Lastly, all peptides belonging to the same protein were then summed into a single intensity. These normalized protein level intensities are what was used for the remainder of the analysis.

To assess technical reproducibility, we calculated the % coefficient of variation (%CV) for each protein across 3 injections of an SPQC pool that were interspersed throughout the study (Table S4). The mean %CV of the SPQC pools was 11.7%, which is well within our expected analytical tolerances. To assess biological + technical variability, %CVs were measured for each protein across the individual groups which averaged 19.8%, which is also within our normal tolerances and indicated a reproducible IP and sample processing.

As an initial statistical analysis, we calculated fold-changes between various sample groups based on the protein expression values and calculated two-tailed heteroscedastic t-test on log2-transformed data for this comparison. Those fold changes and p-values are presented for all proteins in Tables S5, S7, S9, and S10. False Discovery Rate (FDR) correction was performed using the Benjamini-Hochberg procedure, and these values are reported (“p.adjusted”) in Tables S5, S7, S9, and S10. To optimize for discovery, we opted to use the less stringent parameter of unadjusted p-value for downstream analyses. Within supplemental tables, we have labeled proteins significantly more abundant (“Up”, fold change >1.5 and unadjusted p-value <0.05), or less abundant (“Down”, fold change >−1.5 and unadjusted p-value <0.05) in a particular genotype or BioID sample group. These annotated proteins were used for downstream analyses, including protein interaction networks using Cytoscape (v.3.9.1) and Gene Ontology (GO) enrichment analysis using the ClusterProfiler package for R, with all M. musculus genes as the reference background.

##### Mouse synapse imaging and analysis

Staining, image acquisition, and analysis of synaptic density were performed as described previously^102^. Synaptic staining was performed in coronal sections (30 μm thick) containing the ACC and MOp from WT and LRRK2 G2019S^ki/ki^ mice. To label pre and postsynaptic proteins, the following antibody combinations were used: VGluT1 and PSD95 (excitatory, intracortical), VGAT, and GEPHYRIN (inhibitory). High magnification 60x objective plus 1.64x optical zoom z-stack images containing 15 optical sections spaced 0.34 μm apart were obtained using an Olympus FV 3000 inverted confocal microscope. Each z-stack was converted into 5 maximum projection images (MPI), each corresponding to a 1 μm section of z plane, using FIJI. Synapses were identified by the colocalization of pre and postsynaptic puncta. The number of co-localized synaptic puncta of excitatory intracortical (VGluT1-PSD95) and inhibitory (VGAT-GEPHYRIN) synapses were obtained using the FIJI plugin Puncta Analyzer^47^. 15 MPIs were analyzed for each mouse (5 MPI/tissue section, 3 tissue sections/mouse). Between 4 and 5, age and sex-matched mice/genotype/condition were analyzed for each synaptic staining combination, as indicated in the figure legends. All animals appeared healthy at the time of collection. No data were excluded.

##### Cell counting, imaging, and analysis

Tile scan images containing the anterior cingulated cortex (ACC) and primary motor cortex (MOp) from P21 WT and LRRK2 G2019S^ki/ki^; Aldh1l1-eGFP mice were acquired on a confocal Leica SP8 STED microscope using the galvo scanner and 20x objective. For ALDH1L1-EGFP and SOX9 double positive cell counting in the ACC and MOp, the cells labeled with ALDH1L1-EGFP and SOX9 were counted by hand using the cell counter tool in FIJI. 2–4 sections per brain from 3 sex and age-matched mice/genotype were analyzed.

##### Human brain section staining

Floating human frontal cortex sections of 40 μm thickness were obtained from Banner Sun Health Research Institute in Sun City, Arizona (4 control and 3 LRRK2 G2019S mutation carrier subjects). None of the control subjects had a history of dementia or neurological or psychiatric disorders at the time of death (See Supplemental Table 1). Informed and written consent was obtained from the donors. For immunostaining, sections were washed in 1x TBS containing 0.3% Triton X-100 (TBST), blocked in 3% NGS diluted in TBST, and incubated in primary antibody 2–3 nights at 4°C with shaking. Primary antibodies used were GFAP (chicken, 1:250; AB5541, Millipore Sigma) and phospho-ERM (Rabbit, 1:250; #3726, Cell Signaling). Following primary incubation, sections were washed in TBST, incubated in Alexa Fluor conjugated secondary antibodies diluted 1:200 (Life Technologies) for 2–3 hours at room temperature, washed with TBST, and mounted onto glass slides using a homemade mounting media (90% Glycerol, 20 mM Tris pH 8.0, 0.5% n-Propyl gallate) and sealed with nail polish.

##### Mouse brain slice electrophysiology

For whole-cell patch-clamp recordings, 3–4 mice of each genotype and condition were used for miniature excitatory postsynaptic current (mEPSC) and miniature inhibitory postsynaptic current (mIPSC) measurements. WT and *LRRK2* G2019S^ki/ki^ mice of both sexes were anesthetized with 200 mg/kg tribromoethanol (avertin) and decapitated. After decapitation, the brains were immersed in ice-cold artificial cerebrospinal fluid (aCSF, in mM): 125 NaCl, 2.5 KCl, 3 mM MgCl_2_, 0.1 mM CaCl_2_, 10 glucose, 25 NaHCO_3_, 1.25 NaHPO_4_, 0.4 L-ascorbic acid, and 2 Na-pyruvate, pH 7.3–7.4 (310 mOsmol). 350 μm thick coronal slices containing the ACC were obtained using a vibrating tissue slicer (Leica VT1200; Leica Biosystems). Slices were immediately transferred to standard aCSF (33°C, continuously bubbled with 95% O_2_ – 5% CO_2_) containing the same as the low-calcium aCSF but with 1 mM MgCl_2_ and 1–2 mM CaCl_2_. After 30-minute incubation at 33°C, slices were transferred to a holding chamber with the same extracellular buffer at room temperature (RT: ~25°C). Brain slices were visualized by an upright microscope (BX61WI, Olympus) through a 40x water-immersion objective equipped with infrared-differential interference contrast optics in combination with a digital camera (ODA-IR2000WCTRL). Patch-clamp recordings were performed by using an EPC 10 patch-clamp amplifier, controlled by Patchmaster Software (HEKA). Data were acquired at a sampling rate of 50 kHz and low-pass filtered at 6 kHz.

To measure mEPSCs, the internal solution contained the following (in mM): 125 K-gluconate, 10 NaCl, 10 HEPES, 0.2 EGTA, 4.5 MgATP, 0.3 NaGTP, and 10 Na-phosphocreatine, pH adjusted to 7.2 – 7.4 with KOH and osmolality set to ~ 300 mOsmol. mEPSCs were measured in the aCSF bath solution containing 1 μM tetrodotoxin and 50 μM Picrotoxin at −70 mV in voltage-clamp mode. To measure mIPSCs, the internal solution contained the following (in mM): 77 K-gluconate, 77 KCl, 10 HEPES, 0.2 EGTA, 4.5 MgATP, 0.3 NaGTP, and 10 Na-phosphocreatine, pH adjusted to 7.2 – 7.4 with KOH and osmolality set to ~ 300 mOsmol. mIPSCs were measured in the aCSF bath solution containing 1 μM tetrodotoxin and 10 μM 6-cyano-7-nitroquinoxaline-2,3-dione (CNQX), and 50 μM D-2-amino-5-phosphonopentanoate (D-AP5) at −70 mV in voltage-clamp mode. mEPSCs and mIPSCs recorded at −70 mV were detected using Minhee Analysis software (https://github.com/parkgilbong/Minhee_Analysis_Pack). To analyze the frequency, events were counted over 5 minutes of recording. To obtain the average events for each cell, at least 100 non-overlapping events were detected and averaged. The peak amplitude of the average mEPSCs was measured relative to the baseline current.

##### Quantification and statistical analysis

All statistical analyses were performed in GraphPad Prism 9. Exact value of n, what n represents, and specific statistical tests for each experiment are indicated in the figure legend for each experiment. All data are represented as mean ± standard error of the mean, and data points are shown where applicable. Exact *P*-values are listed in the figure for each experiment. Where indicated, the unpaired two-tailed t-tests were run using Welch`s correction, and a correction for multiple comparisons was applied using Hom-Sidak method with an alpha threshold of 0.05 for adjusted *P*-value. A Geisser-Greenhouse correction was used for both One-way and Two-way ANOVA analyses. For Sholl analysis, statistical analyses were conducted using a custom code in R (https://github.com/Eroglu-Lab/In-Vitro-Sholl). A mixed-effect model with multiple comparisons made using the Tukey post-test was used for Sholl analysis to account for the variability per experiment as a random effect to evaluate differences between the conditions. Sample sizes were determined based on previous experience for each experiment to yield high power to detect specific effects. No statistical methods were used to predetermine the sample size. All experimental animals that appeared healthy at the time of tissue collection were processed for data collection.

## Supplementary Material

Supplement 1

## Figures and Tables

**Table:** KEY RESOURCES TABLE

REAGENT or RESOURCE	SOURCE	IDENTIFIER
β-actin	Sigma-Aldrich	Cat# A5441, RRID: AB_476744
ERM	Cell Signaling	Cat# 3142, RRID:AB_2100313
GAPDH	Abcam	Cat# ab8245, RRID:AB_2107448
Gephyrin	Synaptic Systems	Cat# 147 011, RRID:AB_887717
GFAP	Agilent	Cat# Z0334, RRID:AB_10013382
GFAP	Millipore	Cat# AB5541, RRID:AB_177521
GFP	Aves labs	GFP-1020, RRID:AB_10000240
HA	Roche	Cat# 11867423001, RRID:AB_390918
L1 (ASCS4)	DSHB	Cat# ascs4, RRID:AB_528349
LRRK2	Abcam	Cat# ab133474, RRID:AB_2713963
phospho-ERM	Cell Signaling	Cat# 3141, RRID:AB_330232
phospho-ERM	Cell Signaling	Cat# 3726, RRID: AB_10560513
phospho-Rab10	Abcam	Cat# ab230261, RRID:AB_2811274
PSD-95	Innovative Research	Cat# 51-6900, RRID: AB_87705
Sox9	Millipore	Cat# AB5535, RRID:AB_2239761
VGAT	Synaptic Systems	Cat# 131 004, RRID: AB_887873
VGluT1	Synaptic Systems	Cat# 135304, RRID: AB_887878
**Bacterial and virus strains**		
One Shot STBL3	Thermo Fisher	Cat# C737303
AAV PhP.eB	This paper	N/A
**Chemicals, peptides, and recombinant proteins**		
AraC	Sigma	Cat# C1768
B27	GIBCO	Cat# 17504044
B27 Plus	GIBCO	Cat# A3582801
BDNF	PeproTech	Cat# 450-02
Carboxypeptidase E	R&D	Cat# 3587-ZN
CNTF	PeproTech	Cat# 450-13
DMEM	GIBCO	Cat# 11960
DPBS	GIBCO	Cat# 14287
Dystroglycan	R&D	Cat# 6868-DG
Fetal Bovine Serum	Thermo Fisher	Cat# 10-437-028
Forskolin	Sigma	Cat# F6886
Glycerol	Acros Organics	Cat# AC158920010
L-Glutamine	GIBCO	Cat# 25030-081
Hydrocortisone	Sigma	Cat# H0888-5G
Insulin	Sigma	Cat# I1882
Neurobasal	GIBCO	Cat# 21103049
Neurobasal Plus	GIBCO	Cat# A3582901
Odyssey Blocking Buffer	LI-COR Biosciences	Cat# 927-40000
Opti-MEM	Thermo Fisher	Cat# 11058021
Paraformaldehyde	Electron microscopy Sciences	Cat# 19210
Pen/Strep	GIBCO	Cat# 15140
PhosSTOP^™^	Roche	Cat# 4906845001
Poly-D-Lysine	Sigma	Cat# P6407
Protease Inhibitor Cocktail	Roche	Cat# 4693132001
RIPA Buffer	Sigma	Cat# R0278
Sodium Pyruvate	GIBCO	Cat# 11360-070
Tissue-Tek^®^ O.C.T. Compound	Sakura^®^ Finetek	Cat# 4583
Triton X-100	Roche	Cat# 11332481001
**Critical commercial assays**		
Endo-Free Maxi Prep Kit	QIAGEN	Cat# 12362
**Experimental models: cell lines**		
HEK293T	ATCC	CRL-11268
Rat primary cortical neurons	This paper	N/A
Rat primary cortical astrocytes	This paper	N/A
**Experimental models: organisms/strains**		
Mouse: Aldh1L1-EGFP	MMRRC	RRID: MMRRC_011015-UCD
Mouse: CD1	Charles River	RRID: IMSR_CRL:022
Mouse: LRRK2 G2019S^ki/ki^	Taconic	RRID: IMSR_TAC:13940
Rat: Sprague-Dawley	Charles River	001
**Recombinant DNA**		
pZac2.1-GfaABC1D-Ezrin WT-BioID2-HA	This paper	N/A
pZac2.1-GfaABC1D-Ezrin T567D-BioID2-HA	This paper	N/A
pZac2.1-GfaABC1D-Ezrin T567A-BioID2-HA plasmid	This paper	N/A
pLKO.1-sh*Atg7*-EGFP	This paper	N/A
pLKO.1-shControl-EGFP	This paper	N/A
pPB-CAG-sh*LRRK2*-mCherry-CAAX	This paper	N/A
pPB-CAG-sh*Atg7*-mCherry-CAAX	This paper	N/A
pPB-CAG-shControl-mCherry-CAAX	This paper	N/A
**Software and algorithms**		
GraphPad Prism 9	GraphPAD	https://www.graphpad.com/scientificsoftware/prism/
ImageJ	NIH	https://imagej.nih.gov/ij/
Puncta Analyzer	Savage et al., 2024^91^	https://github.com/Eroglu-Lab/Syn_Bot
Minhee Analysis	Kim et al., 2021^92^	https://github.com/parkgilbong/Minhee_Analysis_Pack
In vitro Sholl analysis	Tan et al., 2023^93^	https://github.com/Eroglu-Lab/In-Vitro-Sholl
Image Studio	LICOR	https://www.licor.com

## Data Availability

The data that support the findings of this study are available from the corresponding author (Dr. Cagla Eroglu) upon request after the publication of this manuscript. The data include experiment records, raw data, and analysis. The authors confirm that all data underlying the findings will be made fully available without restriction upon publication.
