# Supplementary material for: PD-linked LRRK2 G2019S mutation impairs astrocyte morphology and synapse maintenance via ERM hyperphosphorylation
Source: bioRxiv. 2025 Apr 25:2023.04.09.536178. Preprint. [Version 4] doi: 10.1101/2023.04.09.536178 (PMC11383028; doi:10.1101/2023.04.09.536178)
Supplement: Supplement 2 [file NIHPP2023.04.09.536178v4-supplement-2.pdf]

bioRxiv preprint doi: https://doi.org/10.1101/2023.04.09.536178; this version posted April 25, 2025. The copyright holder for this preprint (which was not certified by peer review) is the author/funder, who has granted bioRxiv a license to display the preprint in perpetuity. It is made available under aCC-BY 4.0 International license.

Supplemental information

PD-linked LRRK2 G2019S mutation impairs astrocyte morphology and synapse maintenance via ERM hyperphosphorylation

Shiyi Wang, Ryan Baumert, Gabrielle Séjourné, Dhanesh Sivadasan Bindu, Kylie Dimond, Kristina Sakers, Leslie Vazquez, Jessica Moore, Christabel Xin Tan, Tetsuya Takano, Maria Pia Rodriguez, Nick Brose, Luke Bradley, Reed Lessing, Scott H. Soderling, Albert R. La Spada, and Cagla Eroglu

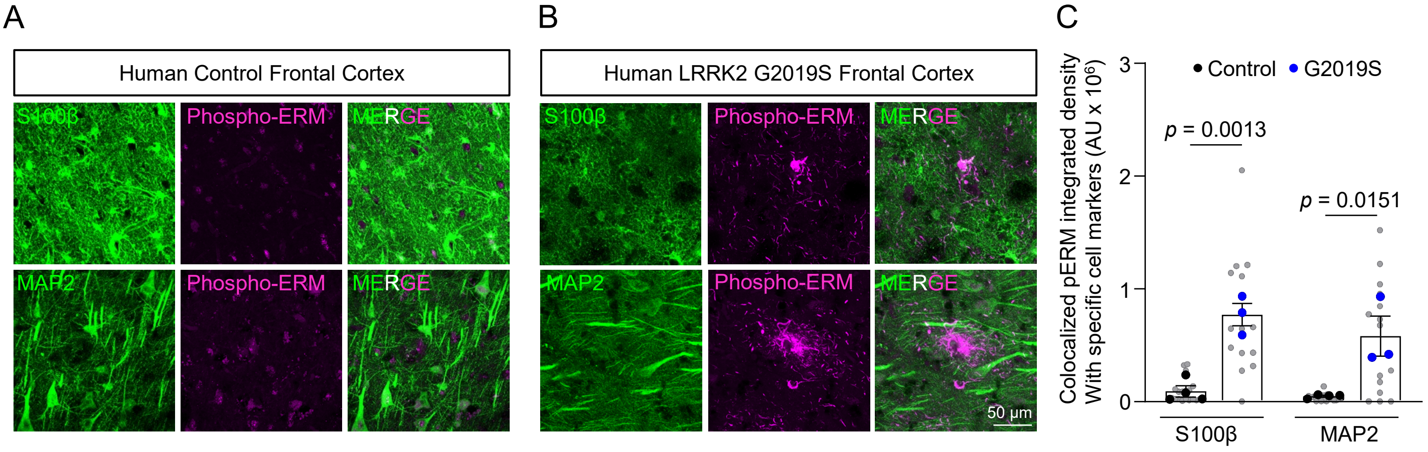

Figure S1: Human phospho-ERM is significantly increased and colocalizes with S100β and MAP2. Related to Figure 1.

(A-B) Representative confocal images of phospho-ERM (purple) and S100β (green) or MAP2 (green) in the frontal cortex of human control subjects or human PD patients carrying LRRK2 G2019S mutation carriers at age >80 years old. Scale bar, 50 µm. **(C)** Quantification of phospho-ERM integrated density in (A-B), n = 4 (Human control, 3 males and 1 female), 3 (LRRK2 G2019S mutation carriers, 2 males and 1 female) subjects, nested t-test, unpaired two-tailed t-test. For colocalized phospho-ERM with S100β, t (5) = 6.541, p = 0.0013. For colocalized phospho-ERM with MAP2, t (5) = 3.625, p = 0.0151. Grey dots are the data acquired from each image. Black dots are the averaged data acquired from each control subject. Blue dots are the averaged data acquired from each LRRK2 G2019S mutation carrier.

Figure S2 (related to Figure 1)

Wang et al.,

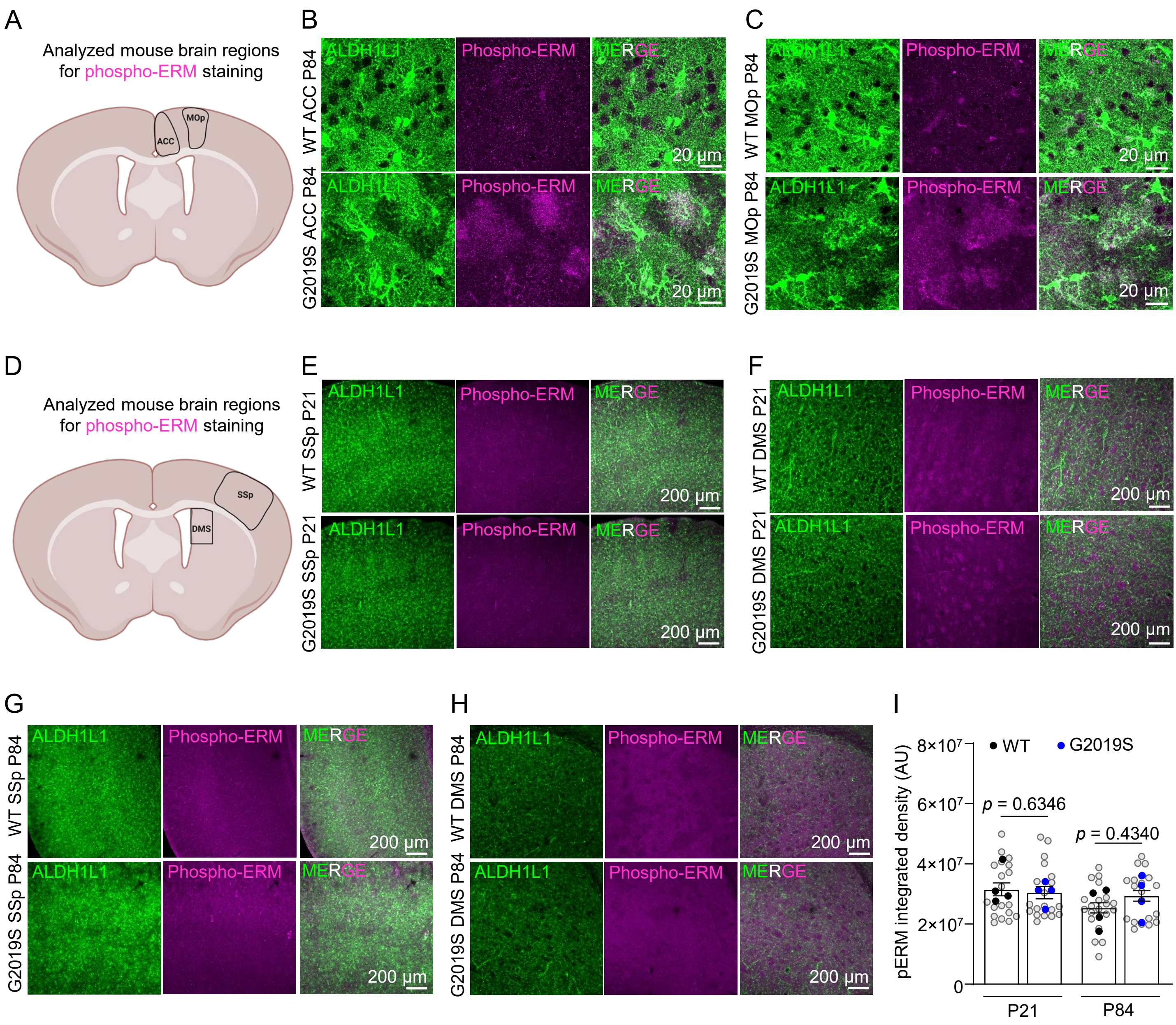

Figure S2: Phospho-ERM is upregulated in LRRK2 G2019S$^{ki/ki}$ astrocytes in ACC and MOp but not SSp and DMS. Related to Figure 1.

(A) Schematic representation of analyzed mouse brain regions for phospho-ERM staining, the ACC, and the MOp. **(B-C)** Representative confocal images of ERM phosphorylation (purple) in the ACC and MOp of WT or LRRK2 G2019S$^{ki/ki}$ Aldh1L1-eGFP mice at P84. Scale bar, 20 µm. **(D)** Schematic representation of analyzed mouse brain regions for phospho-ERM staining, the somatosensory cortex (SSp), and the dorsal medial striatum (DMS). **(E-H)** Representative confocal images of ERM phosphorylation (purple) in the SSp and DMS of WT or LRRK2 G2019S$^{ki/ki}$ Aldh1L1-eGFP mice at P21 (E-F) and at P84 (G-H). Scale bar, 200 µm. **(I)** Quantification of phospho-ERM integrated density in (E-H). For phospho-ERM integrated density at P21, nested t-test, unpaired two-tailed t-test. t (6) = 0.5005, p = 0.6346. n = 4 (WT, 2 males and 2 females), 4 (LRRK2 G2019S$^{ki/ki}$, 2 males and 2 females) mice. For phospho-ERM integrated density at P84, nested t-test, unpaired two-tailed t-test. t (6) = 0.8383, p = 0.1123. n = 4 (WT, 2 males and 2 females), 4 (LRRK2 G2019S$^{ki/ki}$, 2 males and 2 females) mice.

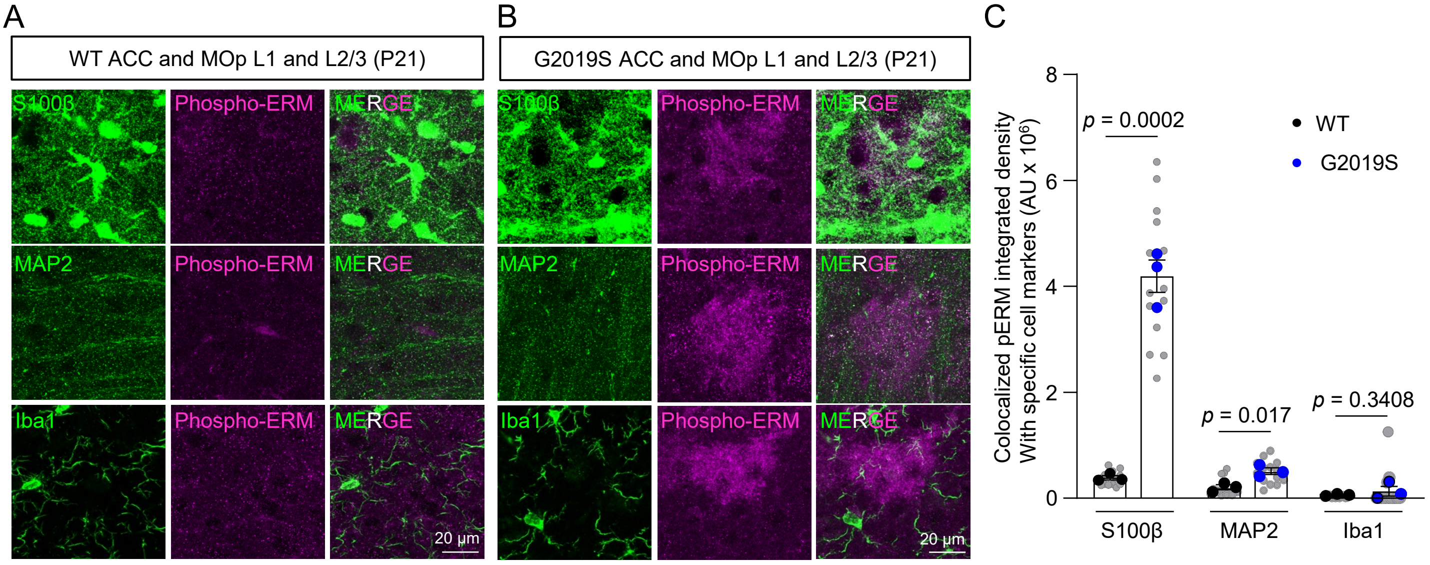

Figure S3: mouse phospho-ERM is significantly increased and colocalizes with S100β and MAP2. Related to Figure 1.

(A-B) Representative confocal images of phospho-ERM (purple) and S100β (green), MAP2 (green), or Iba1 (green) from the ACC and MOp of WT or LRRK2 G2019S$^{ki/ki}$ mice at P21. Scale bar, 20 μm. **(C)** Quantification of colocalized phospho-ERM integrated density with S100β, MAP2, and Iba1 in (D-E), n = 3 (WT, 1 male and 2 females), 3 (LRRK2 G2019S$^{ki/ki}$, 1 male and 2 females) mice, nested t-test, unpaired two-tailed t-test. For colocalized phospho-ERM with S100β, t (4) = 12.37, *p* = 0.0002. For colocalized phospho-ERM with MAP2, t (4) = 3.939, *p* = 0.017. For colocalized phospho-ERM with Iba1, t (4) = 1.08, *p* = 0.3408. Grey dots are the data acquired from each image. Black dots are the averaged data acquired from each WT mouse. Blue dots are the averaged data acquired from each LRRK2 G2019S$^{ki/ki}$ mouse.

Figure S4 (related to Figure 1) **Wang et al.,**

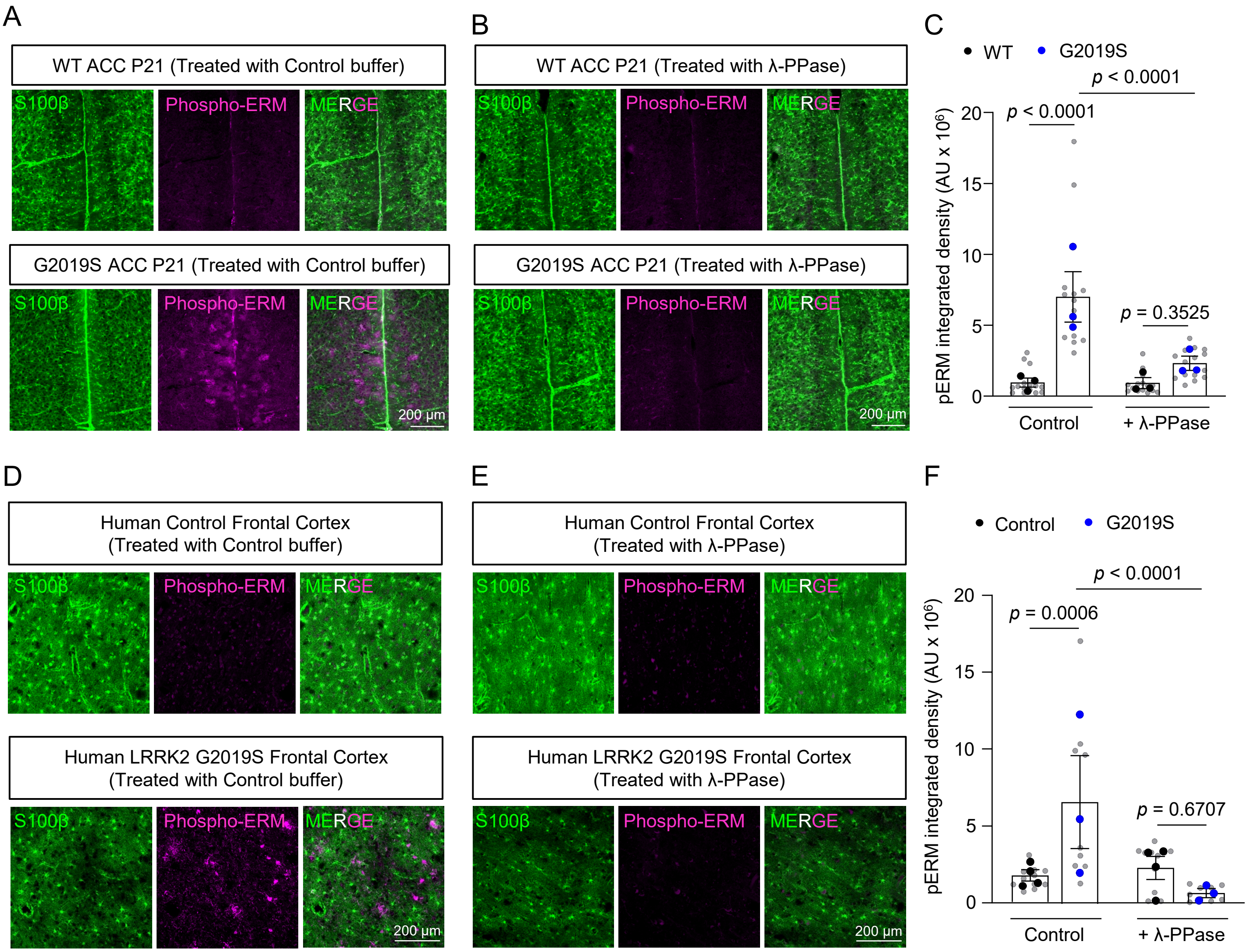

Figure S4: phospho-ERM is eliminated after Lambda protein phosphatase (λ-PPase) treatment. Related to Figure 1.

(A-B) Representative confocal images of S100β (green) and phospho-ERM (purple) in the ACC of WT or LRRK2 G2019S$^{ki/ki}$ mice at P21 with or without λ-PPase treatment. Scale bar, 200 µm. **(C)** Quantification of phospho-ERM integrated density in (A-B), n = 3 (WT, 1 male and 2 females), 3 (LRRK2 G2019S$^{ki/ki}$, 1 male and 2 females) mice, Nested One-way ANOVA [F (3, 56) = 25.19, p < 0.0001], Bonferroni's multiple comparisons test revealed a significant difference between WT mice and LRRK2 G2019S$^{ki/ki}$ mice without λ-PPase treatment (p < 0.0001, 95% C.I. = [-8107799, -3924949]), and between LRRK2 G2019S$^{ki/ki}$ mice with and without λ-PPase treatment (p < 0.0001, 95% C.I. = [2605448, 6788298]). alpha = 0.05. Grey dots are the data acquired from each image. Black dots are the averaged data acquired from each WT mouse. Blue dots are the averaged data acquired from each LRRK2 G2019S$^{ki/ki}$ mouse. **(D-E)** Representative confocal images of S100β (green) and phospho-ERM (purple) in the frontal cortex of human control subjects or human PD patients carrying LRRK2 G2019S mutation carriers at age >80 years old with or without λ-PPase treatment. Scale bar, 200 µm. **(F)** Quantification of phospho-ERM integrated density in (D-E), n = 4 (Human control, 3 males and 1 female), 3 (LRRK2 G2019S mutation carriers, 2 males and 1 female) subjects, Nested One-way ANOVA [F (3, 38) = 9.296, p < 0.0001], Bonferroni's multiple comparisons test revealed a significant difference between human control subjects and human PD patients carrying LRRK2 G2019S mutation carriers without λ-PPase treatment (p = 0.0006, 95% C.I. = [-7928018, -1867234]), and between human PD patients carrying LRRK2 G2019S mutation carriers with and without λ-PPase treatment (p < 0.0001, 95% C.I. = [2768186, 9247437]). alpha = 0.05. Grey dots are the data acquired from each image. Black dots are the averaged data acquired from each control subject. Blue dots are the averaged data acquired from each LRRK2 G2019S mutation carrier.

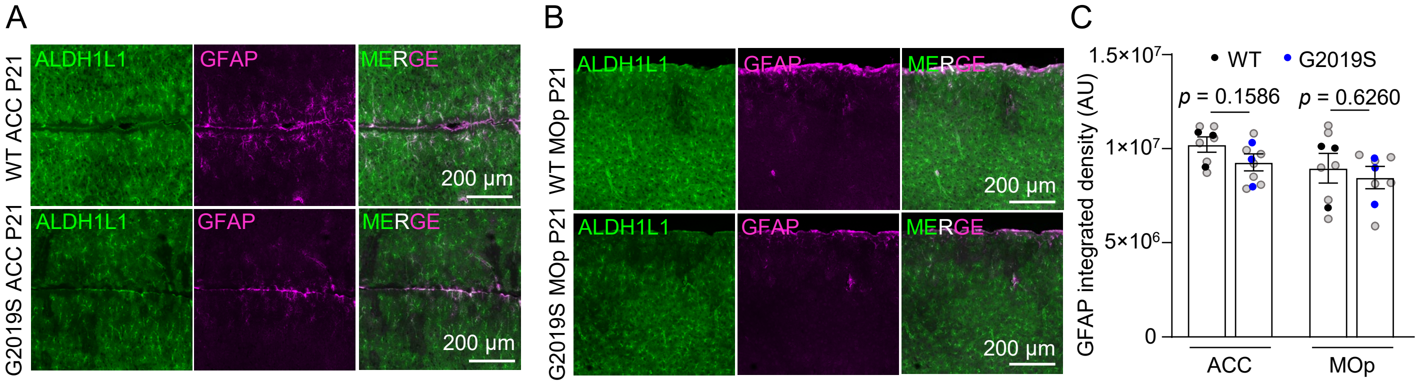

Figure S5: GFAP level is not altered in LRRK2 G2019S$^{ki/ki}$ ACC and MOp. Related to Figure 1.

(A-B) Representative confocal images of GFAP (purple) in the ACC and MOp of WT or LRRK2 G2019S$^{ki/ki}$ Aldh1l1-eGFP mice at P21. Scale bar, 200 µm. **(C)** Quantification of GFAP integrated density in (A-B), n = 3 (WT, 2 males and 1 female), 3 (LRRK2 G2019S$^{ki/ki}$, 2 males and 1 female) mice, For GFAP quantification in the ACC, nested t-test, unpaired two-tailed t-test. t (4) = 1.023, *p* = 0.364. For GFAP quantification in the MOp, nested t-test, unpaired two-tailed t-test. t (4) = 0.375, *p* = 0.7267. Grey dots are the data acquired from each image. Black dots are the averaged data acquired from each WT mouse. Blue dots are the averaged data acquired from each LRRK2 G2019S$^{ki/ki}$ mouse.

Figure S6 (related to Figure 2) **Wang et al.,**

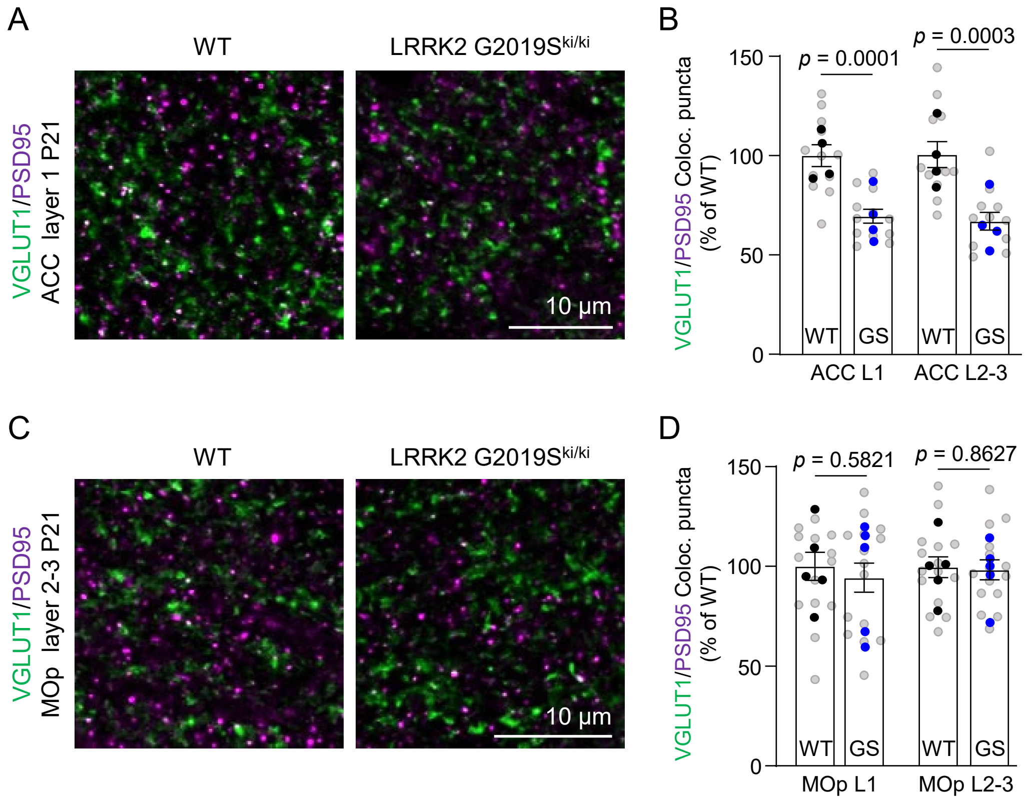

Figure S6: LRRK2 G2019S does not change excitatory synapse numbers in the primary motor cortex. Related to Figure 2.

(A) Representative images from the ventral ACC of WT and LRRK2 G2019S$^{ki/ki}$ mice that were stained with VGluT1 and PSD95 antibodies at P21. Scale bar, 10 μm. **(B)** Quantification of VGluT1-PSD95 co-localized puncta, normalized using the means of WT values in the ventral ACC. n = 4 (WT, 2 males and 2 females), 4 (LRRK2 G2019S$^{ki/ki}$, 2 males and 2 females) mice. Nested One-way ANOVA [F (3, 44) = 12.98, $p < 0.0001$], Bonferroni's multiple comparisons test revealed a significant difference between WT ACC L1 and LRRK2 G2019S$^{ki/ki}$ ACC L1 (p = 0.0002, 95% C.I. = [13.71, 47.33]), and between WT ACC L2-3 and LRRK2 G2019S$^{ki/ki}$ ACC L2-3 ($p < 0.0001$, 95% C.I. = [16.48, 50.10]). alpha = 0.05. **(C)** Representative images from the MOp of WT and LRRK2 G2019S$^{ki/ki}$ mice that were stained with VGluT1 and PSD95 antibodies at P21. Scale bar, 10 μm. **(D)** Quantification of VGluT1-PSD95 co-localized puncta, normalized using the means of WT values in the MOp L1 and L2-3. n = 5 (WT), 5 (LRRK2 G2019S$^{ki/ki}$) mice, 3 males and 2 females. Nested One-way ANOVA [F (3, 56) = 0.1837, p = 0.9070], Bonferroni's multiple comparisons test revealed a significant difference between WT MOp L1 and LRRK2 G2019S$^{ki/ki}$ MOp L1 ($p > 0.9999$, 95% C.I. = [-14.69, 25.97]), and between WT MOp L2-3 and LRRK2 G2019S$^{ki/ki}$ MOp L2-3 ($p > 0.9999$, 95% C.I. = [-19.06, 21.61]). alpha = 0.05. Grey dots are the data acquired from each image. Black dots are the averaged data acquired from each WT mouse. Blue dots are the averaged data acquired from each LRRK2 G2019S$^{ki/ki}$ mouse.

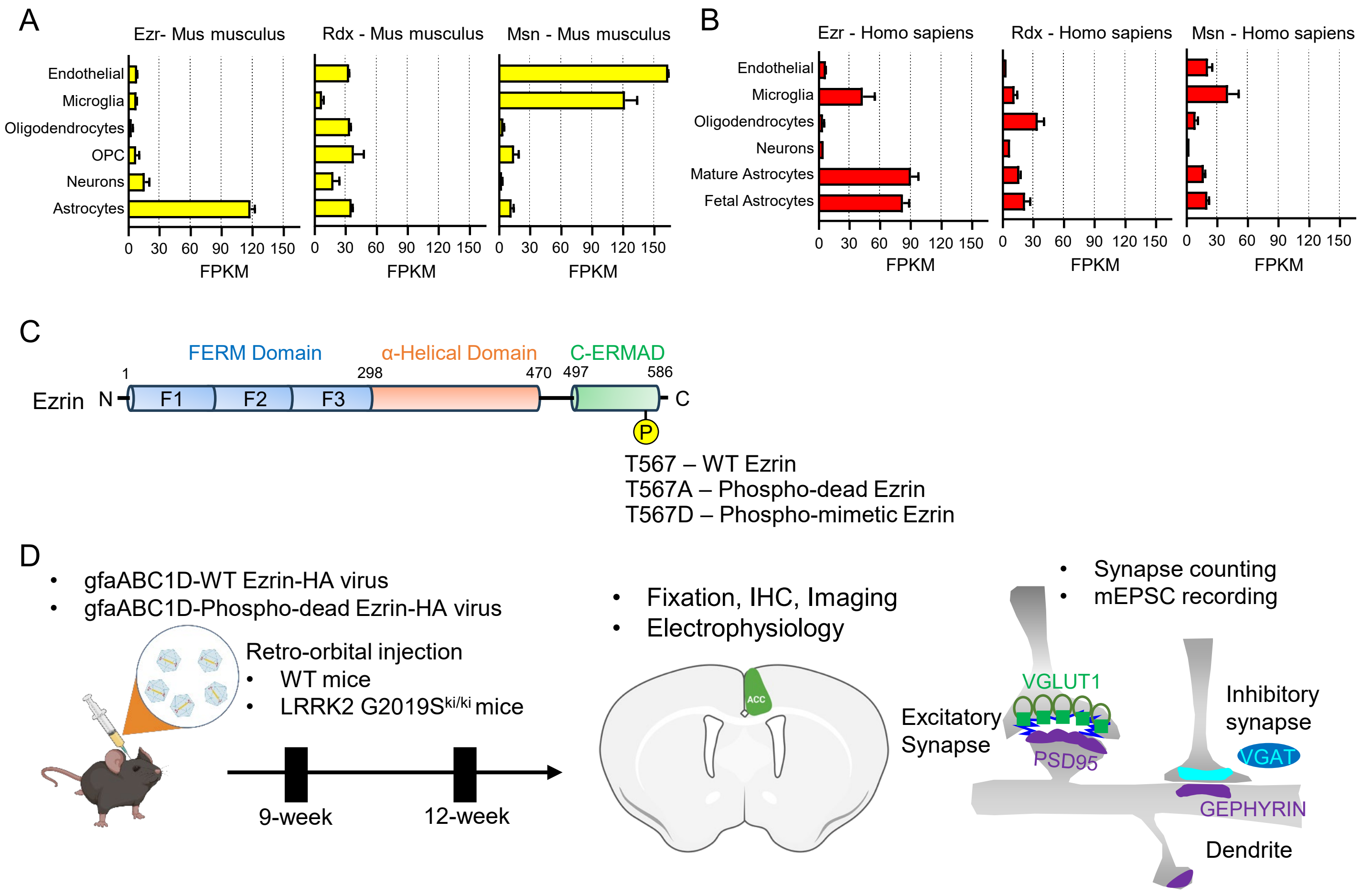

Figure S7: Overexpression of AAV-HA-tagged-WT Ezrin and AAV-HA-tagged-Phospho-dead Ezrin in WT and LRRK2 G2019S$^{ki/ki}$ astrocytes. Related to Figure 4.

(A-B) Bar plots of Ezr, Rdx, and Msn gene expression in various brain cell types in mice (yellow) or humans (red). These plots are generated using publicly available data at https://www.brainrnaseq.org **(C)** Schematic of domains within Ezrin. **(D)** Experiment workflow of AAV-HA-tagged-WT Ezrin and AAV-HA-tagged-Phospho-dead Ezrin in WT and LRRK2 G2019S$^{ki/ki}$ astrocytes.

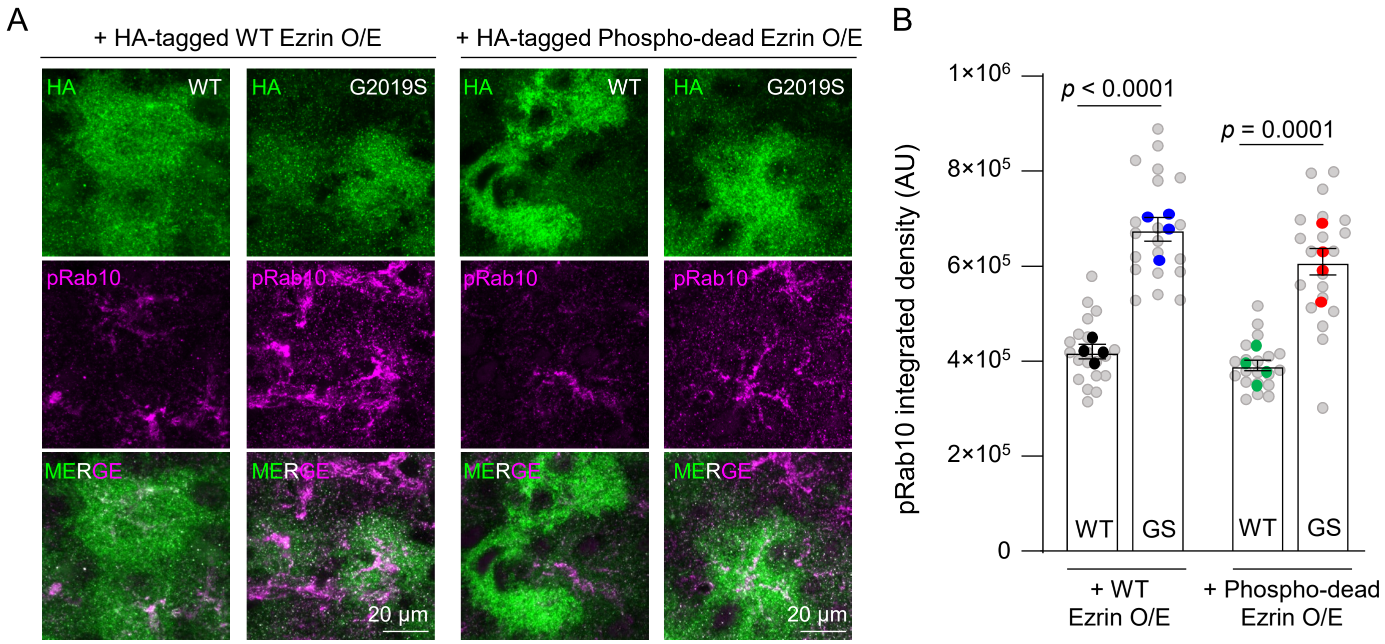

Figure S8: Overexpression of AAV-HA-tagged-WT Ezrin and AAV-HA-tagged-Phospho-dead Ezrin in WT and LRRK2 G2019S$^{ki/ki}$ astrocytes. Related to Figure 4.

(A) Representative images from the ventral ACC of WT and LRRK2 G2019S$^{ki/ki}$ mice injected with AAV-HA-tagged WT Ezrin or Phospho-dead Ezrin, stained with HA and phospho-Rab10 antibodies at P84. Scale bar, 20 µm. **(B)** Quantification of phospho-Rab10 integrated density. n = 4 per group (2 males and 2 females). One-way ANOVA [$F(3,12) = 38.34$, $p < 0.0001$] with Bonferroni's post hoc test revealed significant differences between groups, except WT + WT Ezrin O/E vs. WT + Phospho-dead Ezrin O/E ($p > 0.9999$) and LRRK2 G2019S$^{ki/ki}$ groups ($p = 0.3303$).

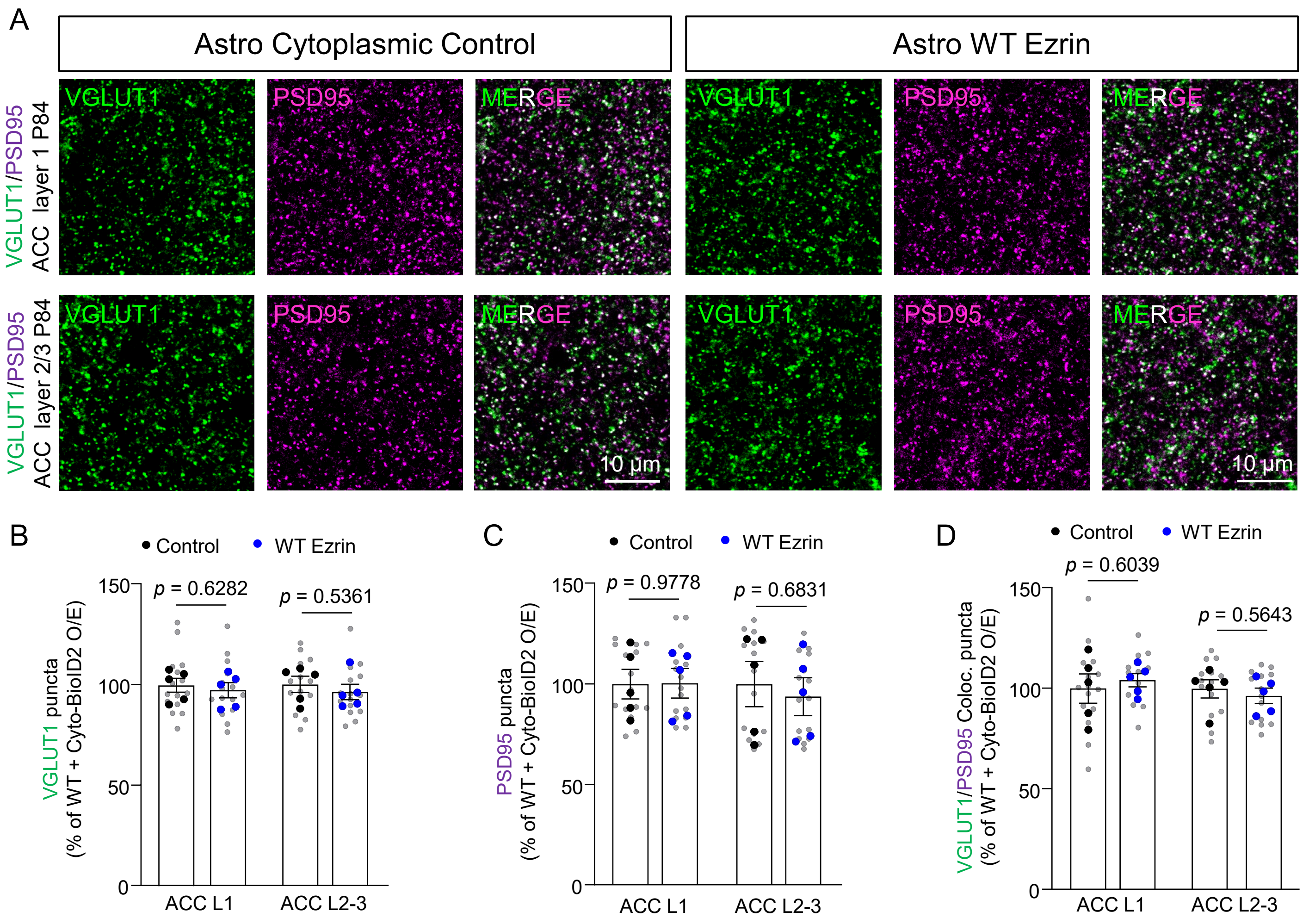

Figure S9: WT Ezrin overexpression does not change excitatory synapse numbers in the ACC of WT mice. Related to Figure 4.

(A) Representative images from the ventral ACC of WT mice injected with AAV-HA-tagged BirA2 (cytoplasmic control) or WT Ezrin that were stained with VGLUT1 and PSD95 antibodies at P84. Scale bar, 10 µm. **(B)** Quantification of VGLUT1 puncta, normalized using the means of BirA2 values in the ventral ACC. n = 5 mice per group. For ACC L1, nested t-test, unpaired two-tailed t-test. t (8) = 0.5035, *p* = 0.6282. For ACC L2/3, nested t-test, unpaired two-tailed t-test. t (8) = 0.6465, *p* = 0.5361. **(C)** Quantification of PSD95 puncta, normalized using the means of BirA2 values in the ventral ACC. n = 5 mice per group. For ACC L1, nested t-test, unpaired two-tailed t-test. t (8) = 0.02872, *p* = 0.9778. For ACC L2/3, nested t-test, unpaired two-tailed t-test. t (8) = 0.4235, *p* = 0.6831. **(D)** Quantification of VGLUT1-PSD95 puncta, normalized using the means of BirA2 values in the ventral ACC. n = 5 mice per group. For ACC L1, nested t-test, unpaired two-tailed t-test. t (8) = 0.5400, *p* = 0.6039. For ACC L2/3, nested t-test, unpaired two-tailed t-test. t (8) = 0.6013, *p* = 0.5643.

Figure S10 (related to Figure 4) **Wang et al.,**

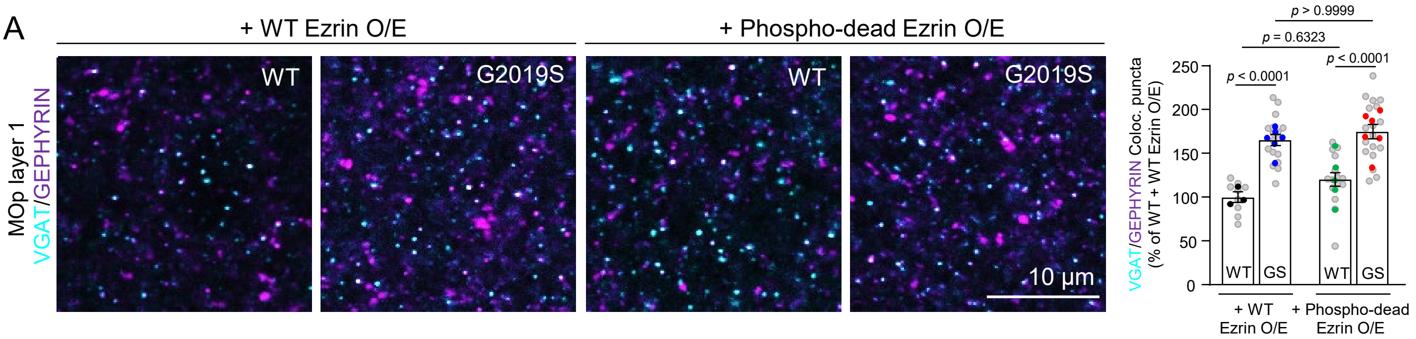

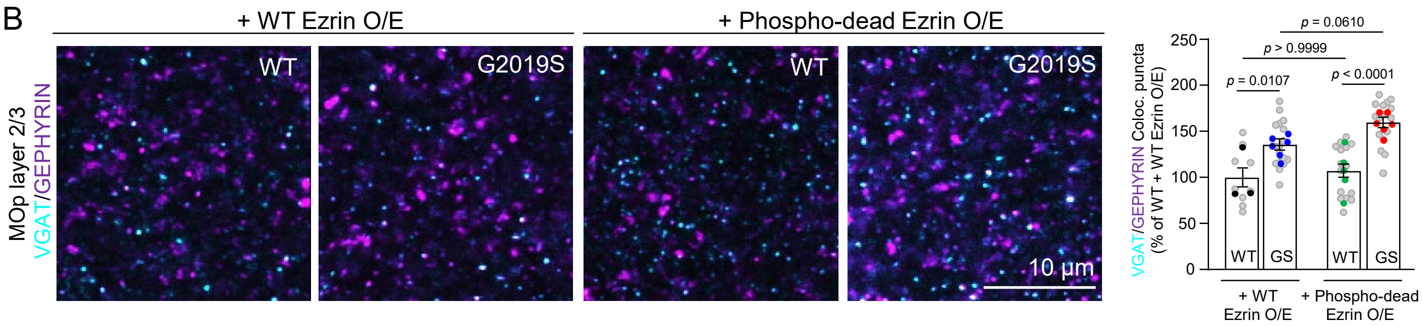

Figure S10: Overexpression of AAV-HA-tagged-WT Ezrin and AAV-HA-tagged-Phospho-dead Ezrin in WT and LRRK2 G2019S$^{ki/ki}$ astrocytes. Related to Figure 4.
(A) Representative images from the MOp L1 of the same groups stained with VGAT and GEPHYRIN antibodies. Scale bar, 10 µm. Quantification of VGAT-GEPHYRIN co-localized puncta, normalized to WT + WT Ezrin O/E. n = 3–6 per group. One-way ANOVA [F(3,55) = 19.7, $p < 0.0001$] with Bonferroni's post hoc test revealed significant differences between WT + WT Ezrin O/E and LRRK2 G2019S$^{ki/ki}$ + WT Ezrin O/E, and between WT + Phospho-dead Ezrin O/E and LRRK2 G2019S$^{ki/ki}$ + Phospho-dead Ezrin O/E ($p < 0.0001$). No differences were found between WT or LRRK2 G2019S$^{ki/ki}$ groups within Ezrin conditions ($p > 0.05$). **(B)** Representative images from the MOp L2-3 of WT and LRRK2 G2019S$^{ki/ki}$ mice injected with AAV-HA-tagged WT Ezrin or Phospho-dead Ezrin, stained with VGAT and GEPHYRIN antibodies at P84. Scale bar, 10 µm. Quantification of VGAT-GEPHYRIN co-localized puncta normalized to WT + WT Ezrin O/E. n = 3–6 per group. One-way ANOVA [F(3,54) = 15.10, $p < 0.0001$] with Bonferroni's post hoc test showed significant differences between WT + WT Ezrin O/E and LRRK2 G2019S$^{ki/ki}$ + WT Ezrin O/E ($p = 0.0107$) and between WT + Phospho-dead Ezrin O/E and LRRK2 G2019S$^{ki/ki}$ + Phospho-dead Ezrin O/E ($p < 0.0001$). No differences were observed between WT groups ($p > 0.9999$) or between LRRK2 G2019S$^{ki/ki}$ groups ($p = 0.0610$).

Figure S11 (related to Figure 5)

Wang et al.,

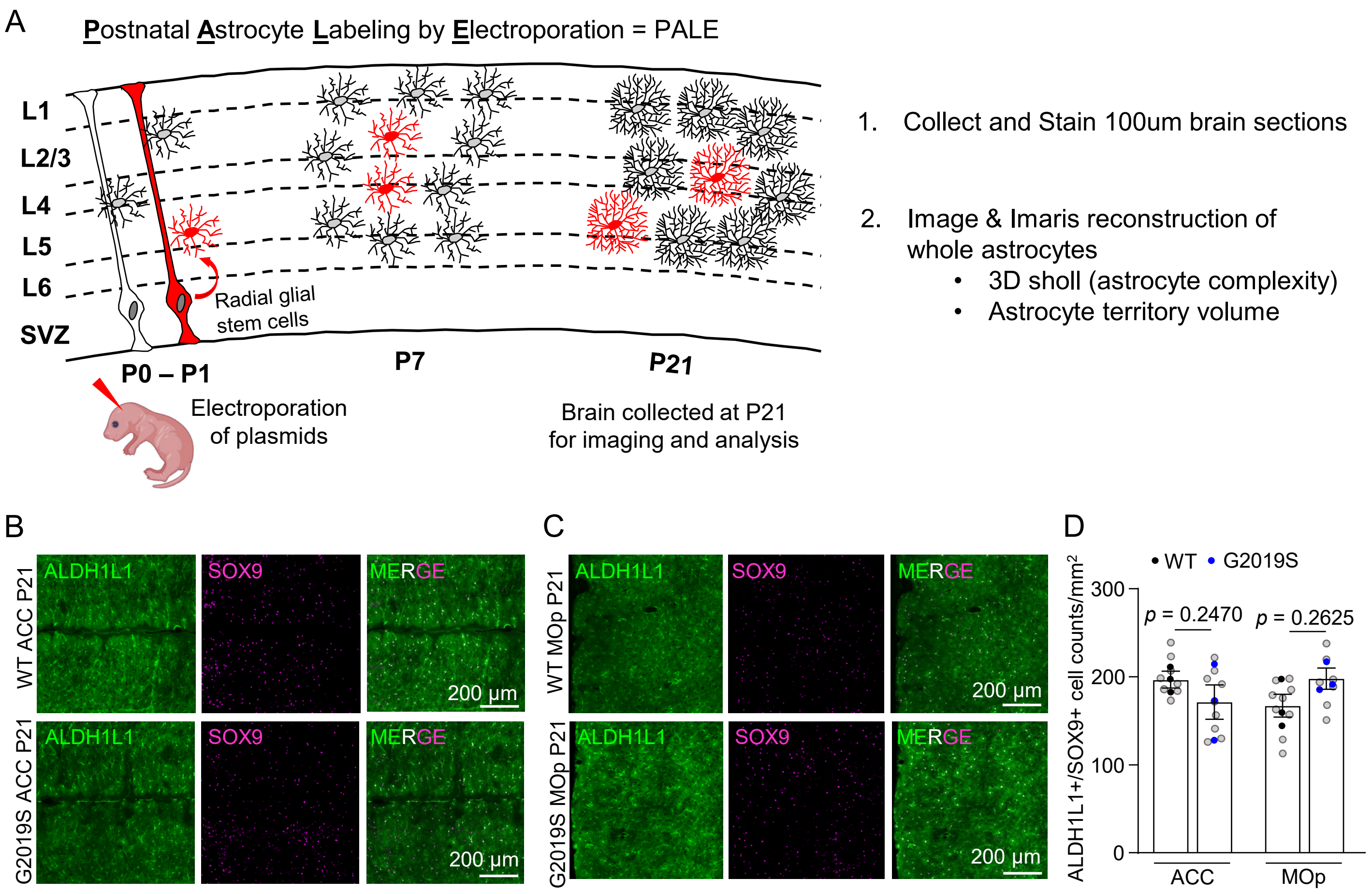

Figure S11: LRRK2 G2019S does not change ALDH1L1+/SOX9+ cell numbers in the ACC and MOp. Related to Figure 5.
(A) Overview of postnatal astrocyte labeling by electroporation (PALE) with PiggyBac plasmids. **(B-C)** Representative confocal images of SOX9 (purple) in the ACC and MOp of WT or LRRK2 G2019S$^{ki/ki}$ Aldh1l1-eGFP mice at P21. Scale bar, 200 µm. **(D)** Quantification of ALDH1L1+/SOX9+ cell numbers in (B-C), n = 3 (WT, 2 males and 1 female), 3 (LRRK2 G2019S$^{ki/ki}$, 2 males and 1 female) mice, For ALDH1L1+/SOX9+ cell counting in the ACC, nested t-test, unpaired Two-tailed t-test. t (4) = 0.9619, *p* = 0.3906. For ALDH1L1+/SOX9+ cell counting in the MOp, nested t-test, unpaired two-tailed t-test. t (4) = 1.684, *p* = 0.1675. Grey dots are the data acquired from each image. Black dots are the averaged data acquired from each WT mouse. Blue dots are the averaged data acquired from each LRRK2 G2019S$^{ki/ki}$ mouse.

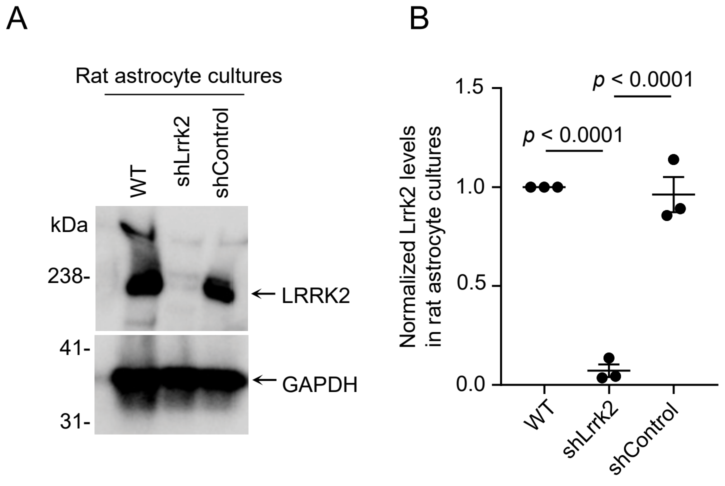

Figure S12: Expression of LRRK2 in astrocytes. Related to Figure 5.
(A) Cultured WT, shControl, and shLrrk2 transfected rat cortical astrocytes were lysed, and cytoplasmic proteins were subjected to Western blotting using LRRK2 and GAPDH antibodies. Results are representative of 3 independent experiments. **(B)** Densitometric analysis of LRRK2 levels in (E). Signals corresponding to LRRK2 were first normalized to that for β-actin. Relative LRRK2 levels were then normalized to the LRRK2 signals in WT rat cortical astrocytes. Statistical significance was determined by One-way ANOVA [F (2, 6) = 92.25, $p < 0.0001$], Bonferroni multiple comparisons revealed a significant difference between WT and shLrrk2 ($p < 0.0001$, 95% C.I. = [0.6743, 1.183]) and between shLrrk2 and shControl ($p < 0.0001$, 95%C.I. = [-1.145, -0.6363]) and no differences between WT and shControl ($p > 0.9999$, 95%C.I. = [-0.2165, 0.2923]), alpha = 0.05.

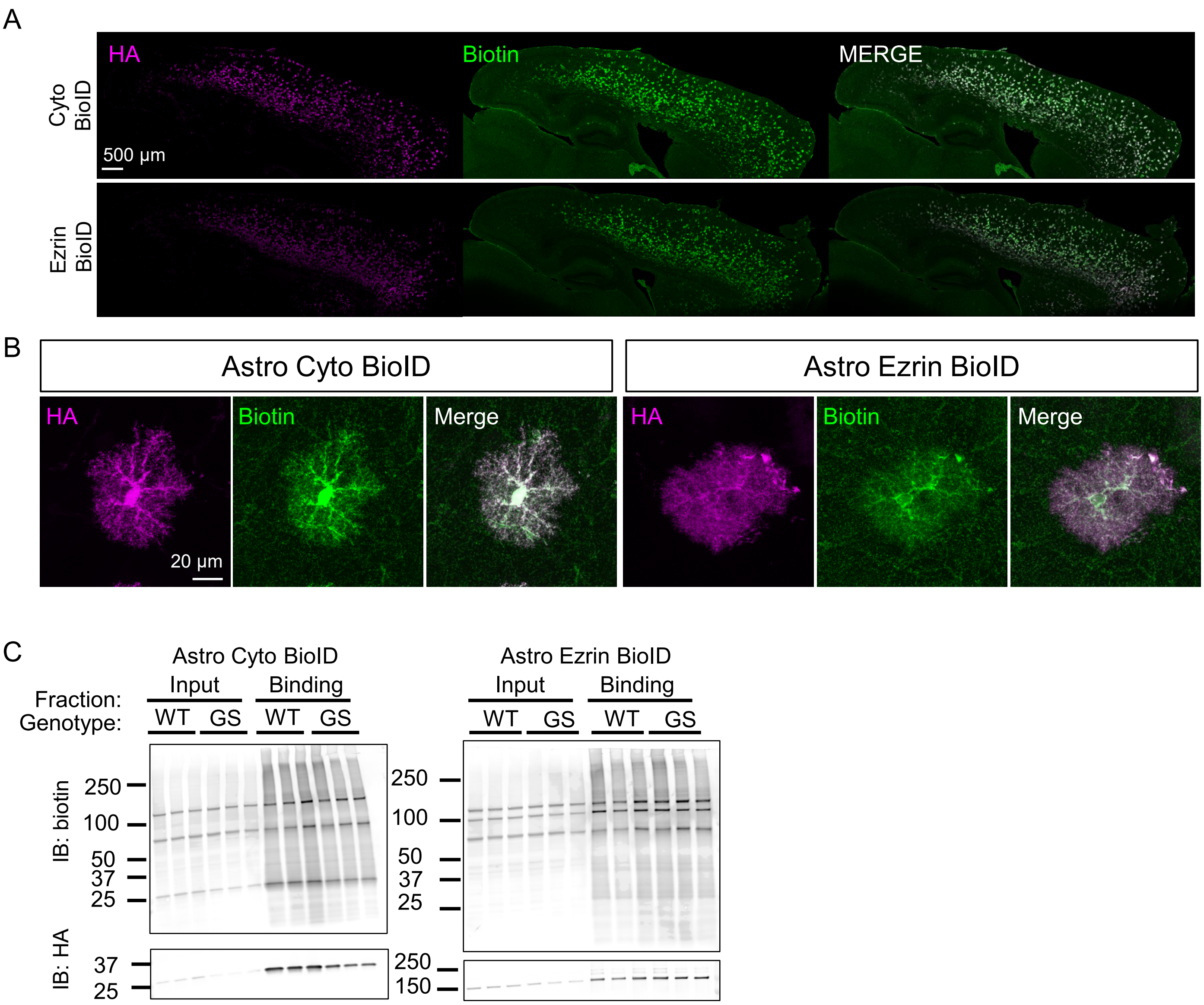

Figure S13: BioID constructs expression and biotinylation *in vivo* and *in vitro*. Related to Figure 6.

(A-B) Representative images of *in vivo* expression in the cortex of different BioID constructs labeled with HA and the biotinylating activity labeled with streptavidin. Merged images show the colocalization of HA and biotin signals in astrocytes. Scale bar, 500 µm (A). Scale bar, 20 µm (B). **(C)** Western blot analysis of BioID constructs expression (HA) and biotinylation activity (Streptavidin) *in vitro* in cortical lysates and subsequent immunoprecipitation.

Figure S14 (related to Figure 6) **Wang et al.,**

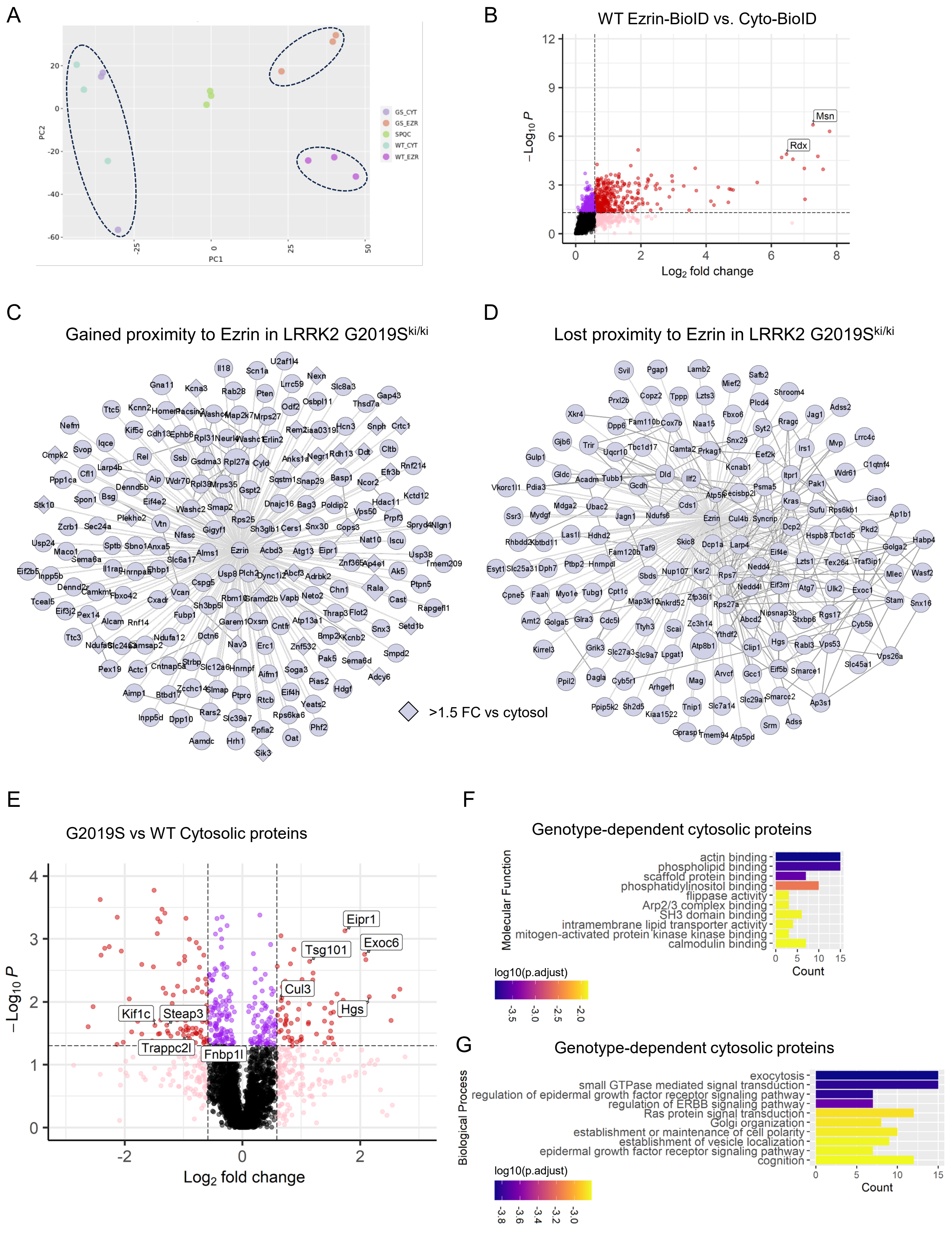

Figure S14: LRRK2 G2019S changes the composition of cytoplasmic proteins in astrocytes *in vivo*. Related to Figure 6.
(A) Principle component analyses (PCA) of Cyto-BioID (control) and Ezrin-BioID (bait) samples. **(B)** The plot shows that Radixin and Moesin, known as Ezrin interacting proteins, are significantly enriched in the Ezrin-BioID sample compared to Cyto-BioID. **(C-D)** Interaction networks depict Ezrin interactions detected by BioID and known protein-protein interactions identified in the publicly available stringDB database for proteins with gained (C) or lost (D) proximity to Ezrin in LRRK2 G2019S$^{ki/ki}$ astrocytes compared to WT astrocytes. **(E)** Volcano plot showing the differential abundance of cytosolic proteins in WT and LRRK2 G2019S$^{ki/ki}$ cortices. **(F-G)** Bars show the top 10 most significant Gene Ontology (GO) terms, ordered by highest gene count and lowest adjusted p-value, for the proteins differentially detected by Astro-cyto-BioID in WT compared to LRRK2 G2019S$^{ki/ki}$ (F) Molecular function (G) Biological Process.

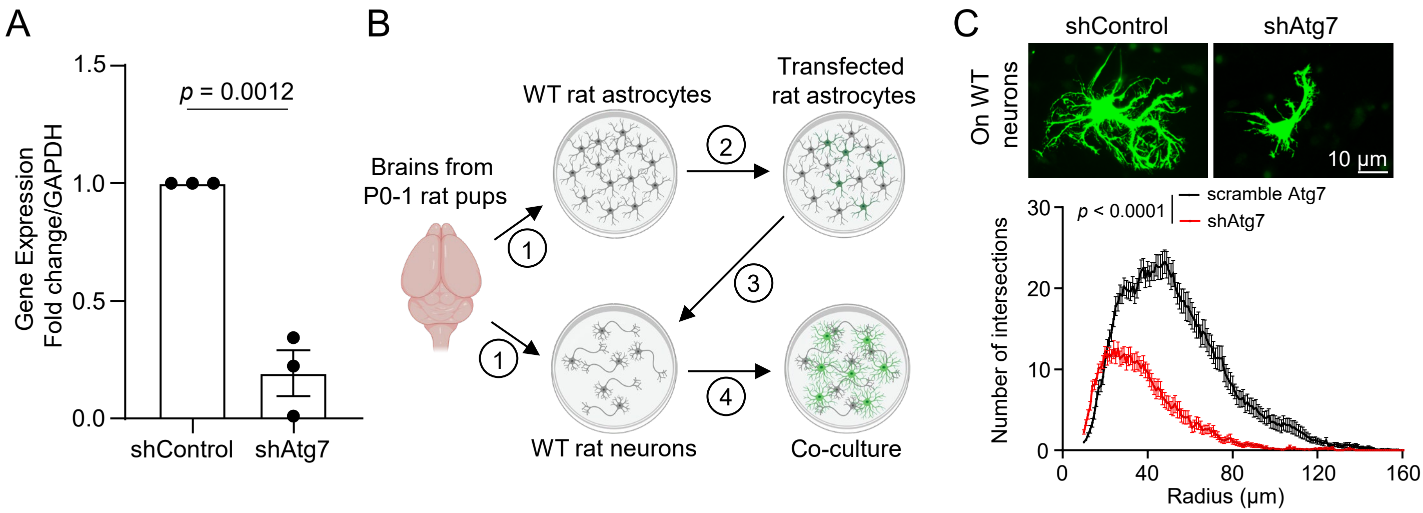

Figure S15: Astrocytic Atg7 is essential astrocyte morphological complexity *in vitro*. Related to Figure 7.

(A) Comparable expression of Atg7 mRNA transcripts by RT-PCR in N2A cells transfected with scrambled shRNA (shControl-GFP) or shRNA targeting Atg7 (shAtg7-GFP). n = 3 independent cultures. unpaired Two-tailed t-test. t (4) = 8.258, $p = 0.0012$. **(B)** Schematic of astrocyte-neuron co-culture assay. **(C)** (Upper) Rat cortical astrocytes transfected with scrambled shRNA (shControl-GFP) or shRNA targeting Atg7 (shAtg7-GFP) co-cultured with wild-type cortical neurons. Scale bar, 10 µm. (Lower) Quantification of astrocyte branching complexity. n = 30 (shControl-GFP), 30 (shAtg7-GFP) astrocytes co-cultured with WT cortical neurons compiled from two independent experiments. We fitted a linear mixed model with a number of intersections as the outcome variable, condition as a predictor, and a number of cells and cell radius entered as random effects. Within this model, shAtg7 (beta = 2.91, t(58) = 7.14) led to a significant decrease in the number of intersections compared to shControl astrocytes (beta = 7.89, t(58) = 19.35). Tukey multiple comparisons test revealed a significant difference between shControl and shAtg7 astrocytes co-cultured on WT neurons ($p < 0.0001$).
