# Supplementary material for: Astrocytic LRRK2 Controls Synaptic Connectivity via Regulation of ERM Phosphorylation
Source: bioRxiv. 2024 Aug 28:2023.04.09.536178. Preprint. [Version 2] doi: 10.1101/2023.04.09.536178 (PMC11383028; doi:10.1101/2023.04.09.536178)
Supplement: Supplementary file 1 [file media-1.pdf]

Supplemental information

Astrocytic LRRK2 controls synaptic connectivity via regulation of ERM phosphorylation

Shiyi Wang, Ryan Baumert, Gabrielle Séjourné, Dhanesh Sivadasan Bindu, Kylie Dimond, Kristina Sakers, Leslie Vazquez, Jessica Moore, Christabel Xin Tan, Tetsuya Takano, Maria Pia Rodriguez, Scott H. Soderling, Albert R. La Spada, and Cagla Eroglu

Figure S1 (related to Figure 1) **Wang et al.,**

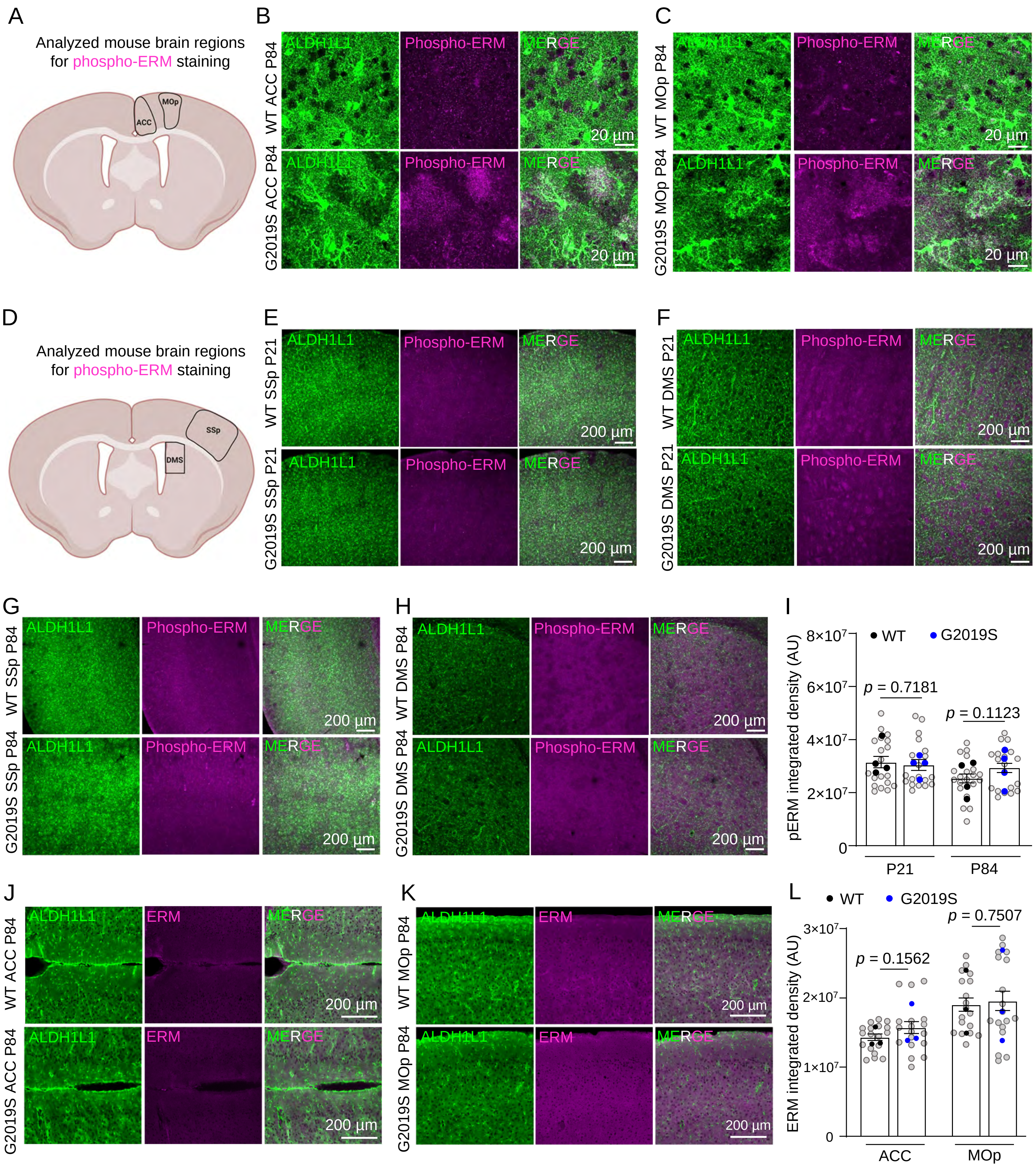

Figure S1: Phospho-ERM is upregulated in LRRK2 G2019S$^{ki/ki}$ astrocytes in ACC and MOp but not SSp and DMS. Related to Figure 1.

(A) Schematic representation of analyzed mouse brain regions for phospho-ERM staining, the ACC, and the MOp. **(B-C)** Representative confocal images of ERM phosphorylation (purple) in the ACC and MOp of WT or LRRK2 G2019S$^{ki/ki}$ Aldh1L1-eGFP mice at P84. Scale bar, 20 μm. **(D)** Schematic representation of analyzed mouse brain regions for phospho-ERM staining, the somatosensory cortex (SSp), and the dorsal medial striatum (DMS). **(E-H)** Representative confocal images of ERM phosphorylation (purple) in the SSp and DMS of WT or LRRK2 G2019S$^{ki/ki}$ Aldh1L1-eGFP mice at P21 (E-F) and at P84 (G-H). Scale bar, 200 μm. **(I)** Quantification of phospho-ERM integrated density in (E-H). For phospho-ERM integrated density at P21, nested t-test, unpaired two-tailed t-test. t (8) = 0.3740, *p* = 0.7181. n = 4 (WT, 2 males and 2 females), 4 (LRRK2 G2019S$^{ki/ki}$, 2 males and 2 females) mice. For phospho-ERM integrated density at P84, nested t-test, unpaired two-tailed t-test. t (38) = 1.626, *p* = 0.1123. n = 4 (WT, 2 males and 2 females), 4 (LRRK2 G2019S$^{ki/ki}$, 2 males and 2 females) mice. **(J-K)** Representative confocal images of ERM (purple) in the primary motor cortex (MOp) and the anterior cingulate cortex (ACC) of WT or LRRK2 G2019S$^{ki/ki}$ Aldh1L1-eGFP mice at P21. Scale bar, 200 μm. **(L)** Quantification of ERM integrated density in (J-K), n = 3 (WT, 2 males and 1 female), 3 (LRRK2 G2019S$^{ki/ki}$, 2 males and 1 female) mice, For ERM quantification in the ACC, nested t-test, unpaired two-tailed t-test. t (34) = 1.45, *p* = 0.1562. For ERM quantification in the MOp, nested t-test, unpaired two-tailed t-test. t (34) = 0.3203, *p* = 0.7507. Grey dots are the data acquired from each image. Black dots are the averaged data acquired from each WT mouse. Blue dots are the averaged data acquired from each LRRK2 G2019S$^{ki/ki}$ mouse.

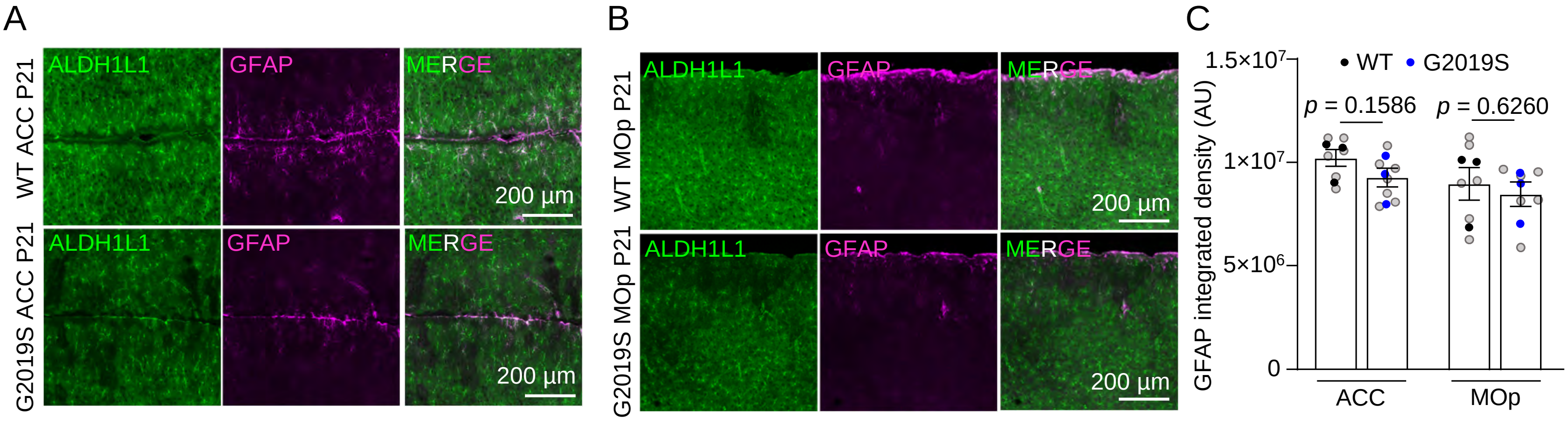

Figure S2: GFAP level is not altered in LRRK2 G2019S$^{ki/ki}$ astrocytes in ACC and MOp. Related to Figure 1.

(A-B) Representative confocal images of GFAP (purple) in the ACC and MOp of WT or LRRK2 G2019S$^{ki/ki}$ Aldh1l1-eGFP mice at P21. Scale bar, 200 µm. **(C)** Quantification of GFAP integrated density in (A-B), n = 3 (WT, 2 males and 1 female), 3 (LRRK2 G2019S$^{ki/ki}$, 2 males and 1 female) mice, For GFAP quantification in the ACC, nested t-test, unpaired two-tailed t-test. t (10) = 1.524, p = 0.1586. For GFAP quantification in the MOp, nested t-test, unpaired two-tailed t-test. t (10) = 0.5028, p = 0.6260. Grey dots are the data acquired from each image. Black dots are the averaged data acquired from each WT mouse. Blue dots are the averaged data acquired from each LRRK2 G2019S$^{ki/ki}$ mouse.

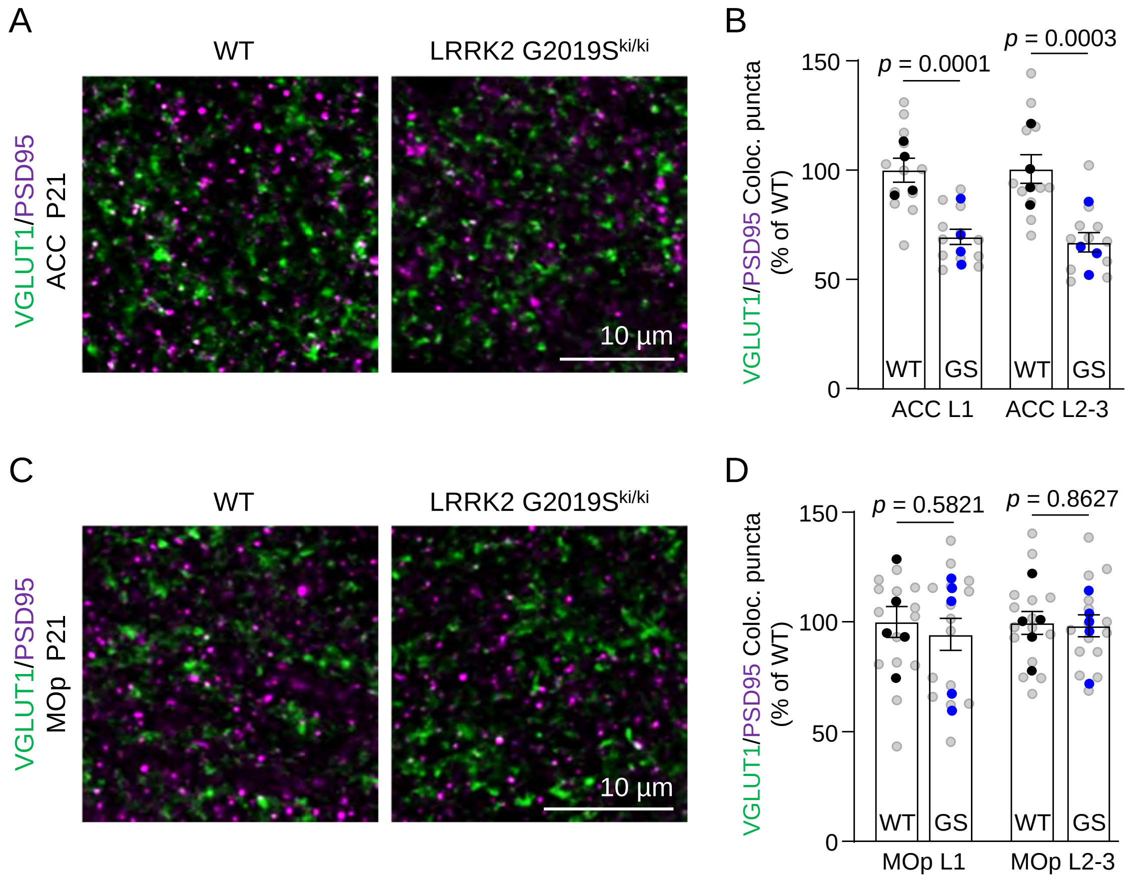

Figure S3: LRRK2 G2019S does not change excitatory synapse numbers in the primary motor cortex. Related to Figure 2.

(A) Representative images from the ventral ACC of WT and LRRK2 G2019S$^{ki/ki}$ mice that were stained with VGluT1 and PSD95 antibodies at P21. Scale bar, 10 µm. **(B)** Quantification of VGluT1-PSD95 co-localized puncta, normalized using the means of WT values in the ventral ACC. n = 4 (WT, 2 males and 2 females), 4 (LRRK2 G2019S$^{ki/ki}$, 2 males and 2 females) mice. Nested One-way ANOVA [F (3, 44) = 12.98, p < 0.0001], Bonferroni's multiple comparisons test revealed a significant difference between WT ACC L1 and LRRK2 G2019S$^{ki/ki}$ ACC L1 (p = 0.0002, 95% C.I. = [13.71, 47.33]), and between WT ACC L2-3 and LRRK2 G2019S$^{ki/ki}$ ACC L2-3 (p < 0.0001, 95% C.I. = [16.48, 50.10]). alpha = 0.05. **(C)** Representative images from the MOp of WT and LRRK2 G2019S$^{ki/ki}$ mice that were stained with VGluT1 and PSD95 antibodies at P21. Scale bar, 10 µm. **(D)** Quantification of VGluT1-PSD95 co-localized puncta, normalized using the means of WT values in the MOp L1 and L2-3. n = 5 (WT), 5 (LRRK2 G2019S$^{ki/ki}$) mice, 3 males and 2 females. Nested One-way ANOVA [F (3, 56) = 0.1837, p = 0.9070], Bonferroni's multiple comparisons test revealed a significant difference between WT MOp L1 and LRRK2 G2019S$^{ki/ki}$ MOp L1 (p > 0.9999, 95% C.I. = [-14.69, 25.97]), and between WT MOp L2-3 and LRRK2 G2019S$^{ki/ki}$ MOp L2-3 (p > 0.9999, 95% C.I. = [-19.06, 21.61]). alpha = 0.05. Grey dots are the data acquired from each image. Black dots are the averaged data acquired from each WT mouse. Blue dots are the averaged data acquired from each LRRK2 G2019S$^{ki/ki}$ mouse.

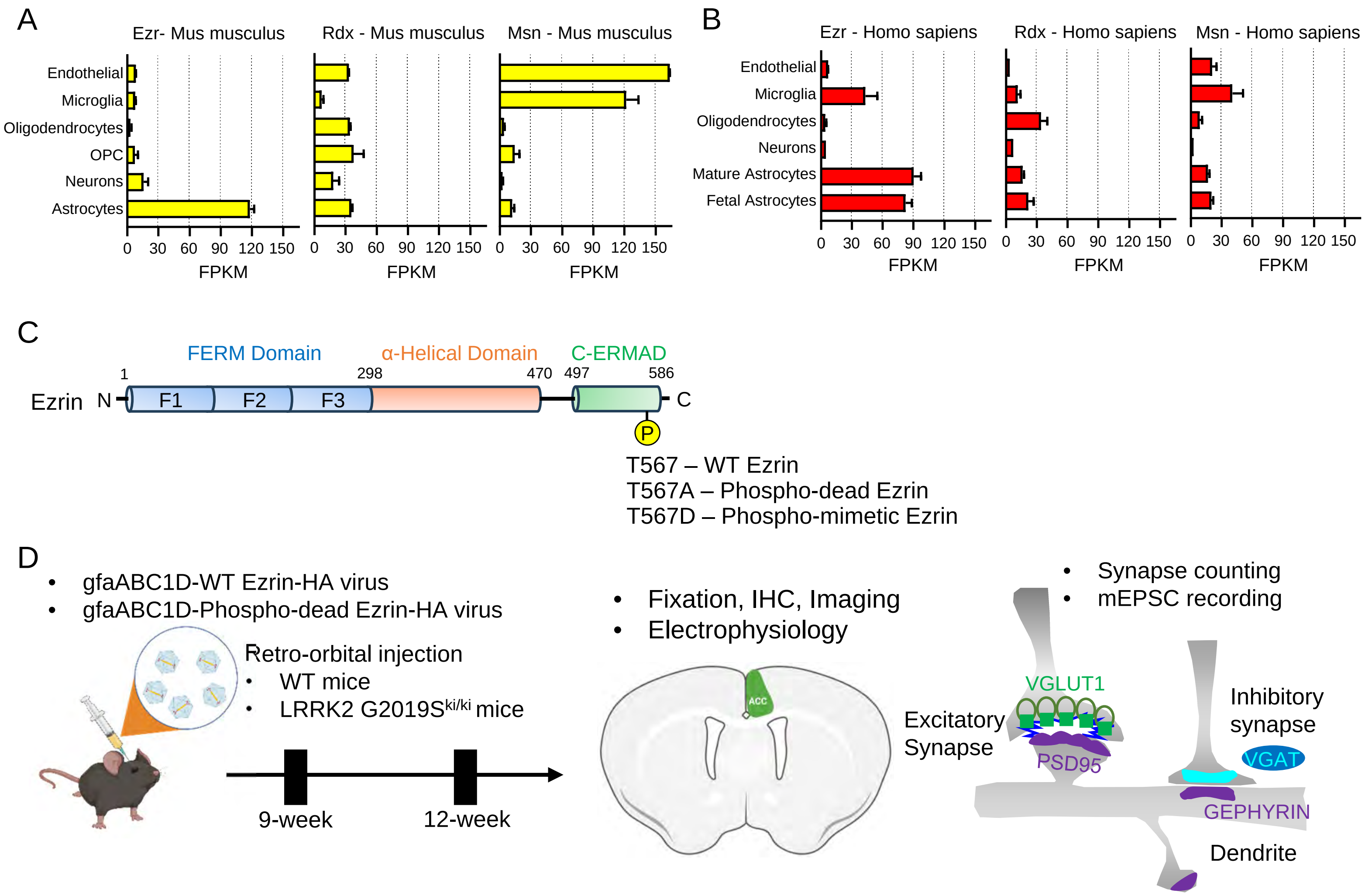

Figure S4: Overexpression of AAV-HA-tagged-WT Ezrin and AAV-HA-tagged-Phospho-dead Ezrin in WT and LRRK2 G2019S$^{ki/ki}$ astrocytes. Related to Figure 4.

(A-B) Bar plots of Ezr, Rdx, and Msn gene expression in various brain cell types in mice (yellow) or humans (red). These plots are generated using publicly available data at https://www.brainrnaseq.org **(C)** Schematic of domains within Ezrin. **(D)** Experiment workflow of AAV-HA-tagged-WT Ezrin and AAV-HA-tagged-Phospho-dead Ezrin in WT and LRRK2 G2019S$^{ki/ki}$ astrocytes.

Figure S5 (related to Figure 4)

Wang et al.,

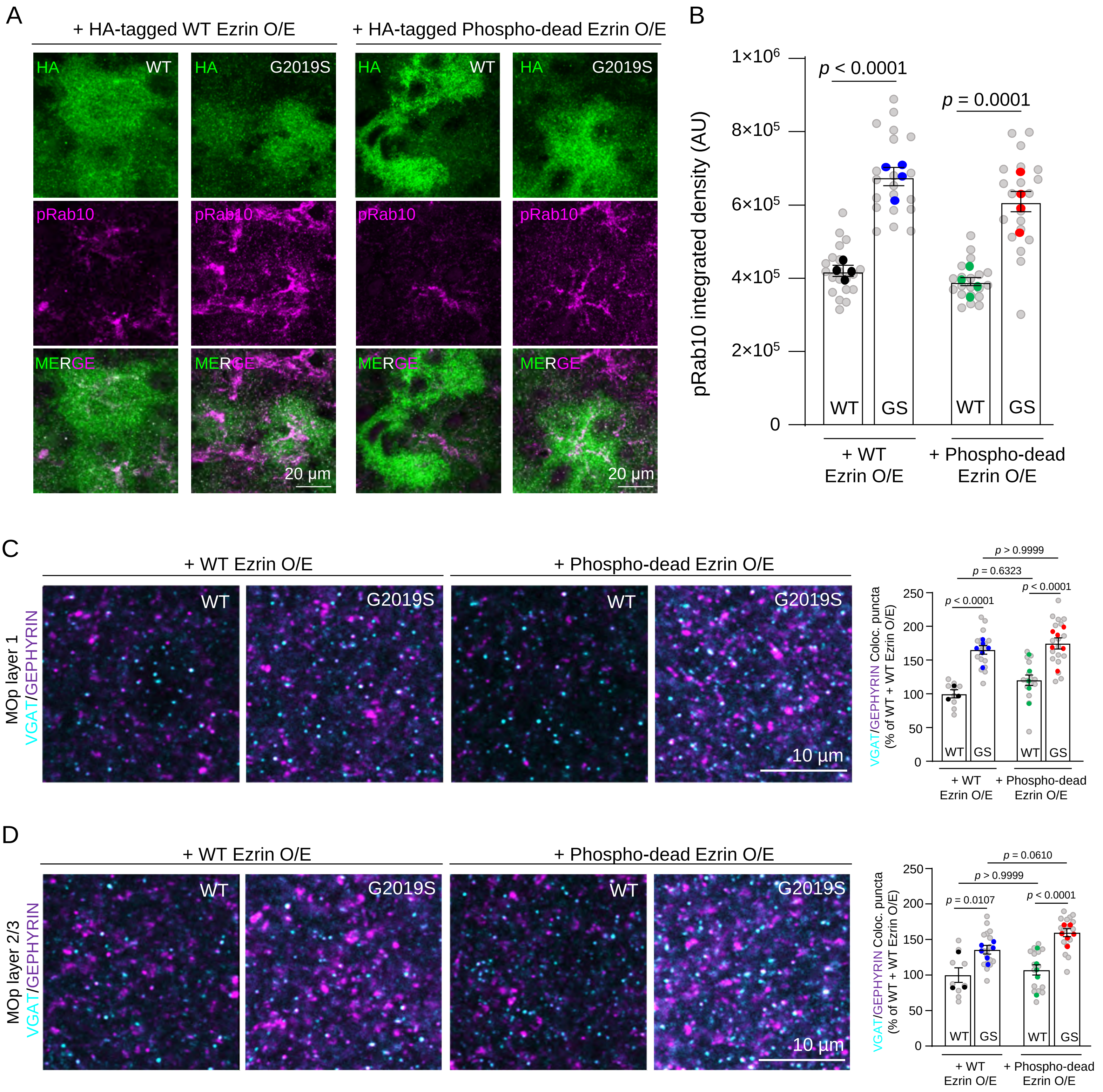

Figure S5: Overexpression of AAV-HA-tagged-WT Ezrin and AAV-HA-tagged-Phospho-dead Ezrin in WT and LRRK2 G2019S$^{ki/ki}$ astrocytes. Related to Figure 4.

(A) Representative images from the ventral ACC of WT and LRRK2 G2019S$^{ki/ki}$ mice injected with AAV-HA-tagged-WT Ezrin or AAV-HA-tagged-Phospho-dead Ezrin that were stained with HA and phospho-Rab10 antibodies at P84. Scale bar, 200 μm. **(B)** Quantification of phospho-Rab10 integrated density in (A). n = 4 (WT + WT Ezrin O/E, 2 males and 2 females), 4 (WT + Phospho-dead Ezrin O/E, 2 males and 2 females), 4 (LRRK2 G2019S$^{ki/ki}$ + WT Ezrin O/E, 2 males and 2 females), 4 (LRRK2 G2019S$^{ki/ki}$ + Phospho-dead Ezrin O/E, 2 males and 2 females) mice. One-way ANOVA [F (3, 12) = 38.34, $p < 0.0001$], Bonferroni's multiple comparisons test revealed a significant difference between WT + WT Ezrin O/E and LRRK2 G2019S$^{ki/ki}$ + WT Ezrin O/E ($p < 0.0001$, 95% C.I. = [-358150, -155861]), between WT + WT Ezrin O/E and LRRK2 G2019S$^{ki/ki}$ + Phospho-dead Ezrin O/E ($p = 0.0004$, 95% C.I. = [-289977, -87688]), between WT + Phospho-dead Ezrin O/E and LRRK2 G2019S$^{ki/ki}$ + WT Ezrin O/E ($p < 0.0001$, 95% C.I. = [-387427, -185138]), and between WT + Phospho-dead Ezrin O/E and LRRK2 G2019S$^{ki/ki}$ + Phospho-dead Ezrin O/E ($p = 0.0001$, 95% C.I. = [-319255, -116966]). There were no differences between WT + WT Ezrin O/E and WT + Phospho-dead Ezrin O/E ($p > 0.9999$, 95% C.I. = [-71867, 130422]), and between LRRK2 G2019S$^{ki/ki}$ + WT Ezrin O/E and LRRK2 G2019S$^{ki/ki}$ + Phospho-dead Ezrin O/E ($p = 0.3303$, 95% C.I. = [-32972, 169317]), alpha = 0.05. **(C)** Representative images from the MOp L1 of WT and LRRK2 G2019S$^{ki/ki}$ mice injected with AAV-HA-tagged-WT Ezrin or AAV-HA-tagged-Phospho-dead Ezrin that were stained with VGAT and GEPHYRIN antibodies at P84. Scale bar, 10 μm. Quantification of VGAT-GEPHYRIN co-localized puncta, normalized using the means of WT mice injected with AAV-HA-tagged-WT Ezrin in MOp L1. n = 3 (WT + WT Ezrin O/E, 2 males and 1 female), 5 (WT + Phospho-dead Ezrin O/E, 3 males and 2 females), 6 (LRRK2 G2019S$^{ki/ki}$ + WT Ezrin O/E, 3 males and 3 females), 6 (LRRK2 G2019S$^{ki/ki}$ + Phospho-dead Ezrin O/E, 3 males and 3 females) mice. One-way ANOVA [F (3, 55) = 19.7, $p < 0.0001$], Bonferroni's multiple comparisons test revealed a significant difference between WT + WT Ezrin O/E and LRRK2 G2019S$^{ki/ki}$ + WT Ezrin O/E ($p < 0.0001$, 95% C.I. = [-98.23, -32.48]), and between WT + Phospho-dead Ezrin O/E and LRRK2 G2019S$^{ki/ki}$ + Phospho-dead Ezrin O/E ($p < 0.0001$, 95% C.I. = [-82.37, -26.61]). There were no differences between WT + WT Ezrin O/E and WT + Phospho-dead Ezrin O/E ($p = 0.6323$, 95% C.I. = [-53.85, 13.40]), and between LRRK2 G2019S$^{ki/ki}$ + WT Ezrin O/E and LRRK2 G2019S$^{ki/ki}$ + Phospho-dead Ezrin O/E ($p > 0.9999$, 95% C.I. = [-36.33, 17.61]), alpha = 0.05. **(D)** Representative images from the MOp L2-3 of WT and LRRK2 G2019S$^{ki/ki}$ mice injected with AAV-HA-tagged-WT Ezrin or AAV-HA-tagged-Phospho-dead Ezrin that were stained with VGAT and GEPHYRIN antibodies at P84. Scale bar, 10 μm. Quantification of VGAT-GEPHYRIN co-localized puncta, normalized using the means of WT mice injected with AAV-HA-tagged-WT Ezrin in MOp L2-3. n = 3 (WT + WT Ezrin O/E, 2 males and 1 female), 5 (WT + Phospho-dead Ezrin O/E, 3 males and 2 females), 6 (LRRK2 G2019S$^{ki/ki}$ + WT Ezrin O/E, 3 males and 3 females), 6 (LRRK2 G2019S$^{ki/ki}$ + Phospho-dead Ezrin O/E, 3 males and 3 females) mice. One-way ANOVA [F (3, 54) = 15.10, $p < 0.0001$], Bonferroni's multiple comparisons test revealed a significant difference between WT + WT Ezrin O/E and LRRK2 G2019S$^{ki/ki}$ + WT Ezrin O/E ($p = 0.0107$, 95% C.I. = [-65.46, -5.94]), and between WT + Phospho-dead Ezrin O/E and LRRK2 G2019S$^{ki/ki}$ + Phospho-dead Ezrin O/E ($p < 0.0001$, 95% C.I. = [-78.07, -26.92]). There were no differences between WT + WT Ezrin O/E and WT + Phospho-dead Ezrin O/E ($p > 0.9999$, 95% C.I. = [-37.73, 23.15]), and between LRRK2 G2019S$^{ki/ki}$ + WT Ezrin O/E and LRRK2 G2019S$^{ki/ki}$ + Phospho-dead Ezrin O/E ($p = 0.0610$, 95% C.I. = [-48.84, 0.6819]), alpha = 0.05.

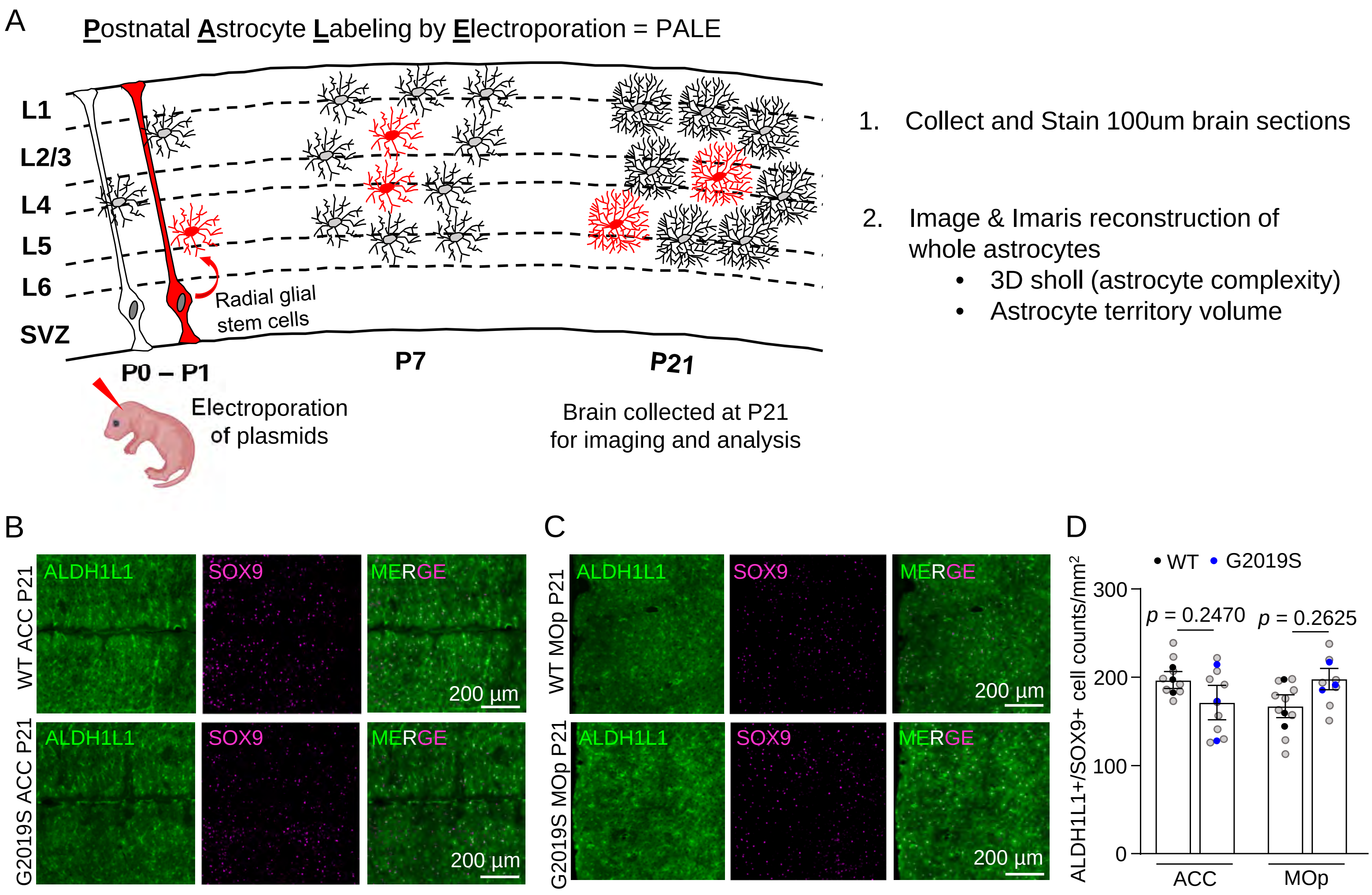

Figure S6: LRRK2 G2019S does not change ALDH1L1+/SOX9+ cell numbers in the ACC and MOp. Related to Figure 5.

(A) Overview of postnatal astrocyte labeling by electroporation (PALE) with PiggyBac plasmids. **(B-C)** Representative confocal images of SOX9 (purple) in the ACC and MOp of WT or LRRK2 G2019S$^{ki/ki}$ Aldh1l1-eGFP mice at P21. Scale bar, 200 µm. **(D)** Quantification of ALDH1L1+/SOX9+ cell numbers in (B-C), n = 3 (WT, 2 males and 1 female), 3 (LRRK2 G2019S$^{ki/ki}$, 2 males and 1 female) mice, For ALDH1L1+/SOX9+ cell counting in the ACC, nested t-test, unpaired Two-tailed t-test. t (9) = 1.533, p = 0.2470. For ALDH1L1+/SOX9+ cell counting in the MOp, nested t-test, unpaired two-tailed t-test. t (2) = 1.544, p = 0.2625. Grey dots are the data acquired from each image. Black dots are the averaged data acquired from each WT mouse. Blue dots are the averaged data acquired from each LRRK2 G2019S$^{ki/ki}$ mouse.

Figure S7 (related to Figure 5) **Wang et al.,**

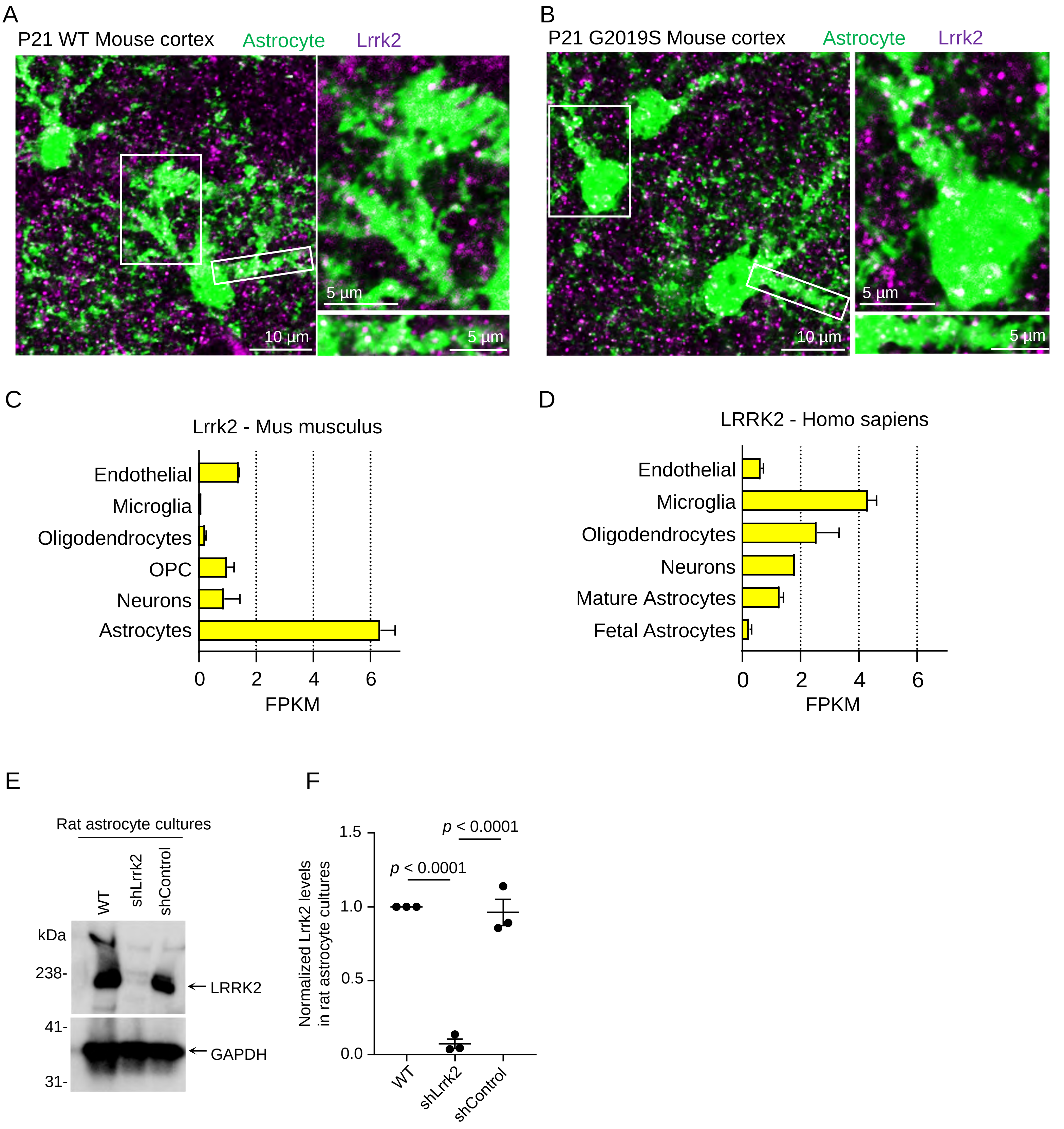

Figure S7: Localization and expression of LRRK2 in astrocytes. Related to Figure 5.
(A-B) LRRK2 (red) in the primary motor cortex (MOp) of WT and LRRK2 G2019S$^{ki/ki}$; Aldh1l1-eGFP mice at P21. Scale bar, 10 µm. **(C-D)** Bar plots of Lrrk2 gene expression in various brain cell types in mice and humans. **(E)** Cultured WT, shControl, and sh*Lrrk2* transfected rat cortical astrocytes were lysed, and cytoplasmic proteins were subjected to Western blotting using LRRK2 and GAPDH antibodies. Results are representative of 3 independent experiments. **(F)** Densitometric analysis of LRRK2 levels in (E). Signals corresponding to LRRK2 were first normalized to that for β-actin. Relative LRRK2 levels were then normalized to the LRRK2 signals in WT rat cortical astrocytes. Statistical significance was determined by One-way ANOVA [$F(2, 6) = 92.25$, $p < 0.0001$], Bonferroni multiple comparisons revealed a significant difference between WT and sh*Lrrk2* ($p < 0.0001$, 95% C.I. = [0.6743, 1.183]) and between sh*Lrrk2* and shControl ($p < 0.0001$, 95%C.I. = [-1.145, -0.6363]) and no differences between WT and shControl ($p > 0.9999$, 95%C.I. = [-0.2165, 0.2923]), alpha = 0.05.

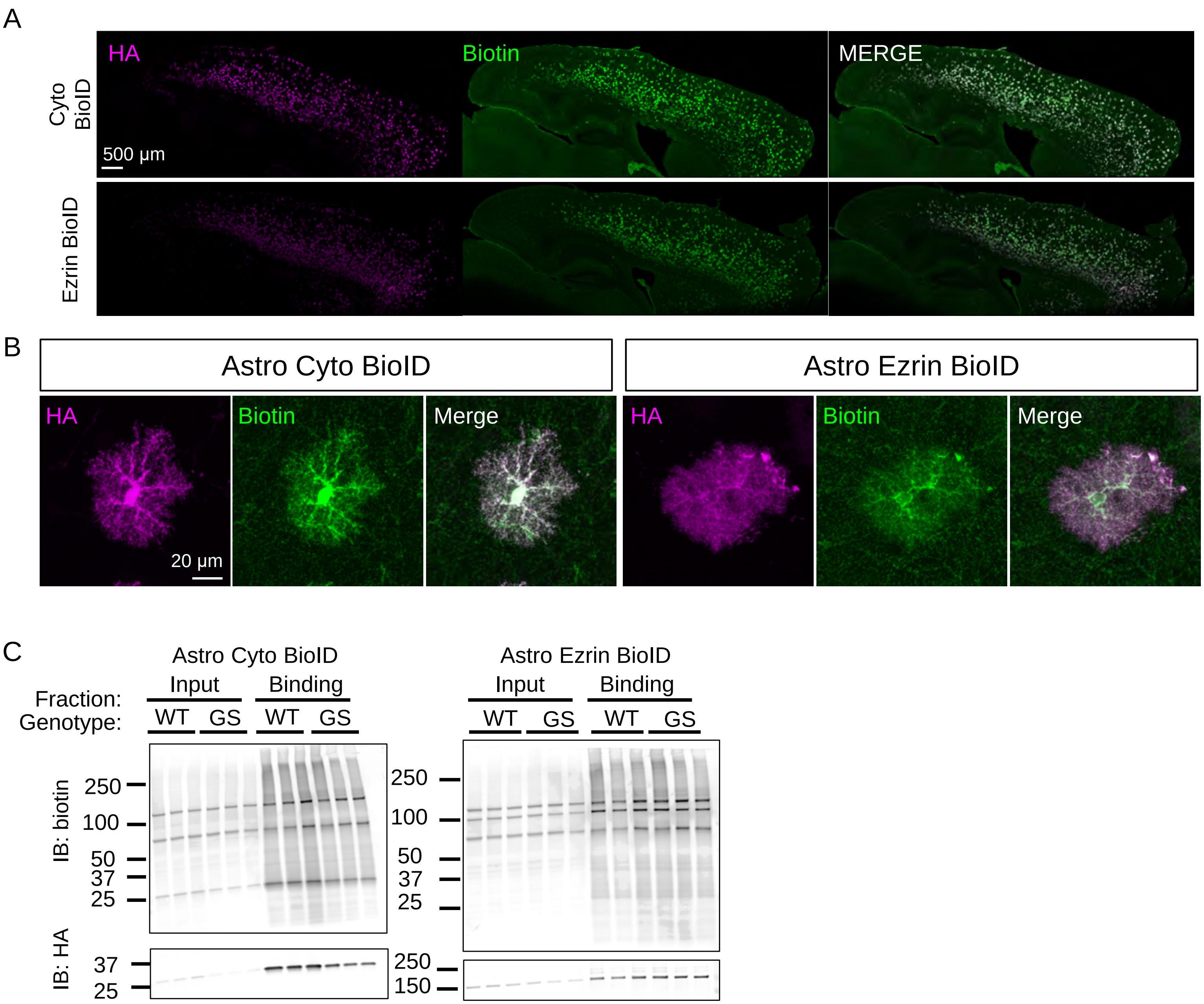

Figure S8: BioID constructs expression and biotinylation *in vitro* and *in vivo*. Related to Figure 6. **(A-B)** Representative images of in vivo expression in the cortex of different BioID constructs labeled with HA and the biotinylating activity labeled with streptavidin. Merged images show the colocalization of HA and biotin signals in astrocytes. Scale bar, 500 µm (A). Scale bar, 20 µm (B). **(C)** Western blot analysis of BioID constructs expression (HA) and biotinylation activity (Streptavidin) in vitro in cortical lysates and subsequent immunoprecipitation.

Figure S9 (related to Figure 6) **Wang et al.,**

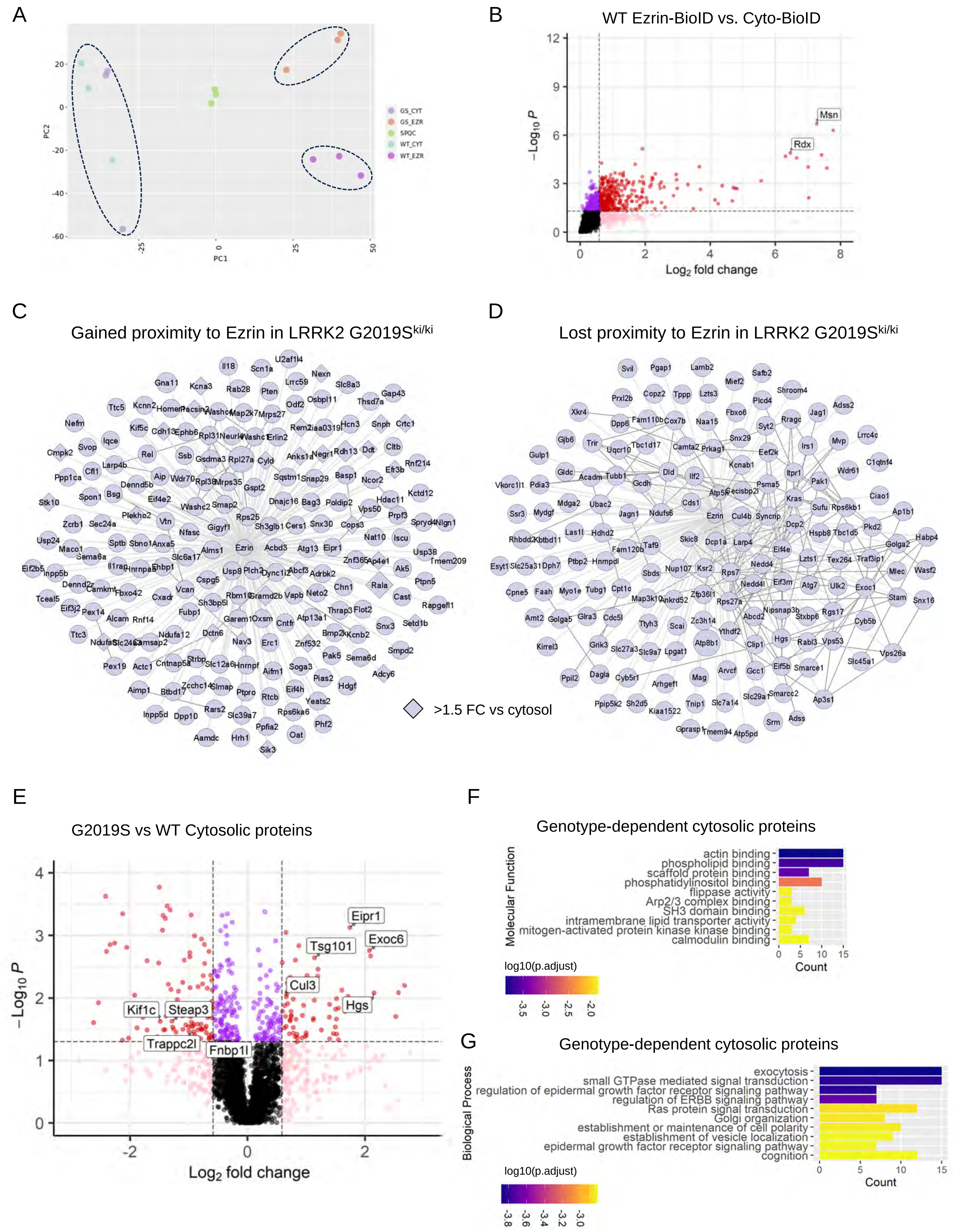

Figure S9: LRRK2 G2019S changes the composition of cytoplasmic proteins in astrocytes *in vivo*. Related to Figure 6.

(A) Principle component analyses (PCA) of Cyto-BioID (control) and Ezrin-BioID (bait) samples. **(B)** The plot shows that Radixin and Moesin, known as Ezrin interacting proteins, are significantly enriched in the Ezrin-BioID sample compared to Cyto-BioID. **(C-D)** Interaction networks depict Ezrin interactions detected by BioID and known protein-protein interactions identified in the publicly available stringDB database for proteins with gained (C) or lost (D) proximity to Ezrin in LRRK2 $G2019S^{ki/ki}$ astrocytes compared to WT astrocytes. **(E)** Volcano plot showing the differential abundance of cytosolic proteins in WT and LRRK2 $G2019S^{ki/ki}$ cortices. **(F-G)** Bars show the top 10 most significant Gene Ontology (GO) terms, ordered by highest gene count and lowest adjusted p-value, for the proteins differentially detected by Astro-cyto-BioID in WT compared to LRRK2 $G2019S^{ki/ki}$ (F) Molecular function (G) Biological Process.

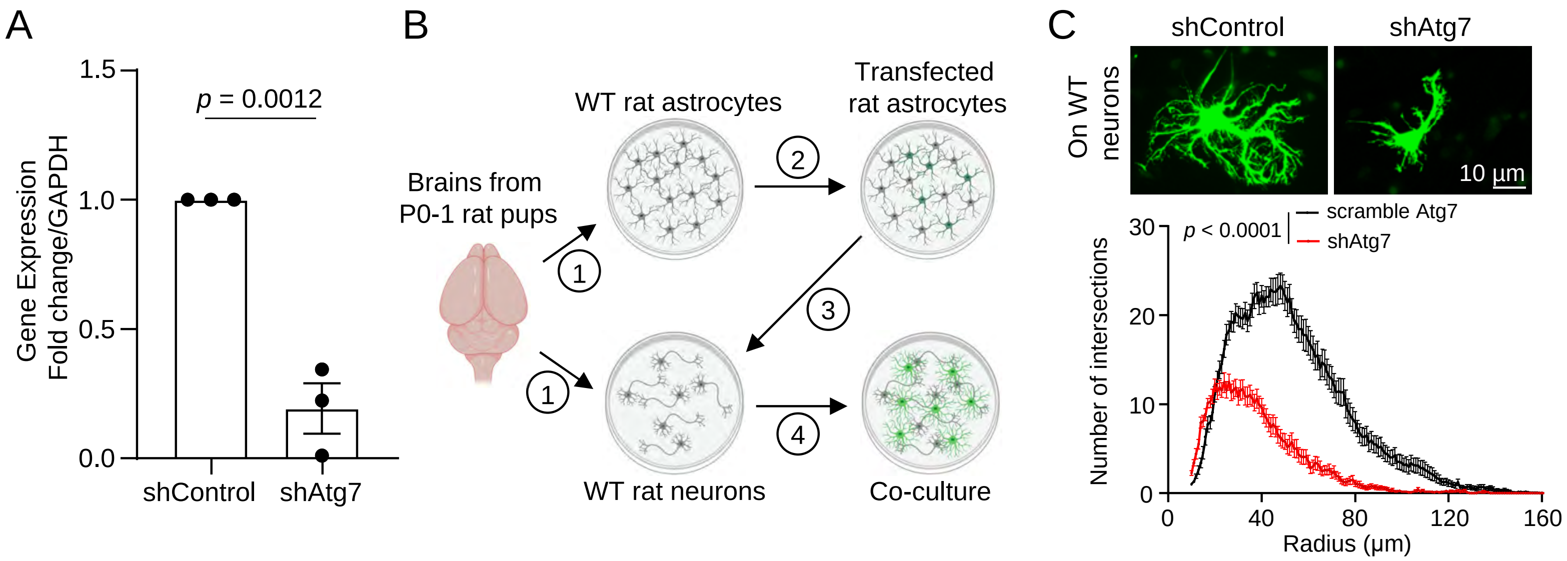

Figure S10: Astrocytic Atg7 is essential astrocyte morphological complexity *in vitro.* Related to Figure 7.

(A) Comparable expression of Atg7 mRNA transcripts by RT-PCR in N2A cells transfected with scrambled shRNA (shControl-GFP) or shRNA targeting Atg7 (shAtg7-GFP). n = 3 independent cultures. unpaired Two-tailed t-test. t (4) = 8.258, p = 0.0012. **(B)** Schematic of astrocyte-neuron co-culture assay. **(C)** (Upper) Rat cortical astrocytes transfected with scrambled shRNA (shControl-GFP) or shRNA targeting Atg7 (shAtg7-GFP) co-cultured with wild-type cortical neurons. Scale bar, 10 µm. (Lower) Quantification of astrocyte branching complexity. n = 30 (shControl-GFP), 30 (shAtg7-GFP) astrocytes co-cultured with WT cortical neurons compiled from two independent experiments. We fitted a linear mixed model with number of intersections as outcome variable, condition as predictor, and number of cells and cell radius were entered as random effects. Within this model, shAtg7 (beta = 2.91, t(58) = 7.14) led to a significant decrease of number of intersections compared to shControl astrocytes (beta = 7.89, t(58) = 19.35). Tukey multiple comparisons test revealed a significant difference between shControl and shAtg7 astrocytes co-cultured on WT neurons (p < 0.0001).

Supplement Table 1: Control and LRRK2 human brain sample information

CaseID	Mutation	MutationType	result	gender	expired_age	PMI
01-39	LRRK2	G2019s	positive	male	85	2
13-60	LRRK2	G2019s	positive	male	89	3.82
10-37	LRRK2	G2019s	positive	female	84	5

Control Human brain samples information

CaseID	DonorID	adcc_type	study_number	gender	expired_age	PMI
16-32	1008			male	79	4.3
04-52	991	ADCC Accepted	0300037	male	85	1.83
02-27	1294			male	86	3
03-63	999			female	83	3.25

PMI=Postmortem Interval In hours

High confidence gained interactions<br>*GS_EZR > WT_EZR & GS_EZR > GS_CYTO*														
	Normalized peptide intensity per sample												Fold change	
	**WT_CYT_1**	**WT_CYT_2**	**WT_CYT_3**	**WT_EZR_1**	**WT_EZR_2**	**WT_EZR_3**	**GS_CYT_1**	**GS_CYT_2**	**GS_CYT_3**	**GS_EZR_1**	**GS_EZR_2**	**GS_EZR_3**	**GS_EZR_v_G**	**WT_EZR_v_W**
Ddt	69922.3042	57463.0131	84921.0012	63852.1608	68325.9292	73727.019	46534.3266	65804.5839	71883.3415	113868.921	84363.7289	111489.73	1.68124305	0.96984918
Cops3	547598.987	594100.463	431706.923	223683.12	218716.652	202181.059	252873.016	224348.268	160642.896	537574.941	448714.296	639170.083	2.54828437	0.40967219
Kcna3	462023.825	515222.7	334114.577	467834.403	459995.186	588629.4	547834.493	291897.795	381059.197	1313198	1340539.14	1639167.88	3.51649325	1.15640077
Anks1a	372812.607	340069.272	284374.997	108178.568	132591.595	114564.484	100026.778	106097.37	98348.5707	592404.133	230065.636	373278.256	3.92727477	0.35631206
Rala	432706.847	277897.325	489330.517	1817.42714	1536.94577	8895.96969	3926.71099	5407.92952	2615.30871	796538.021	349148.135	534717.262	140.62013	0.01020917
Adcy6	11291.6308	5193.7949	13917.362	5365.0999	5784.68073	6476.84808	7360.21743	7743.75592	5303.28054	17817.6126	11782.9656	12824.9822	2.0789451	0.57977015
Cmpk2	26306.5386	18247.4866	19323.4546	23844.5666	25166.0014	17491.0084	22840.8871	23101.0389	28464.0834	48636.364	33373.4233	41668.1889	1.66220413	1.04108015
Crtc1	861695.135	665289.314	994880.349	518433.467	710836.023	545939.087	245710.658	309591.089	328205.134	1600071.76	1317029.9	1867868.2	5.41588296	0.70392694
Znf532	248347.745	293021.564	247062.262	228321.902	220397.238	151252.592	148604.305	248250.911	284391.606	526535.943	861883.674	657644.937	3.00341152	0.76096868
Sik3	732896.605	530337.404	734514.031	878356.386	714632.418	923370.846	685112.999	406343.878	572745.462	1404644.71	1261930.48	1636066.87	2.58540801	1.25959811
Gramd2b	249072.737	255900.057	301157.504	3700.31181	3934.76848	4264.22717	2980.54136	4085.57995	2120.99807	134978.259	124214.211	150568.854	44.6017197	0.01476102
Efr3b	1537672.67	1441476.53	1359457.39	1037868.3	753296.44	817201.778	591279.175	590575.161	501206.755	1320302.59	1732992.24	1341157.84	2.61098821	0.60119913
Nexn	397222.696	509318.8	498314.758	290823.373	230523.153	230002.34	159040.357	164379.12	191651.548	700947.6	550493.586	684790.85	3.75915542	0.53482259
Nav3	109160.96	83928.8531	91587.2237	109540.401	99861.8695	82483.7523	102395.773	93712.0289	68665.6925	408548.314	332816.466	484623.861	4.63032995	1.02532338
Cyld	270774.397	302075.317	323166.448	388947.593	330983.435	454264.112	384766.21	380215.733	459345.138	725262.767	611191.49	821440.227	1.76251471	1.31046201
Snph	330863.581	265073.428	382678.059	23701.2312	16874.3376	21936.9306	42507.8089	34597.9688	36966.2655	224131.584	264373.66	242118.653	6.40493391	0.06387854
Ap4e1	892087.923	511766.647	982765.373	846797.05	753816.022	1027577.94	379972.576	590742.218	527923.334	2380957.22	1724031.78	2002967.72	4.07567151	1.10121891
Negr1	1769591.6	1407608.74	1859827.36	769471.37	724047.244	638845.609	551037.239	867956.055	783320.686	1986538.71	1980667.3	2617815.18	2.99004649	0.4233378
Rdh13	192469.068	150574.097	162216.359	3624.0642	7149.94743	3031.8855	86622.6944	152707.388	154413.811	239421.245	307804.814	254645.057	2.03652966	0.02732437
Setd1b	359851.634	221796.785	408803.344	728253.765	574646.805	443013.571	124456.345	378542.428	311208.148	1356733.98	881435.106	1677283.6	4.808916	1.76274525
Plekho2	1122363.35	1207808.25	1305713.3	685471.583	806459.178	833257.883	547268.64	820041.66	870295.928	2203399.96	1541152.3	1768688.07	2.46390105	0.63951107
Abcf3	414671.958	493246.893	483051.995	354079.286	472416.411	465608.452	298541.757	289989.508	250354.281	727754.811	544285.677	698006.973	2.34841031	0.92892252
Rem2	26407.7571	12654.1208	31307.8703	9197.82824	5205.98945	3212.6846	14145.6784	23863.3269	23218.3532	43126.1477	42522.265	52952.7899	2.26371357	0.25034198
Ndufa8	88402.6885	81720.3535	92286.0799	52720.051	55400.9754	60235.0152	46695.9072	68125.915	63292.5323	101624.457	98054.1948	84402.5203	1.59493699	0.64157846
Rnf14	2091.83848	1951.27342	6273.80463	1396.33295	1305.05988	1993.45231	1477.79523	1256.98688	1309.57431	348711.235	393238.377	439853.69	292.210473	0.45506282
Cdh13	16611.8282	14994.3402	14086.1046	9944.27613	13489.9447	11425.6235	2604.43147	2250.73504	2505.2369	19524.7661	31166.8767	24108.9717	10.1625699	0.76292646
Dctn6	263798.086	306325.42	289898.823	118306.587	101951.257	145471.286	126528.462	158193.955	116247.094	243186.735	325128.712	185228.416	1.87930464	0.42525539
Pacsin2	33601.3763	44741.0387	46989.6769	20122.8864	22401.8824	31780.7554	10913.3443	35227.1193	18022.2946	102717.573	64266.7686	64460.6205	3.6071542	0.5928691

High confidence lost interactions<br>*WT_EZR > GS_EZR & WT_EZR > WT_CYTO*														
	**Normalized peptide intensity per sample**												**Fold change**	
	WT_CYT_1	WT_CYT_2	WT_CYT_3	WT_EZR_1	WT_EZR_2	WT_EZR_3	GS_CYT_1	GS_CYT_2	GS_CYT_3	GS_EZR_1	GS_EZR_2	GS_EZR_3	GS_EZR_v_G	WT_EZR_v_W
Cul4b	840129.702	972404.01	940107.136	1551573.21	1589893.53	1646040.85	1031671.32	1028872.56	1056198.56	842625.601	896988.951	995135.027	0.87743843	1.73924164
Kiaa1522	59997.8	61220.4345	63197.5838	179432.167	126558.346	197894.418	44056.5668	10728.0556	10874.7344	36199.323	26312.0315	32234.0412	1.44298391	2.73233031
Myo1e	10413.5898	18245.3382	12139.0903	35629.0862	37314.7198	30779.7418	19400.5535	9398.30634	7766.68066	16976.3021	16419.9684	10351.3237	1.19641591	2.54236731
Ap1b1	2533236.7	2312981.33	2456883.45	5481963.63	6693604.79	4709944.73	2824815.32	2646851.34	2844827.38	3451916.46	3012233.66	2297157.47	1.0534857	2.31210167
Prkag1	4738.73216	10971.2844	4197.16035	445220.119	196139.741	299017.299	215489.053	86789.3724	74930.0496	8542.29161	9050.7385	9973.00721	0.07307905	47.2380973
Itpr1	425774.194	411128.554	355151.198	846808.522	880333.781	875984.643	653677.83	622879.579	485463.321	571681.126	530417.345	477933.107	0.89671566	2.1837325
Mag	290674.972	426031.282	165440.531	2160840.38	2824268.51	1472812.29	1818891.44	1704502.85	1553469.41	260233.156	415717.631	414020.167	0.21469376	7.32068777
Irs1	53307.1132	61595.2769	60788.3296	230022.858	299547.195	296315.413	35633.0169	49886.5817	55810.7607	47217.0747	63305.0298	111377.499	1.57007741	4.70079164
Adss2	720996.721	671572.543	620569.407	1423804.6	1524364.25	1250165.22	1237392.55	739122.335	943778.8	855176.839	758513.558	909117.622	0.86388846	2.0854669
Mdga2	30377.4892	45409.4265	28994.24	70343.3175	59040.7437	63902.7165	93178.6855	55259.6339	79657.0248	29104.0166	44288.7512	24917.7224	0.43100612	1.84467117
Sbds	528254.533	556383.935	480138.165	748518.743	816239.984	861676.685	826947.406	770015.667	760278.471	497937.603	519860.526	420912.202	0.61033641	1.55065928
Arvcf	3193.05241	3780.07544	3224.07973	127695.76	66849.1229	81153.0076	41459.7116	4446.71424	5964.31855	1097.09899	4474.18424	1753.72455	0.14121655	27.0366067
Lamb2	6427.05032	5823.45393	1113.72829	63917.0632	36465.1084	48595.9872	67915.7376	4193.48437	1194.0406	7596.64494	2531.44461	1781.38126	0.1624685	11.1475282
Mlec	16107.8476	27239.7323	13798.0023	581831.904	558764.221	478240.893	38701.133	33062.5484	34134.0637	37287.0804	31632.6037	54642.0217	1.16680205	28.3282969
Tppp	705675.621	1025872.37	594707.013	1871545.37	2074858.75	1741110.38	1320811.76	1189384.64	1007430.33	703581.146	795724.395	1076006.05	0.73211622	2.44492306
Naa15	277852.787	219730.177	192093.237	366283.476	441499.545	354004.663	415959.475	173505.172	142051.948	195190.118	215158.073	195365.698	0.82802481	1.68454078
Cpt1c	455571.089	425794.178	443476.842	984516.183	1152211.66	1080295.35	718076.074	596365.433	494581.882	539212.932	556067.66	564669.796	0.91759476	2.42823139
Snx16	1736.25603	4118.56042	1756.12518	59411.8925	31741.6881	66108.3549	77654.9822	3218.58979	1605.45585	7613.06644	1415.50214	2895.75512	0.14457401	20.6626122
Nedd4l	750061.719	782516.328	625224.267	1195574.39	1322261.37	1225960.66	1303233.46	813956.874	786369.575	834306.065	749286.081	734007.414	0.79819244	1.73500436
Sh2d5	210914.129	174542.745	90892.088	495428.481	487203.527	551337.214	224496.082	164784.706	103148.668	117431.727	88874.322	159621.719	0.74310698	3.2202636
Svil	5833.87096	22001.1584	7113.91999	259601.981	348720.581	286637.554	156545.593	103036.875	69477.9747	8720.44321	9180.76974	12426.7554	0.09216534	25.6076401
Exoc1	470548.226	374056.284	369288.589	721345.09	727667.336	821161.056	482609.326	226235.725	263077.443	426364.955	520223.665	427677.972	1.41396727	1.87015931
Gldc	6397.99816	2724.77537	3867.27644	26121.6211	25454.3972	21811.8913	35326.6381	23268.2954	24480.0762	5180.41632	8480.71038	2539.98571	0.19501788	5.64954791
Hgs	101094.282	52162.4338	97658.502	345415.302	413077.434	399786.937	296574.033	410881.84	410480.27	69274.2563	71046.2471	58942.5832	0.17824192	4.61621931
Cyb5b	1159.79423	7481.73391	4102.08584	417029.787	527534.265	718763.334	4634.77873	7416.24639	1757.13751	7557.00668	1527.30109	2961.08074	0.87233826	130.522424
Dph7	27942.458	45362.2943	27462.5237	55320.5487	75817.3543	51446.8295	56676.9301	23323.6424	30089.2816	36199.5763	33918.7935	36985.4217	0.97287613	1.81194471
Ppil2	1100.20288	1317.89901	1160.09749	13992.5628	12648.4958	8955.62556	16049.6738	20901.92	27261.2567	2903.09217	5635.54119	3566.57545	0.18851692	9.94821151
Atg7	54503.6978	43581.7233	37603.8699	130813.572	113523.79	65013.3437	154353.739	66352.631	48364.2048	39490.8155	57048.2069	36430.9274	0.49418243	2.27984613
Tnip1	9219.38489	10718.9648	7494.3718	15309.1525	19618.1293	18244.2075	8973.59834	2830.67182	7613.94323	12241.2746	11224.5851	10202.4934	1.73385432	1.93825061
Dpp6	17570.9103	28403.0463	16270.475	53610.7229	87916.6021	67562.1485	63211.5504	32013.3992	33224.1339	20773.1043	23300.8948	26641.4963	0.55053328	3.35916753
